# Polymerization-mediated SRFR1 condensation in upper lateral root cap cells regulates root growth

**DOI:** 10.1093/plcell/koaf292

**Published:** 2025-12-30

**Authors:** Jianbin Su, Xianjin Xu, Joshua S Baik, Leland J Cseke, Katherine Rodriguez-Lukey, Sean K Whittier, Ruimei Zhou, Zhengzhi Zhang, Zackary K Dietz, Bing Yang, Shi-You Chen, William D Picking, Xiaoqin Zou, Walter Gassmann

**Affiliations:** Division of Plant Science and Technology, University of Missouri, Columbia, MO, United States; Interdisciplinary Plant Group, University of Missouri, Columbia, MO, United States; Christopher S. Bond Life Sciences Center, University of Missouri, Columbia, MO, United States; Dalton Cardiovascular Research Center, University of Missouri, Columbia, MO, United States; Department of Physics and Astronomy, University of Missouri, Columbia, MO, United States; Department of Biochemistry, University of Missouri, Columbia, MO, United States; Institute for Data Science and Informatics, University of Missouri, Columbia, MO, United States; Division of Plant Science and Technology, University of Missouri, Columbia, MO, United States; Interdisciplinary Plant Group, University of Missouri, Columbia, MO, United States; Christopher S. Bond Life Sciences Center, University of Missouri, Columbia, MO, United States; Division of Plant Science and Technology, University of Missouri, Columbia, MO, United States; Interdisciplinary Plant Group, University of Missouri, Columbia, MO, United States; Christopher S. Bond Life Sciences Center, University of Missouri, Columbia, MO, United States; Christopher S. Bond Life Sciences Center, University of Missouri, Columbia, MO, United States; Christopher S. Bond Life Sciences Center, University of Missouri, Columbia, MO, United States; Department of Veterinary Pathobiology, University of Missouri, Columbia, MO, United States; Department of Surgery, University of Missouri, Columbia, MO, United States; Division of Plant Science and Technology, University of Missouri, Columbia, MO, United States; Interdisciplinary Plant Group, University of Missouri, Columbia, MO, United States; Christopher S. Bond Life Sciences Center, University of Missouri, Columbia, MO, United States; Christopher S. Bond Life Sciences Center, University of Missouri, Columbia, MO, United States; Department of Veterinary Pathobiology, University of Missouri, Columbia, MO, United States; Division of Plant Science and Technology, University of Missouri, Columbia, MO, United States; Interdisciplinary Plant Group, University of Missouri, Columbia, MO, United States; Christopher S. Bond Life Sciences Center, University of Missouri, Columbia, MO, United States; Donald Danforth Plant Science Center, St. Louis, MO, United States; Department of Surgery, University of Missouri, Columbia, MO, United States; Christopher S. Bond Life Sciences Center, University of Missouri, Columbia, MO, United States; Department of Veterinary Pathobiology, University of Missouri, Columbia, MO, United States; Dalton Cardiovascular Research Center, University of Missouri, Columbia, MO, United States; Department of Physics and Astronomy, University of Missouri, Columbia, MO, United States; Department of Biochemistry, University of Missouri, Columbia, MO, United States; Institute for Data Science and Informatics, University of Missouri, Columbia, MO, United States; Division of Plant Science and Technology, University of Missouri, Columbia, MO, United States; Interdisciplinary Plant Group, University of Missouri, Columbia, MO, United States; Christopher S. Bond Life Sciences Center, University of Missouri, Columbia, MO, United States

## Abstract

Primary root growth, regulated by internal hormone signals, adapts to external factors such as water availability, soil compactness, and microbial interactions. An essential step in root growth consists of cell divisions in the meristem, with the outermost root cap layer thought to provide protection. However, recent studies reveal that lateral root cap (LRC) cells control meristem size and lateral root initiation. In this study, we identified an upper LRC-specific protein condensation mechanism involving SUPPRESSOR of rps4-RLD1 (SRFR1) that governs root growth and show that growth conditions and hormone treatment dynamically modulate condensate accumulation. SRFR1 condensate formation is driven by its plant-associated N-terminal tetratricopeptide repeat (PANT) polymerization domain and fine-tuned by the adjacent intrinsically disordered region 1 (IDR1). Mutational and biophysical analyses show that IDR1's zwitterionic nature is essential for its regulatory role, acting as a chaperone to promote PANT polymerization at low temperatures while preventing aggregation at high temperatures. This enables SRFR1 condensate formation across a wide temperature range. Notably, the zwitterionic IDR1 can be functionally substituted by zwitterionic dehydrins. Shifting IDR1 toward a negative state impairs, whereas a positive shift enhances SRFR1 condensation and further improves root growth. The association of zwitterionic IDRs with polymerization domains is common, suggesting that this mechanism broadly prevents irreversible aggregation and promotes physiological polymerization under varying temperatures.

## Teaser

The ability to assemble into condensates in a specific root cell type is necessary for the protein SRFR1, which is soluble in most other cell types, to regulate root growth.

## Introduction

Biomolecular condensates play key roles in various developmental, pathological, and stress response processes (Brangwynne et al. 2009; Li et al. 2012a; Banani et al. 2017; Shin and Brangwynne 2017; Fang et al. 2019; Powers et al. 2019; Huang et al. 2021; Mittag and Pappu 2022). Protein constituents of such condensates may be removed from activity or conversely provide accelerated functions with molecular crowding (Folkmann et al. 2021; Huang et al. 2021). Additionally, proteins sequestered into biomolecular condensates can be protected from degradation for a more rapid re-engagement with cellular processes (Saad et al. 2017) or conversely lead to efficient ubiquitination and protein degradation (Zavaliev et al. 2020). These processes are not mutually exclusive and allow a fine-tuning of responses.

The formation of biomolecular condensates is often driven by multivalent interactions, which can arise from either intrinsic sticker–sticker interactions encoded in intrinsically disordered regions (IDRs) or from emergent polymerization of folded domains (Choi et al. 2020; Mittag and Pappu 2022). Considerable studies have focused on IDRs as the drivers for biomolecular condensate formation (Mittag and Pappu 2022). However, less studied are principles of polymerization- or oligomerization-mediated biomolecular condensate formation. Bienz proposed 2 principles underlying head-to-tail type polymerization in biomolecular condensate formation (Bienz 2020): (i) polymerization must be spontaneous and reversible, leading to short and dynamic filaments. Indeed, the DIX domain of Disheveled 2 (DVL2) spontaneously forms reversible short filaments in vitro (Schwarz-Romond et al. 2007). (ii) Because head-to-tail polymerization only gives rise to 1D filaments, Bienz reasoned that at least one additional interaction is required to cross-link 1D filaments to form a 3D network.

This notion is supported by the fact that the DEP domain of DVL2 is capable of dimerizing, leading to cross-linked DIX-induced 1D filaments (Bienz 2020). Beyond the DVL2 model, condensate formation driven by oligomerization of coiled-coil domains in bacterial proteins, such as Crn1 and PopZ, is modulated by adjacent IDRs (Lasker et al. 2022; Han et al. 2023). As an entropy-driven process, polymerization/oligomerization is highly temperature sensitive, exhibiting a high activation energy barrier that impedes initiation at low temperatures and promotes excessive, often irreversible aggregation at high temperatures (Brangwynne et al. 2015; Narayanan et al. 2019; Rodriguez Gama et al. 2021; Mittag and Pappu 2022). The mechanisms governing polymerization/oligomerization-mediated condensate formation across a broad temperature range remain poorly understood. Additionally, while thermosensitive condensates have been extensively studied (Nott et al. 2015; Hahm et al. 2020; Jung et al. 2020; Zhu et al. 2021; Chen et al. 2022; Du et al. 2024), the mechanisms ensuring the stability of condensates across wide temperature ranges are less explored.

In materials science, pH-sensitive, temperature-invariant diblock copolymers have been synthesized by linking a lower critical solution temperature (LCST)-type block with an upper critical solution temperature (UCST)-type zwitterionic polymer (Morimoto and Yamamoto 2021). Moreover, the conjugation of zwitterionic synthetic polymers has been used to enhance the thermal stability of protein enzymes, providing both cryoprotective and heat-protective effects (Keefe and Jiang 2011; Cummings et al. 2014). These findings suggest that zwitterionic polypeptides, particularly zwitterionic IDRs or zwitterionic intrinsically disordered proteins (IDPs), may exhibit analogous chaperone-like functions in biological systems. Zwitterionic IDRs/IDPs are characterized by their balanced enrichment in oppositely charged residues, with charge stoichiometry either equal or weighted toward a positively or negatively charged state (Das and Pappu 2013; Blackman et al. 2019). A notable example is the late embryogenesis abundant (LEA) protein family, many members of which are zwitterionic. However, it remains unclear whether the zwitterionic nature of LEA proteins contributes to their functional properties, such as providing cryoprotective and heat-protective functions for various client proteins (Hernandez-Sanchez et al. 2022; Smith and Graether 2022).

The length of the primary Arabidopsis (*Arabidopsis thaliana*) root is determined by the size of the root apical meristem (RAM) (Beemster and Baskin 1998; Dello Ioio et al. 2008; De Nittis et al. 2025). A small number of undifferentiated stem cells form the quiescent center (QC) that generates files of daughter cells destined to form the cylindrical arrangement of epidermal, cortical, and vascular cells. Rapid cell divisions in these files result in root tissue consisting of unelongated cells that form the root meristem. At the transition zone (TZ), these cells start to elongate, resulting in rapid growth of the root. Placement of the TZ is therefore a tightly regulated and choreographed developmental process mainly determined by the plant hormones auxin and cytokinin (CK) (Di Mambro et al. 2017; De Nittis et al. 2025). Polar auxin transport from the shoot to the root tip occurs along the central cylinder of the root and at the tip reverses upwards in the outer cell layers (Grieneisen et al. 2007). This maximum of auxin concentration at the root tip promotes cell division and prevents cell differentiation (elongation). CK counteracts this upward movement of auxin by downregulating PINs, polar auxin transporters responsible for moving auxin up the outer cell columns (Dello Ioio et al. 2008). This antagonism leads to a switch from cell proliferation (auxin dominant) to cell elongation (CK dominant) at the TZ.

The lateral root cap (LRC), a cell layer external to the epidermis at the very tip of the root whose cells eventually undergo programmed cell death (PCD), was shown to serve as a strong auxin sink as a result of LRC-specific expression of *GH3.17* and *PIN5* (Di Mambro et al. 2019). GH3.17 inactivates auxin by conjugation to aspartate and glutamate, while the auxin transporter PIN5 moves auxin out of the cytoplasm into the ER, where it is not biologically active. Because of the facile movement of auxin down concentration gradients, the upper LRC cells establish a zone of minimal auxin concentrations across the root layers, defining the TZ (Di Mambro et al. 2019). Indeed, overexpression of GH3.17 and PIN5 shifts the TZ toward the root tip compared with wild-type (Di Mambro et al. 2019).

Additional plant hormones impinge on the auxin-CK interaction and root growth. For example, ethylene (ET) promotes the expression of auxin transport and biosynthesis genes. The resulting increase in auxin concentrations in the elongation zone delays cell elongation and inhibits root growth (Vaseva et al. 2018; Qin et al. 2019; Yamoune et al. 2024). In contrast, gibberellic acid (GA) inhibits CK-induced gene expression at the root tip and therefore leads to longer root meristems, ultimately promoting root growth (Shtin et al. 2022). It has been proposed that upper LRC cells (LRCs), as the central regulators of TZ placement, are the main location for integration of internal (developmental) and external (environmental) signals to fine-tune adaptation of root development (Salvi et al. 2020).

In this study, we found that the Arabidopsis SUPPRESSOR of rps4-RLD1 (SRFR1) protein specifically forms biomolecular condensates in the upper LRCs. We further show that the extent of SRFR1 condensation is sensitive to growth conditions and hormone treatments but remains stable across a broad temperature range (0 to 33 °C). We identified a condensation module governing SRFR1 condensate formation, consisting of a polymerization domain (PD) that drives assembly and an adjacent IDR that finetunes condensate dynamics. The zwitterionic IDR enables SRFR1 condensate formation across a wide temperature range. Further, shifting the zwitterionic IDR to a positively charged state enhances condensate formation and promotes root growth.

## Results

### SRFR1 regulates primary root growth

**Figure 1.**
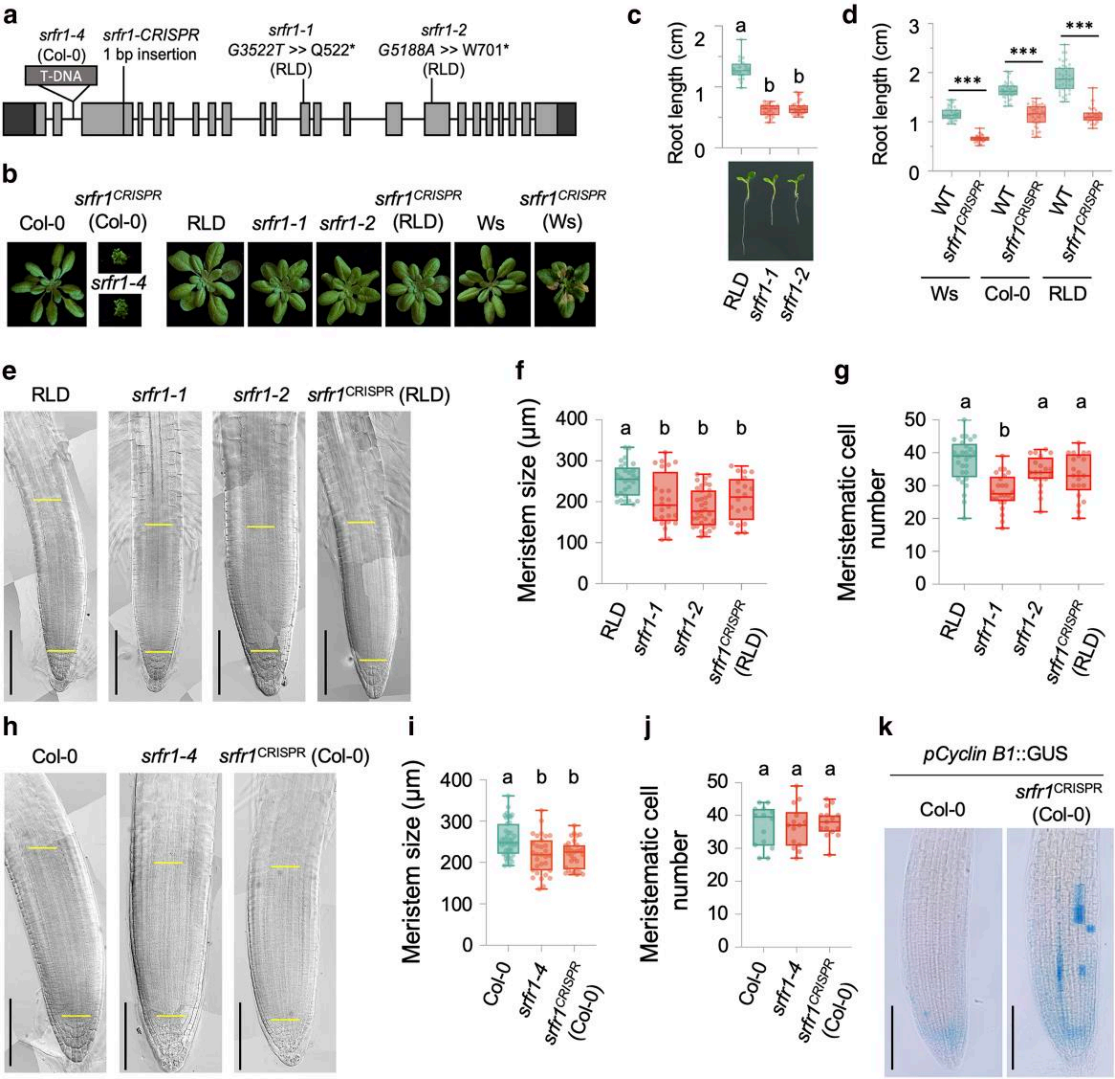
SRFR1 regulates primary root development. a) Schematic diagram of the *SRFR1* gene structure. *srfr1-1* and *srfr1-2* (RLD background) and *srfr1-4* (SAIL_412_E08, Col-0 background) have been described in previous studies (Kwon et al. 2009; Kim et al. 2010). *srfr1^CRISPR^* mutants in Col-0, RLD, and Ws backgrounds all harbor a 1 bp insertion at the same site. b) Rosette phenotype of soil-grown *srfr1* mutants in Col-0, RLD, and Ws backgrounds. Here and in subsequent figures, depicted plants of the same genetic background were grown together and recorded at the same time. c) Primary root length of *srfr1-1* and *srfr1-2*. Primary root length is measured with 6-d-old seedlings, *n* = 20 to 28. d) Primary root length of *srfr1^CRISPR^* mutants in Col-0, RLD, and Ws background. Root length was measured with 6-d-old seedlings, *n* = 40. e to g) Root apical meristem size and meristematic cell number of RLD, *srfr1-1*, *srfr1-2*, and *srfr1^CRISPR^* (RLD) measured with 6-d-old seedlings. *n* = 20 to 28. h to j) Root apical meristem size (*n* = 30 to 43) and meristematic cell number (*n* = 14 to 15) of Col-0, *srfr1-4*, and *srfr1^CRISPR^* (Col-0) measured with 6-d-old seedlings. The individual samples shown in panels (e) and (h) were assembled from multiple images of the same root. k) Representative images of Cyclin B1-GUS activity of Col-0 and *srfr1^CRISPR^* (Col-0) in 6-d-old seedlings. For c, d, f, g, i, and j), all data points are shown in box-and-whisker plots. Boxes indicate the interquartile range, with center lines denoting the median. Whiskers extend to the minimum and maximum values of the dataset. *** indicates *P* < 0.001, unpaired Student's *t*-test, and letters denote statistically different groups (ordinary one-way ANOVA, Tukey-Kramer grouping).

In our previous work, we demonstrated that SRFR1 is a negative regulator of effector-triggered immunity (ETI) (Kwon et al. 2009; Kim et al. 2010; Bhattacharjee et al. 2011). Our previous studies found that *srfr1* mutants exhibited distinct shoot phenotypes in Col-0 and RLD accessions (Fig. 1, a and b). For example, *srfr1-4* showed severe stunting mediated by the Col-0 background-specific SNC1 protein (Fig. 1b) (Kim et al. 2010; Bhattacharjee et al. 2011). In this study, we found that the Wassilewskija-0 (Ws-0) background *srfr1* CRISPR mutant grew normally during the first 3 wk, then cell death initiated at the petioles and progressed toward the leaf tips (Fig. 1b). Apart from the *SNC1*-mediated shoot stunting in Col-0, we observed a modest reduction in shoot growth in RLD and Ws-0 *srfr1* mutants (Fig. 1b) (Kim et al. 2010). In addition, all *srfr1* mutants in 3 different accessions, including the newly CRISPR-generated alleles, exhibited a shortened primary root (Fig. 1, a, c, and d).

To investigate how *srfr1* mutations lead to a shortened primary root, we quantified the meristem length and numbers of meristematic cells in *srfr1* mutants in both RLD and Col-0 backgrounds. As expected, all *srfr1* mutants exhibited reduced meristem sizes (Fig. 1, e, f, h, and i). Although we observed a trend of decreased meristematic cell numbers in all *srfr1* mutants, a significant reduction in meristematic cell numbers was only detected in *srfr1-1* (Fig. 1, g and j). The lowest meristematic cell number in *srfr1-1* correlates with the highest expression of *ACS* genes (Supplementary Figure S1a). To further examine the effects of *srfr1* mutations on meristematic cell division, we generated *srfr1 CRISPR* mutants in the *pCyclin B1::GUS* reporter line. By screening multiple lines, we identified mutants carrying the same single-base (A) insertion in *SRFR1*. Interestingly, *pCyclin B1::GUS srfr1^CRISPR^* seedlings showed increased rather than reduced GUS staining (Fig. 1k). Together, these results suggest that the shortened root and reduced meristem size in *srfr1* mutants primarily result from impaired cell elongation rather than defective cell division in the meristem.

**Figure 2.**
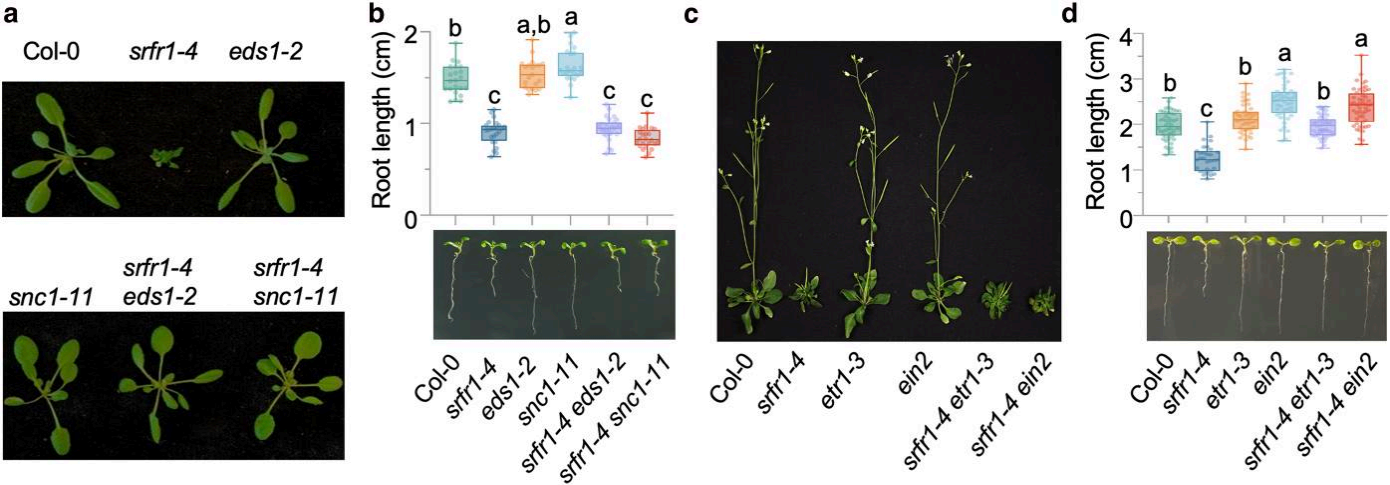
The short primary root phenotype of *srfr1-4* is dependent on ethylene signaling. a and b) The stunted shoot phenotype a), but not the short primary root phenotype b), of *srfr1-4* is dependent on *EDS1* and *SNC1*. Primary root length is measured with 6-d-old seedlings, *n* = 21 to 27. c and d) The stunted shoot phenotype c), but not the short primary root phenotype d), of *srfr1-4* is independent of ethylene signaling. Primary root length is measured with 7-d-old seedlings, *n* = 31 to 52. For b and d), all data points are shown in box-and-whisker plots. Boxes indicate the interquartile range, with center lines denoting the median. Whiskers extend to the minimum and maximum values of the dataset. Letters denote statistically different groups (ordinary one-way ANOVA, Tukey-Kramer grouping).

The accession-independent short primary root phenotype of *srfr1* mutants suggests that regulating primary root growth is a general function of SRFR1 that is independent of ETI. Indeed, *srfr1-4 snc1-11* and *srfr1-4 eds1-2* double mutants still exhibited short primary roots, in contrast to the reversion of shoot stunting by mutations in *EDS1* or *SNC1* (Fig. 2, a and b) (Bhattacharjee et al. 2011; Garner et al. 2021). Further, our results suggest that regulation of primary root growth by SRFR1 is dependent on ethylene signaling. The short root phenotype of these mutants was largely rescued by both aminoethoxyvinylglycine (AVG) and silver nitrate (AgNO3), 2 inhibitors of ethylene biosynthesis (Supplementary Figure S1, b and c). Consistent with this observation, several *ACS* genes encoding enzymes that catalyze the rate-limiting step of ethylene biosynthesis, particularly *ACS7* and *ACS9*, were expressed at higher levels in the root tissues of *srfr1* mutants (Supplementary Figure S1a). To provide genetic evidence that the short primary root phenotype results from elevated ethylene responses, we crossed *srfr1-4* with *etr1-3* and *ein2*, 2 key ethylene signaling mutants. In contrast to the *srfr1-4 snc1-11* and *srfr1-4 eds1-2* double mutants, *srfr1-4 etr1-3* and *srfr1-4 ein2* exhibited restored primary root lengths equivalent to those of *etr1-3* and *ein2* single mutants, respectively, while retaining the stunted shoot phenotype characteristic of *srfr1-4* (Fig. 2, c and d). Additionally, SRFR1 high-expression lines showed a longer primary root compared with wild-type (Supplementary Figure S1d). These results suggest that SRFR1 plays an essential role in preventing the overactivation of ethylene responses, thereby facilitating optimal root growth.

### SRFR1 forms condensates in upper LRC cells

**Figure 3.**
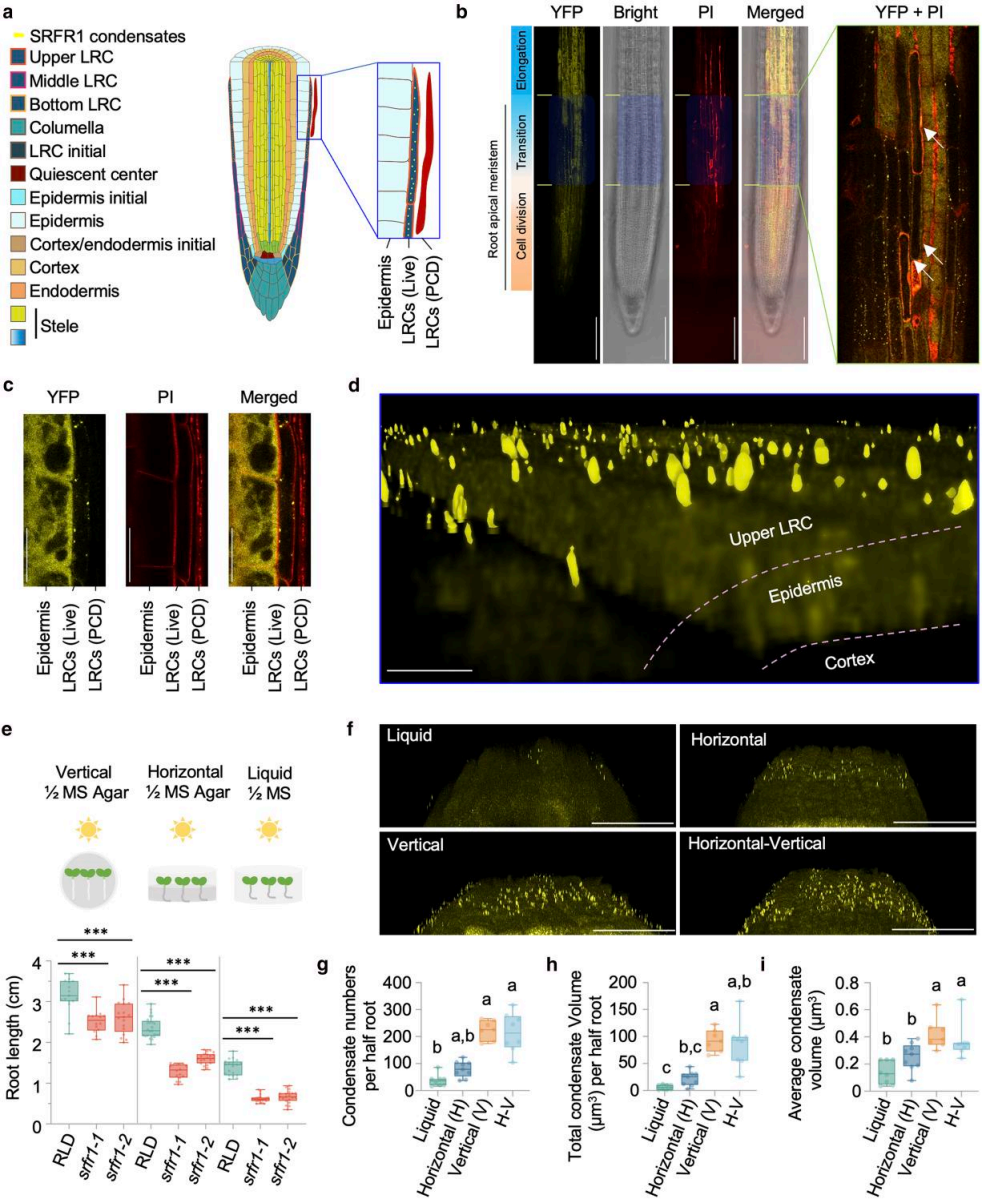
SRFR1 forms condensates in upper lateral root cap (LRC) cells, and their accumulation is sensitive to growth conditions. a) The diagram demonstrates the specific localization of SRFR1 condensates in upper lateral root cap cells (LRCs). b) Localization of SRFR1 condensates in roots of 6-d-old seedlings. Images were obtained using tile scanning and assembled from 4 surface section images, bar = 100µm. Transparent blue box indicates the upper LRC region; white arrows indicate cells undergoing programmed cell death (PCD). c) Localization of SRFR1 condensates in live upper LRCs. Median optical section images were acquired from roots of 6-d-old seedlings, bar = 20 µm. d) 3D localization of SRFR1 in the upper LRCs. A Z-stack of surface sections in the upper LRC region of 6-d-old seedling roots was taken using confocal microscopy. e) Primary root length of *srfr1-1* and *srfr1-2* is sensitive to growth conditions. Primary root length was measured with 8-d-old seedlings, *n* = 14 to 25. f) Accumulation of SRFR1 condensates in upper LRCs under different growth conditions. Confocal 3D images were taken from the roots of 6-d-old *srfr1-1 YFP-HA-gSRFR1^WT^ #7* seedlings grown under different conditions. Z-stack images were collected by scanning from the root surface to the midsection in intervals of 0.42 µm. Bar =50 µm. For the horizontal–vertical experiment, images were taken 12 h after transfer. Seedlings were fixed with 4% PFA for large-scale 3D imaging. *YFP-HA-SRFR1^WT^* #7 was used for all condensate imaging. g to i) Quantification of SRFR1 condensate number, total condensate volume, and average condensate volume per-half-root under different conditions (*n* = 8 to 10). The average condensate volume was calculated by first averaging the volumes of all condensates within each half-root, followed by averaging per-half-root means across samples. For e and g to i), all data points are shown in box-and-whisker plots. Boxes indicate the interquartile range, with center lines denoting the median. Whiskers extend to the minimum and maximum values of the dataset. *** indicates *P* < 0.001, unpaired Student's *t*-test, and letters denote statistically different groups (one-way ANOVA, Kruskal–Wallis Dunn's post-hoc test).

We next examined the tissue and subcellular localization of SRFR1. We inserted a YFP and an HA tag just before the start codon in a native promoter-driven 9 kb *SRFR1* genomic clone, designated *YFP-HA-gSRFR1^WT^*. SRFR1 displayed a diffuse cytoplasmic distribution in most root tissues, but it formed puncta in upper LRCs (Fig. 3, a to d, Supplementary Figure S2a, Supplementary Videos 1 and 2). In shoot tissues, SRFR1 puncta were frequently observed in developing trichomes and young stomatal cells, whereas they were rarely seen in mesophyll cells (Supplementary Figure S2, b to d). These findings suggest that SRFR1 puncta represent a spatially and temporally distinct tissue-specific biomolecular condensate. Notably, the upper LRC region where SRFR1 condensates form is aligned with the TZ (Fig. 3b). Previous studies have shown that LRCs act as an auxin sink regulating the meristem size (Di Mambro et al. 2019), with PCD of LRCs releasing auxin to promote lateral root initiation above the meristem (Xuan et al. 2016). Thus, we examined multiple roots and found that SRFR1 forms condensates only in live upper LRCs, but not in those heavily stained with propidium iodide (PI) or showing disrupted staining patterns, indicative of cell damage caused by PCD (Fig. 3, b and c). The study found significant changes in both meristematic cell numbers and meristem length by manipulating cytokinin signaling in the entire LRC, encompassing the bottom, middle, and upper regions (Di Mambro et al. 2019). In contrast, SRFR1 condensate formation is restricted to the upper LRC region corresponding with the TZ. This spatially confined condensate accumulation may explain why most *srfr1* mutants exhibit only a slight, but not statistically significant, reduction in meristematic cell numbers. Morphologically, SRFR1 condensates frequently exhibited column-like or elongated structures of varying lengths and diameters (Fig. 3d).

### Accumulation of SRFR1 condensates is dynamically regulated by both environmental and developmental cues

Root growth in plants is highly plastic, modulated by both internal developmental signals and external environmental cues (De Nittis et al. 2025). In a preliminary assessment of growth conditions, we observed that root growth of *srfr1* mutants compared with wild-type plants was reduced by approximately 19.5% when seedlings were grown vertically on ½ MS agar plates with whole roots exposed to air. This reduction was more pronounced (∼39%) when seedlings were grown horizontally with roots penetrating the growth media and became more severe (54.6% reduction) when grown in liquid ½ MS medium (Fig. 3e). We next examined SRFR1 condensate accumulation in the upper LRCs of the YFP-HA-SRFR1^WT^ line #7 under different growth conditions. Fewer condensates were detected in seedlings grown in liquid medium, with increased accumulation under horizontal growth and the highest levels observed under vertical growth (Fig. 3f).

As primary root growth rates follow the order vertical > horizontal > liquid (Fig. 3e), this pattern suggests a positive correlation between SRFR1 condensate accumulation and primary root growth. To quantify SRFR1 condensate accumulation, we measured condensate number, total condensate volume, and average condensate volume. Due to the limited scanning depth of confocal microscopy, we quantified condensates per-half-root, scanning from the outer surface to the midsection. Given the column-like morphology of SRFR1 condensates, we approximated their volumes as cylinders. Condensate number, total condensate volume, and average condensate volume consistently increased in the order vertical > horizontal > liquid (Fig. 3, f to i). Remarkably, only after 12 h of transferring seedlings to vertical plates, SRFR1 condensate accumulation reached levels comparable to those in continuously vertically grown seedlings (Fig. 3, f to i), indicating dynamic regulation in response to environmental changes.

**Figure 4.**
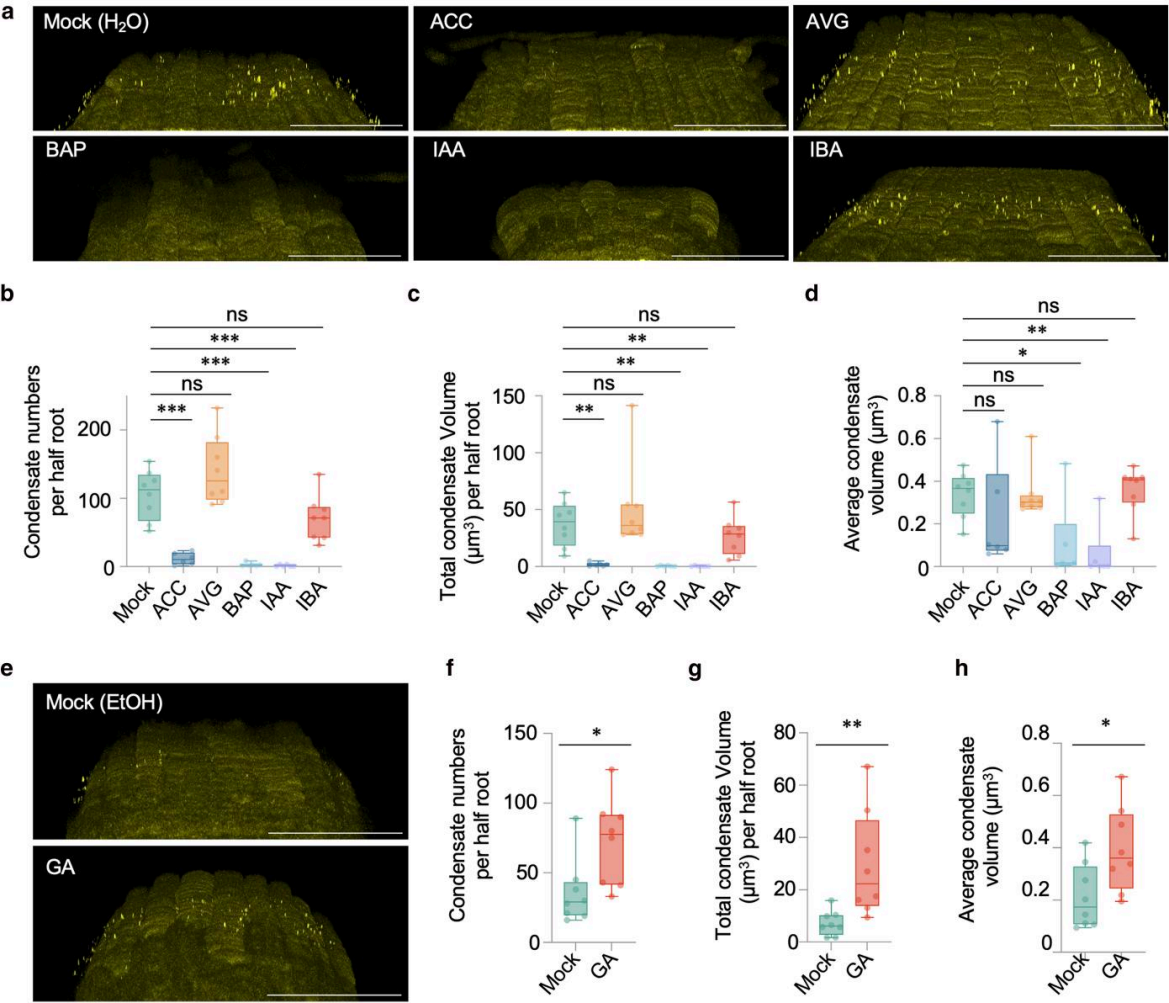
SRFR1 condensate accumulation responds to hormones. a and e) Accumulation of SRFR1 condensates in upper LRCs under different hormone treatments. Confocal 3D images were taken from the roots of 6-d-old *srfr1-1 YFP-HA-gSRFR1^WT^ #7* seedlings grown under different conditions. Z-stack images were collected by scanning from the root surface to the midsection at 0.42 µm intervals. Bar = 50 µm. Seedlings were fixed with 4% PFA 12 h post-hormone treatment for large-scale 3D imaging. d to d and f to h) Quantification of SRFR1 condensate number, total condensate volume, and average condensate volume per-half-root under different conditions (*n* = 6 to 8). The average condensate volume was calculated by first averaging the volumes of all condensates within each half-root, followed by averaging per-half-root means across samples. All data points are shown in box-and-whisker plots. Boxes indicate the interquartile range, with the center line denoting the median. Whiskers extend to the minimum and maximum values of the dataset. *** indicates *P* < 0.001, ** indicates *P* < 0.01 and * indicates *P* < 0.05 (unpaired Mann–Whitney *U* test).

Next, we examined SRFR1 accumulation in response to hormone treatment. SRFR1 condensates were almost completely eliminated when roots were treated with 1-aminocyclopropane-1-carboxylate (ACC), 6-benzylaminopurine (BAP), and indole-3-acetic acid (IAA), while GA treatment promoted condensate formation (Fig. 4, a to h). Treatments with AVG and indole-3-butyric acid (IBA) caused no significant changes after 12-h treatment (Fig. 4, a to d). Because IBA must first be converted to IAA to effect auxin functions in roots (Strader et al. 2011), it is possible that this conversion was insufficient in our experimental conditions. Taken together, these results suggest that the formation and disassembly of SRFR1 condensates are dynamically regulated by both hormonal and environmental cues.

### The PANT-IDR1 module is required for upper LRC SRFR1 condensate formation

**Figure 5.**
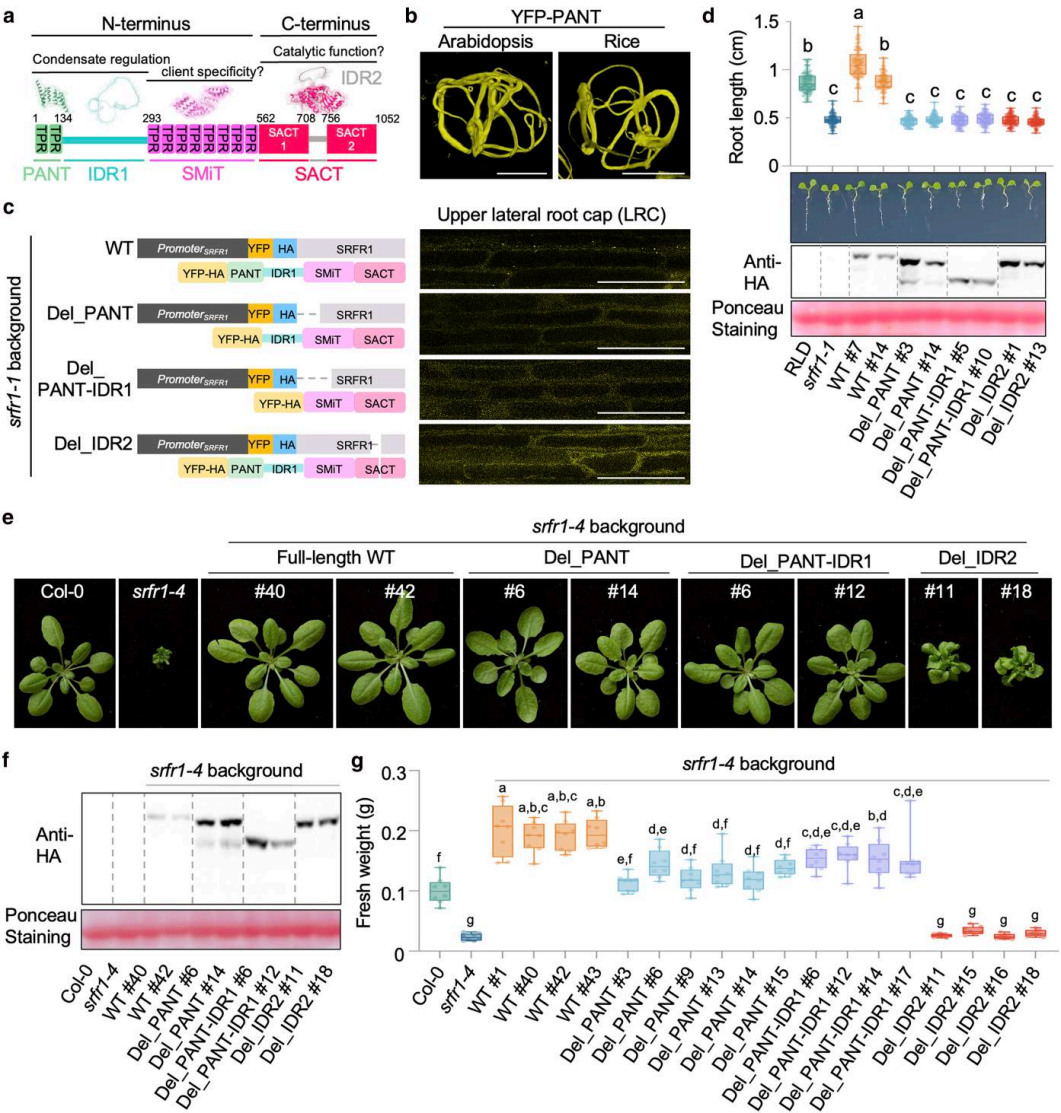
The PANT-IDR1 module is required for upper LRC SRFR1 condensate formation and root growth, but is dispensable for suppressing ETI. a) Schematic diagram of SRFR1 protein architecture. Structures of all domains are derived from AlphaFold2 prediction (AF-F4JS25-1). b) Z-stack images show the 3D localization of PANT polymers in rice and Arabidopsis protoplasts. Bar = 10 µm. c) Subcellular localization of full-length and truncated SRFR1 in upper LRC. Images were taken with roots of 6-d-old seedlings. Single surface sections were taken using the same confocal microscopy settings. Bar = 20 µm. d) Deletion of PANT, PANT-IDR, or IDR2 failed to rescue the *srfr1-1* short primary root phenotype. Primary root length is measured with 6-d-old seedlings, *n* = 63 to 88. Ponceau S staining of the Rubisco large subunit shows equal protein loading. e) Rosette phenotype of 4-wk-old soil-grown YFP-HA-tagged full-length and truncated SRFR1 variants in the *srfr1-4* background. f) Western blot detection of YFP-HA-tagged full-length and truncated SRFR1 expression in the *srfr1-4* background. Ponceau S staining of the Rubisco large subunit shows equal protein loading. g) The PANT-IDR1 module is required for increased SRFR1-mediated biomass. Fresh weights of different genotypes were measured with 4-wk-old soil-grown plants, *n* = 9. For d and g), all data points are shown in box-and-whisker plots. Boxes indicate the interquartile range, with center lines denoting the median. Whiskers extend to the minimum and maximum values of the dataset. Letters denote statistically different groups (Ordinary one-way ANOVA, Tukey-Kramer grouping).

Structural prediction by AlphaFold2 suggests that SRFR1 contains 3 distinct domains and 2 IDRs (Fig. 5a). We designated these domains as the PANT domain, SRFR1 middle TPR (SMiT) domain, and SRFR1-associated C-terminal (SACT) domain. The PANT and SMiT domains are separated by IDR1, while the shorter IDR2 divides the SACT domain into 2 subdomains (Fig. 5a). The PANT domain is conserved in plants but absent in animal SRFR1 orthologs (Supplementary Figure S3a). The SACT domain is conserved in eukaryotes and is consistently found alongside at least an SMiT domain; however, its function remains unknown (Kwon et al. 2009). To investigate which domain is required for SRFR1 biomolecular condensate formation, we expressed individual domains and their combinations in both rice and Arabidopsis protoplasts and human (*Homo sapiens*) HEK293T cells (Supplementary Figure S3, b to e). Surprisingly, the PANT domain alone formed cytoskeleton-like fibrils in Arabidopsis, rice, and human cells (Fig. 5b, Supplementary Figure S3, b to d, and Supplementary Video 3).

Interestingly, when IDR1 was present, PANT fibril formation was suppressed, resulting in only 1 or 2 puncta in rice protoplasts but diffuse localization in Arabidopsis protoplasts and human HEK293T cells (Supplementary Figure S3, b to d). Based on these findings, we hypothesized that SRFR1 condensate formation is driven by PANT polymerization and fine-tuned by IDR1. This hypothesis is further supported by the observation that removing the PANT domain from full-length SRFR1 completely abolished condensate formation, while deletion of IDR1 or IDR2 still allowed puncta formation, albeit less frequently (Supplementary Figure S3, b and d).

We next tested these results from transient overexpression using stable transgenic plants with the *YFP-HA-gSRFR1^WT^* construct (Figs. 3 and 4) as a starting point, since this construct successfully complemented all mutant phenotypes of *srfr1-1* and *srfr1-4* (Fig. 5, c to e), suggesting that the N-terminal YFP-HA tag does not affect SRFR1 function. PANT, PANT-IDR1, or IDR2 were deleted in the *YFP-HA-gSRFR1^WT^* clone (Fig. 5c), and the resulting constructs were used to transform both *srfr1-1* and *srfr1-4*, 2 *SRFR1* mutants in the RLD and Col-0 background, respectively. SRFR1 with deleted PANT, PANT-IDR1, or IDR2 abolished upper LRC SRFR1 condensate formation and failed to complement the short primary root phenotype (Fig. 5, c and d). To our surprise, despite being essential for biomolecular condensate formation, the PANT and IDR1 domains were dispensable for complementing ETI-related phenotypes, such as the severely stunted shoots of *srfr1-4* (Fig. 5, e to g). We noticed that multiple high-expression transgenic lines readily could be obtained for SRFR1^Del_PANT^, SRFR1^Del_PANT-IDR1^, and SRFR1^Del_IDR2^, but not for SRFR1^WT^ (Fig. 5, d and f). Moreover, the protein levels in these truncation or deletion lines were consistently higher than those in SRFR1^WT^ lines, suggesting that SRFR1 protein abundance is tightly regulated and that these domains may play a role in maintaining SRFR1 homeostasis. Although SRFR1 truncation variants lacking the PANT or PANT-IDR1 domains were not as efficient in promoting wild-type-level shoot growth (Fig. 5g), their fresh weights were increased compared with Col-0, possibly due to elevated SRFR1 expression levels. Finally, the 51 amino acid IDR2 (residues 708 to 758) was found to be essential, as SRFR1 lacking IDR2 failed to complement any of the *srfr1* mutant phenotypes (Fig. 5, c to g). In summary, both transient expression in protoplasts and stable transgenic expression in the whole organism suggest that the PANT domain, along with its adjacent IDR1, functions as a condensation module, regulating SRFR1 condensate formation that is required for primary root growth.

### PANT domain-mediated polymerization

**Figure 6.**
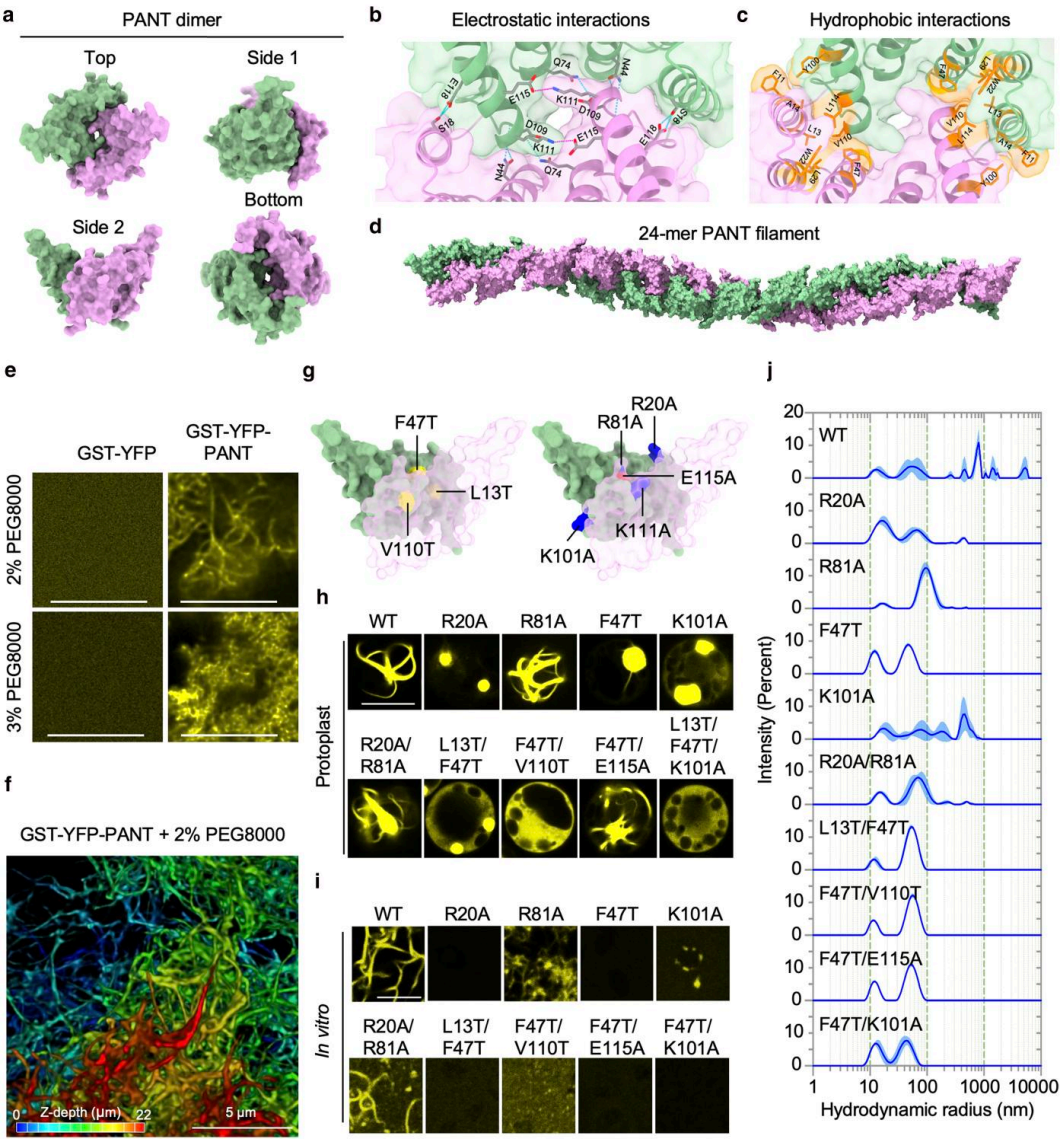
The PANT domain forms fibrils both in vitro and in vivo. a to d) PANT dimer prediction was performed with locally installed AlphaFold v2.2.4 using default settings. Hydrogen bonds are shown as cyan dashed lines, salt bridges as red lines, and hydrophobic interactions as yellow surfaces. The PANT 24-mer was generated using MatchMaker in UCSF Chimera. e) Formation of PANT fibrils and PANT-IDR1 condensates in PBS buffer induced by PEG8000. Thirty micromolars of protein was used for each. Images were taken 3 h post-PEG addition with the same confocal microscopy settings. Bar = 10 µm. f) 3D organization of PANT fibrils in vitro induced by 2% PEG8000. g) Key residues mediating PANT dimer formation are highlighted. Positively and negatively charged residues are colored in blue and red, respectively. Hydrophobic residues are shown in yellow. h) Subcellular localization of PANT dimer- and tetramer-interface variants in rice protoplasts. i) In vitro fibril or condensate formation assay of GST-eYFP-tagged PANT and PANT variants at 15 µM in PBST buffer containing 0.5% PEG8000 (w/v) at room temperature. Images were taken 3 h post-PEG addition using the same confocal microscopy settings. Bar = 10 µm. j) Hydrodynamic radius of GST-eYFP-tagged PANT and PANT variants measured by MADLS at 24 °C using 30 µM protein in PBS solution.

The fibril-like localization pattern of the PANT domain suggests its ability to undergo spontaneous polymerization. To gain insights into PANT polymerization, we used a locally installed AlphaFold2 program to model PANT dimer and tetramer structures. The predicted PANT dimer has a high-confidence score with multiple stabilizing interactions, including a salt bridge between K111 and E115, hydrogen bonds within residue pairs such as S18-E117 and Q74-D109, and hydrophobic interactions involving F11, L13, A14, W22, L29, F47, Y100, V110, and L114 (Fig. 6, a to c). According to the modeled structures, PANT tetramers may exist as ensembles of several structural conformations, potentially corresponding to polymerization behaviors, such as filament elongation, bending, twisting, fractal branching, and bundling (Fig. 6d and Supplementary Figure S4a).

Purified GST-eYFP-tagged PANT domain formed fibrils in vitro when induced by the crowding reagent PEG8000 (Fig. 6e). 3D reconstruction revealed that PANT domains form intricate, fractal-like fibrils in vitro (Fig. 6f and Supplementary Video 4), which closely resemble those observed in protoplasts and human HEK293T cells (Fig. 5b and Supplementary Figure S3, b to d). We then tested these models in vitro and in protoplasts by introducing multiple mutations at the predicted dimer- and tetramer-forming interfaces (Fig. 6g and Supplementary Figure S4a). In rice protoplasts, 58 PANT variants were expressed, and their localization patterns could generally be categorized into 7 groups (Fig. 6h and Supplementary Figure S4b): (i) WT-like fibrils; (ii) short or thin fibrils; (iii) aggregation-like puncta; (iv) hairy puncta with emerging thin fibrils; (v) spherical puncta; (vi) spherical puncta coexisting with diffuse localization, and (vii) completely diffuse localization. We also tested 17 PANT variants in Arabidopsis protoplasts (Supplementary Figure S4c). Many PANT variants exhibited localization patterns similar to those observed in rice protoplasts. However, several variants, including F47T, V110T, K111A, L114T, and F47T/E115A, formed ring-like structures surrounding chloroplasts, suggesting that factors associated with chloroplast outer membranes may serve as nucleation sites for their polymerization. In this context, the heterologous rice protoplast system may better reflect the intrinsic polymerization behavior of PANT variants, possibly due to weaker interactions with rice proteins. This extensive mutational analysis not only confirmed the accuracy of our structural predictions by validating key residues involved in dimer and tetramer formation but also enabled us to identify PANT variants with differing polymerization propensities, ranging from robust fibril formation to complete loss of polymerization.

To investigate the polymerization mechanism of the PANT domain, we performed in vitro fibril formation assays and measured the hydrodynamic radius with multi-angle dynamic light scattering (MADLS) analysis (Fig. 6, i and j). We tested 9 variants, with mutations covering both dimer- and tetramer-forming interfaces. Because the GST-YFP-tagged PANT domain formed fractal-like fibrils in vitro that closely resembled those observed in protoplasts expressing YFP-HA-PANT, and the YFP-HA-tagged SRFR1 successfully complemented all *srfr1* mutant phenotypes, the N-terminal GST-YFP tag for PANT and its variants were kept in our in vitro fibril formation assays and MADLS measurements. MADLS analysis showed that WT PANT undergoes spontaneous polymerization at room temperature, forming large clusters in PBS solution with hydrodynamic radii in the micrometer range (Fig. 6j), which later aggregated into visible precipitates (Supplementary Figure S5). WT PANT formed fractal linear fibrils in vitro, while PANT^K101A^, a variant affecting the side/bottom tetramer-forming interface (Supplementary Figure S4a), exhibited reduced cluster size and a slower precipitate formation rate (Fig. 6j and Supplementary Figure S5). As expected, PANT^K101A^ failed to form linear fibrils and instead formed aggregation-like structures in vitro (Fig. 6i), further supporting the AlphaFold predictions.

Other mutations, such as R20A, which affects the top/top tetramer formation, and those impacting dimer formation including F47T, and the double mutants L13T/F47T, F47T/V110T, and F47T/E115A, as well as F47T/K101A, which affects both dimer and tetramer interfaces, all exhibited a small hydrodynamic radius in PBS at room temperature (Fig. 6j). These variants almost completely abolished fibril formation with 1% PEG in vitro (Fig. 6i). TPR (Tetratricopeptide Repeat) domains, characterized by 34 amino acid repeats forming a helix-turn-helix structure, are known for mediating protein–protein interactions (Blatch and Lassle 1999). The unique ability of the PANT domain to polymerize and form fibrils unveils a previously uncharacterized property of TPR domains, warranting further study. The fibril-forming property of the PANT domain may cause toxicity in animal cells, potentially explaining the absence of the PANT domain in SRFR1 orthologs across animal species (Supplementary Figure S3a).

### PANT domain polymerization-mediated SRFR1 condensate formation is required for primary root growth

**Figure 7.**
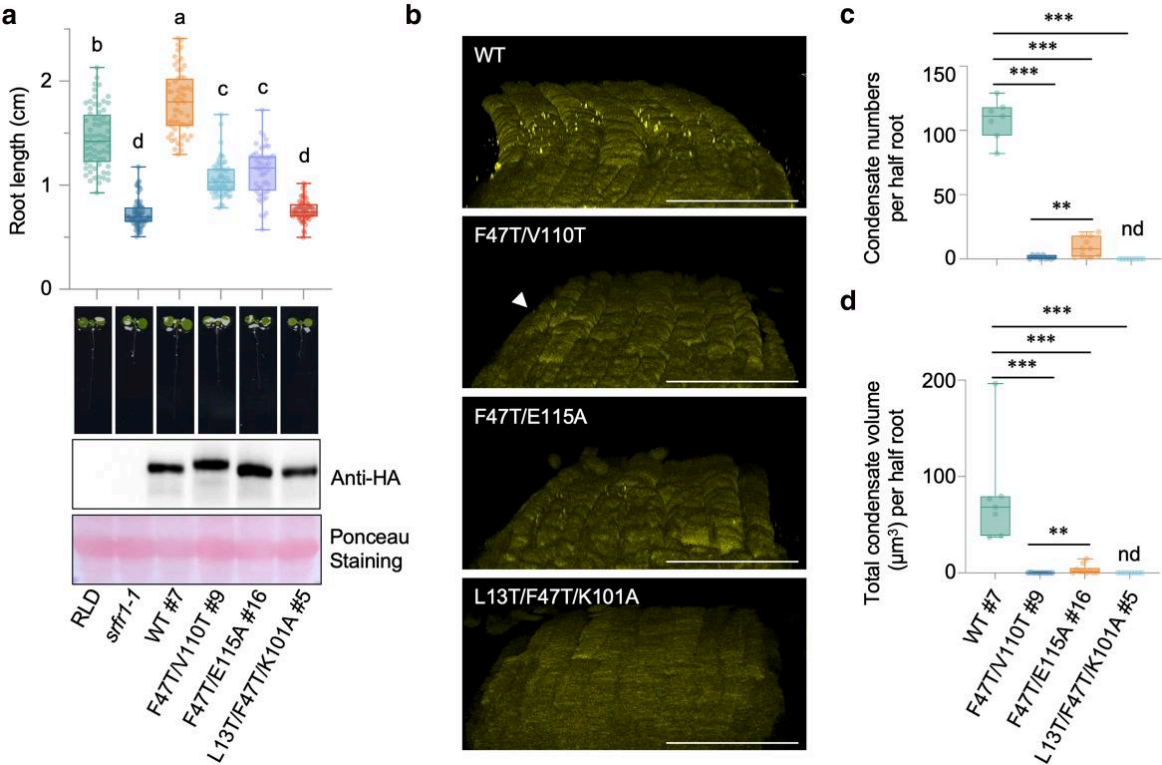
PANT domain-mediated polymerization is required for upper LRC SRFR1 condensate formation and primary root development. a) Primary root length and protein expression of YFP-HA-tagged SRFR1 PANT domain dimer- and tetramer-interface variants. Primary root length was measured with 6-d-old seedlings, *n* = 32 to 51. Letters denote statistically different groups (ordinary one-way ANOVA, Tukey-Kramer grouping). Protein expression levels were detected using an anti-HA antibody. Ponceau S staining of the Rubisco large subunit was used as a protein loading control. b) Condensate accumulation by SRFR1 PANT domain variants in upper LRCs. Confocal 3D images were taken from the roots of 6-d-old seedlings. Seedlings were fixed with 4% PFA for large-scale 3D imaging. Z-stack images were collected by scanning from the root surface to the midsection at 0.42 µm intervals. Bar = 50 µm. The white arrow indicates small condensates in SRFR1^F47T/V110T^ line #9. c and d) Quantification of condensate number and total condensate volume formed by SRFR1 PANT domain variants (*n* = 7 to 10). *** indicates *P* < 0.001 and ** indicates *P* < 0.01 (Unpaired Mann–Whitney test). For a, c, and d), all data points are shown in box-and-whisker plots. Boxes indicate the interquartile range, with center lines denoting the median. Whiskers extend to the minimum and maximum values of the dataset.

To determine the impact of these PANT variants on SRFR1 condensate formation in the upper LRC and primary root growth, we introduced several of these mutations into the native promoter-driven genomic clone *YFP-HA-gSRFR1^WT^* and generated stable transformants with them in the *srfr1-1* mutant background. The SRFR1^L13T/F47^, SRFR1^F47T/V110T^, and SRFR1^F47T/E115A^ variants, which exhibited reduced condensate formation in the upper LRC, partially rescued the short root phenotype of *srfr1-1* (Fig. 7, a to d and Supplementary Figure S6, a and b). Notably, the triple mutant L13T/F47T/K101A variant, which harbors mutations in both the dimer and tetramer interfaces and exhibited only diffuse localization, failed to form condensates in the LRC and completely failed to complement the short root phenotype of *srfr1-1* (Fig. 7, a to d and Supplementary Figure S6b). In addition, although the SRFR1^F47T/E115A^ variant formed significantly more and larger condensates than SRFR1^F47T/V110T^ (Fig. 7, b to d), no significant difference in primary root length was observed (Fig. 7a). The SRFR1^F47T/V110T^ condensate intensity is very low (Supplementary Figure S6a), making it difficult to visualize them after 3D reconstruction due to background signals from epidermal cells (Fig. 7b). These observations suggest that the smaller, fewer, and lower-intensity condensates formed by SRFR1^F47T V110T^ are sufficient for partial complementation. In addition, in protein gels, we observed slower migration of SRFR1^F47T/V110T^ compared with the wild-type and other variants (Fig. 7a). It will be interesting to investigate the molecular basis of this band shift and its potential relationship with condensate formation and root growth in future studies. Collectively, our results support a positive relationship between PANT domain-dependent polymerization-mediated SRFR1 biomolecular condensate formation in the upper LRCs and primary root growth.

### Temperature-dependent regulation of PANT polymerization by IDR1 of SRFR1

**Figure 8.**
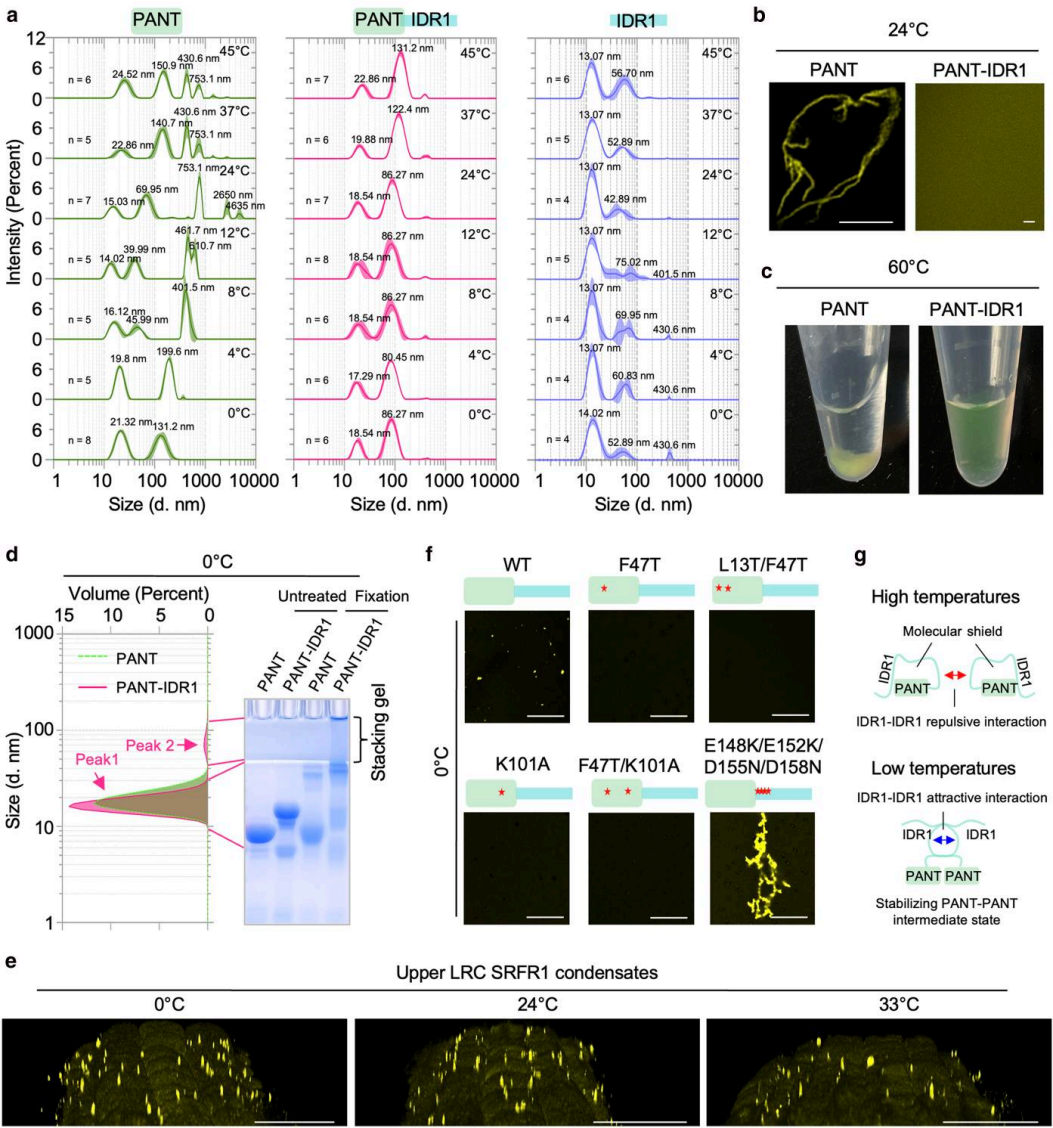
IDR1 inhibits PANT aggregation at high temperatures but promotes PANT polymerization at low temperatures. a) Hydrodynamic radius of GST-eYFP-tagged PANT, PANT-IDR1, and IDR1 variants measured by MADLS at different temperatures using proteins at 30 µM in PBS solution. b) In vitro fibril formation assay of GST-eYFP-tagged PANT and PANT-IDR1 at 30 µM in PBS solution. Samples were incubated at room temperature for 30 min. The laser power to visualize fluorescent protein in the PANT-IDR1 sample was approximately 10-fold higher than with the PANT sample. Bar = 10 µm. c) In vitro sedimentation assay of GST-eYFP-tagged PANT and PANT-IDR1 at 30 µM in PBS solution. Samples were incubated at 60 °C for 15 min. d) In vitro cross-linking assay of GST-eYFP-tagged PANT and PANT-IDR1 at 30 µM in PBS solution. Samples were fixed with 0.0625% glutaraldehyde (v/v) on ice for 10 min. Volume distribution of PANT and PANT-IDR1 was derived from the MADLS experiment from a) at 0 °C. e) Accumulation of SRFR1 condensates in the upper LRC at different temperatures. 3D localization of SRFR1 condensates in the upper LRC. A Z-stack of surface sections in the upper LRC region of 6-d-old seedling roots was taken using confocal microscopy. Bar = 20 µm. f) In vitro condensate formation assay of GST-eYFP-tagged PANT-IDR1 variants at 7.5 µM in PBST buffer containing 0.5% PEG8000 on ice. Bar = 10 µm. g) A proposed model illustrating the chaperone-like function of IDR1 at high and low temperatures.

The above results suggest that the PANT domain and its adjacent IDR1 function together as a condensation module, with PANT polymerization driving the process and IDR1 fine-tuning it. Polymerization processes, such as actin filament assembly, are intrinsically nucleation-limited and autocatalytic. Nucleation must overcome substantial thermodynamic and kinetic barriers, occurring only above a critical concentration or temperature, or with the assistance of nucleation factors (Kashchiev 2015; Pollard 2016; Narayanan et al. 2019; Rodriguez Gama et al. 2021). For example, actin filament assembly requires multiple factors to overcome the energy barrier (Carlsson 2010; Pollard 2016). Once nucleated, polymerization proceeds rapidly and cooperatively, as the initial seed accelerates monomer addition. Because of this intrinsic autocatalytic nature, physiological polymerization must be tightly regulated to prevent pathological aggregates (Narayanan et al. 2019; Rodriguez Gama et al. 2021). Dysregulation can result in irreversible fibril formation, as seen in misfolding-induced amyloid-β (Aβ) aggregation in Alzheimer's disease (Jarrett and Lansbury 1993; Knowles et al. 2014). To further understand how IDR1 regulates PANT polymerization, we conducted in vitro assays using purified PANT, PANT-IDR1, and IDR1. MADLS analysis revealed that the PANT domain is highly sensitive to temperature changes (Fig. 8a). At 0 °C, PANT predominantly existed as monomers, with small fractions forming potential dimers and tetramers (Fig. 8d) corresponding to the hydrodynamic radius peaks at 21.32 nm. As the temperature increased, larger clusters with hydrodynamic radii peaking at 401.5 nm were detected at 8 °C. Further temperature increases led to even larger clusters, reaching micrometer sizes (Fig. 8a). In support of the MADLS measurements, micrometer-sized linear PANT fibrils with fractal branching were readily observed after a 30-min incubation at 24 °C (Fig. 8b). Moreover, visible precipitates rapidly formed by heating to 60 °C for 10 min (Fig. 8c). In contrast, PANT-IDR1 demonstrated high thermal stability, showing no marked changes in hydrodynamic radius between 0 and 24 °C. Importantly, no precipitates formed at 24 °C, even after several days or after being heated to 60 °C for 15 min (Fig. 8, b and c). These findings suggest that IDR1 functions as a molecular shield, protecting the PANT domain from irreversible aggregation at elevated temperatures.

Prevention of aggregation of a folded domain by an adjacent disordered region is consistent with a tuner function and has been observed previously (Lasker et al. 2022). However, unexpectedly, we found that PANT-IDR1 forms high molecular weight clusters at 0 °C, whereas neither PANT nor IDR1 alone exhibits this behavior (Fig. 8, a and d and Supplementary Figure S7a). It is important to note that in dynamic light scattering measurements, the scattered light intensity is proportional to the sixth power of the particle diameter. Consequently, when the intensity distribution is converted to a volume distribution, volume peaks corresponding to larger particles (peak at higher hydrodynamic radius) are much smaller than light scattering peaks might suggest. As shown in Fig. 8d, the second peak (Peak 2) formed by PANT-IDR1 only accounts for about 5% of the total particle volume, while that formed by PANT is negligible.

This observation prompted a detailed analysis of the condensation behavior of PANT, IDR1, and PANT-IDR1 across various temperatures and concentrations (Supplementary Figure S7, b and c). At concentrations of 7.5 µM or below, PANT did not form condensates or fibrils. However, at 15 µM, PANT formed spherical condensates at lower temperatures (0 and 12 °C) and fibrils at 24 and 37 °C. IDR1, on the other hand, remained highly soluble, forming condensates only at 0 °C and at higher concentrations (15 and 30 µM). Interestingly, as a combined module, PANT-IDR1 formed condensates over a broad range of temperatures and concentrations. Indeed, SRFR1 condensates in upper LRCs remained stable across a broad temperature range from 0 to 24 °C, and a substantial number of SRFR1 condensates were still detectable even at 33 °C, a temperature generally considered a high stress condition for Arabidopsis (Fig. 8e) (Vu et al. 2019).

Since PANT alone did not form condensates at low temperatures and low concentrations, we hypothesized that transient interactions involving IDR1 might promote PANT polymerization under these conditions. To test this hypothesis, we introduced mutations into PANT-IDR1. As shown in Fig. 8f, no condensates were detected for the 4 tested PANT-IDR1 variants (F47T, K101A, L13T/F47T, and F47T/K101A), suggesting that PANT polymerization is essential for condensate formation at low temperatures and concentrations. Furthermore, a quadruple mutant of IDR1 (E148K/E152K/D155N/D158N) with enhanced self-interactions dramatically promoted PANT-IDR1 condensate formation (Fig. 8f and Supplementary Figure S7c). In summary, our results suggest that IDR1 plays dual roles in modulating PANT polymerization: it prevents PANT domain aggregation at high temperatures while promoting PANT polymerization at low temperatures (Fig. 8g).

### The zwitterionic nature of IDR1 finetunes condensate formation

**Figure 9.**
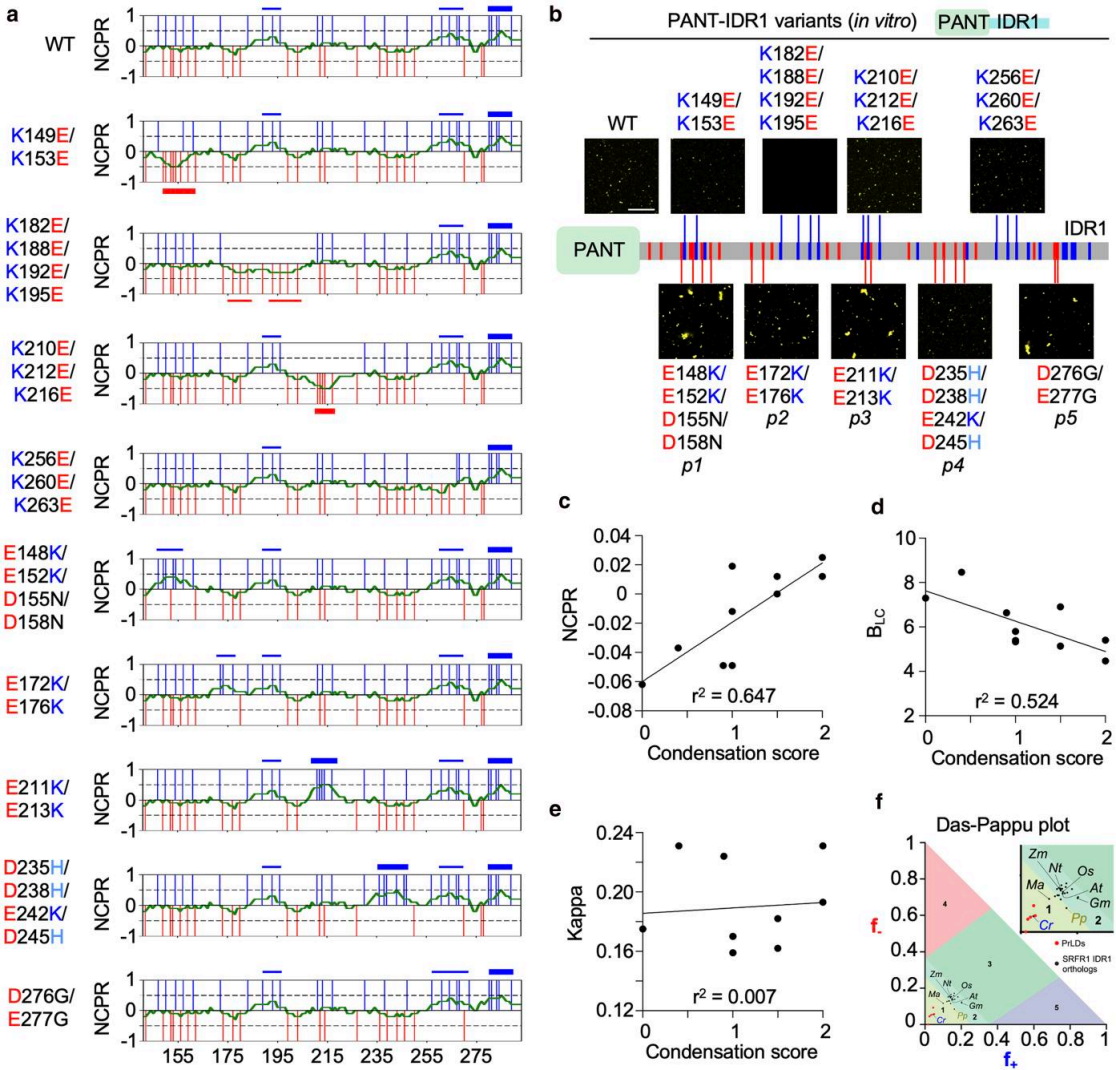
The zwitterionic nature of IDR1 is required for its chaperone-like functions. a) A 10-residue sliding window was used to calculate the local NCPR of IDR1 and its variants (horizontal green traces). Positively and negatively charged residues are indicated by vertical blue and red lines, respectively. Acidic and basic blocks are represented by red (bottom) and blue (top) horizontal bars, respectively. Weak blocks are defined as 0.25 < NCPR < 0.5 or −0.5 < NCPR < −0.25; intermediate blocks as 0.5 ≤ NCPR < 0.75 or −0.75 < NCPR ≤ −0.5; and strong blocks as 0.75 ≤ NCPR ≤ 1 or −1 ≤ NCPR ≤ −0.75. b) In vitro condensate formation assay of GST-eYFP-tagged PANT-IDR1 variants in PBS buffer containing 0.5% PEG8000 (w/v) at 7.5 µM on ice. Bar = 10 µm. c to e) Correlation analysis of the condensation score of PANT-IDR1 variants in b) and the global NCPR, B_LC_, and kappa parameters of the IDR1 variants. The goodness-of-fit (*r*^2^) is determined through linear regression. A condensation score was assigned to each variant, following the method commonly used for scoring disease symptoms in plant immunity (WT = 1, K149E/K153E = 0.4, K182E/K188E/K192E/K195E = 0, K210E/K212E/K216E = 1; K256E/K260E/K263E = 1, E148K/E152K/D155N/D158N = 2, E172K/E176K = 1.5, E211K/E213K = 2, D235H/D238H/E242K/D245H = 1, and D276G/E277G = 1.5). f) The Das-Pappu plot shows the distribution of SRFR1 IDR1 orthologs (light blue dots) and 5 prion-like domains (red dots).

To investigate how IDR1 functions at the molecular level, we performed an extensive mutational analysis targeting IDR1 in the context of the PANT-IDR1 module. Interestingly, we found PANT-IDR1 exhibited completely diffuse localization in rice protoplasts when a 6 aa tail (PGIHLI) was added by the multiple cloning site of the *pSAT6-eYFP* construct. We then took advantage of this localization pattern to explore substitutions that mimic the function of native SRFR1 IDR1. Among the 57 PANT-IDR1 variants tested in rice protoplasts (Supplementary Figure S8), we observed a clear pattern: mutations that changed negatively charged residues to positively charged or polar residues at 6 distinct positions in IDR1 consistently shifted the PANT-IDR1 from diffuse localization to puncta formation. Thus, we reasoned that changing some of the positively charged residues to negatively charged ones in IDR1 would reduce PANT-IDR1 condensate formation (Fig. 9a). Since PANT-IDR1, as well as its positive-to-negative variants, already exhibited diffuse localization in rice protoplasts (Supplementary Figure S9a), we tested this hypothesis using purified proteins. Consistent with the punctate localization observed in rice protoplasts, purified PANT-IDR1 variants (E148K/E152K/D155N/D158N, E172K/E176K, E211K/E231K, and D276G/E277G) showed increased condensate formation in vitro, while the D235H/D238H/E242K/D245H variant did not (Fig. 9b). Conversely, the PANT-IDR1^K149E/K153E^ variant showed reduced condensate formation, and the PANT-IDR1^K182E/K188E/K192E/K195E^ variant completely lost the ability to form condensates. However, no noticeable changes in condensate formation were detected in 2 other PANT-IDR1 variants with positive-to-negative mutations (K210E/K212E/K216E and K256E/K260E/K263E).

We next analyzed the composition and distribution of charged residues in the IDR1 region. The positively and negatively charged residues in SRFR1 IDR1 exhibit an almost perfectly alternating distribution, with only 2 weak basic blocks in the middle and a short intermediate basic block near the C-terminus, as indicated by the local net charge per residue (NCPR) calculated in a 10-residue sliding window (Fig. 9a). Due to this alternating charge pattern, changing negatively charged residues to positively charged residues results in the emergence of basic blocks, and vice versa. Correlation analysis of the condensation score of PANT-IDR1 variants with charge patterning parameters revealed a moderate correlation with the global NCPR (Fig. 9c, *r*^2^ = 0.647) and a moderate negative correlation with the blockiness of like charges (B_LC_) (Fig. 9d, *r*^2^ = 0.524) (Yamazaki et al. 2022). In contrast, no correlation was found with the kappa parameter (Fig. 9e, *r*^2^ < 0.01), which describes charge asymmetry (Das and Pappu 2013). Taken together, these results suggest that the zwitterionic nature of SRFR1 IDR1 is required for its chaperone-like function.

We then analyzed the composition and distribution of charged residues in SRFR1 IDR1 orthologs. Despite low sequence similarity, IDR1 from SRFR1 orthologs in angiosperms is moderately charged, with an average fraction of charged residues (FCR) of 0.29, which is classified as a weak polyampholyte (Das and Pappu 2013). Most of them fall in region 2 of the Das-Pappu plot, exhibiting a nearly equal ratio (∼0.94) of positively to negatively charged residues (P/N ratio) (Fig. 9f and Supplementary Figure S9b) (Das and Pappu 2013). We also performed substitution analysis using prion-like domains (PrLDs) and SRFR1 IDR1 orthologs. As expected, all 4 tested PrLDs failed to mimic the native IDR1 of SRFR1 in maintaining diffuse localization (Supplementary Figure S10, a and b). SRFR1 IDR1 orthologs from soybean (*Glycine max*) and rice (*Oryza sativa*) functionally mimicked the native Arabidopsis IDR1 (Supplementary Figure S10, a and b). Correspondingly, rice SRFR1 IDR1, which is highly similar to Arabidopsis SRFR1 IDR1, fully substituted for Arabidopsis IDR1 in terms of LRC condensate accumulation and primary root growth (Supplementary Figure S10c). SRFR1 IDR1 orthologs from maize (*Zea mays*) and especially tobacco (*Nicotiana tabacum*) failed to mimic the native Arabidopsis IDR1, likely due to the presence of strong basic blocks (Supplementary Figure S10a). Notably, the NtSRFR1 IDR1 contains 4 basic blocks and 1 weak acidic block, with an intermediate basic block in the middle resembling the E211K/E213K variant of AtSRFR1 IDR1 (Fig. 9b and Supplementary Figure S10a). To test whether the punctate localization of PANT-IDR1^NtSRFR1^ is due to the enrichment of basic blocks, we generated 2 variants (IDR1^NtSRFR1 K194E/K195E^ and IDR1^NtSRFR1 K197E/K198E^) to weaken intermediate basic block #2, and 1 variant (IDR1^NtSRFR1 K219E/K220E^) to eliminate weak basic block #3. All 3 variants, on average, exhibited reduced condensate formation in rice protoplasts (Supplementary Figure S10d). These results further support the notion that the zwitterionic nature of SRFR1 IDR1 is important for its chaperone-like function.

### The zwitterionic IDR1 of SRFR1 can be functionally substituted by dehydrins

**Figure 10.**
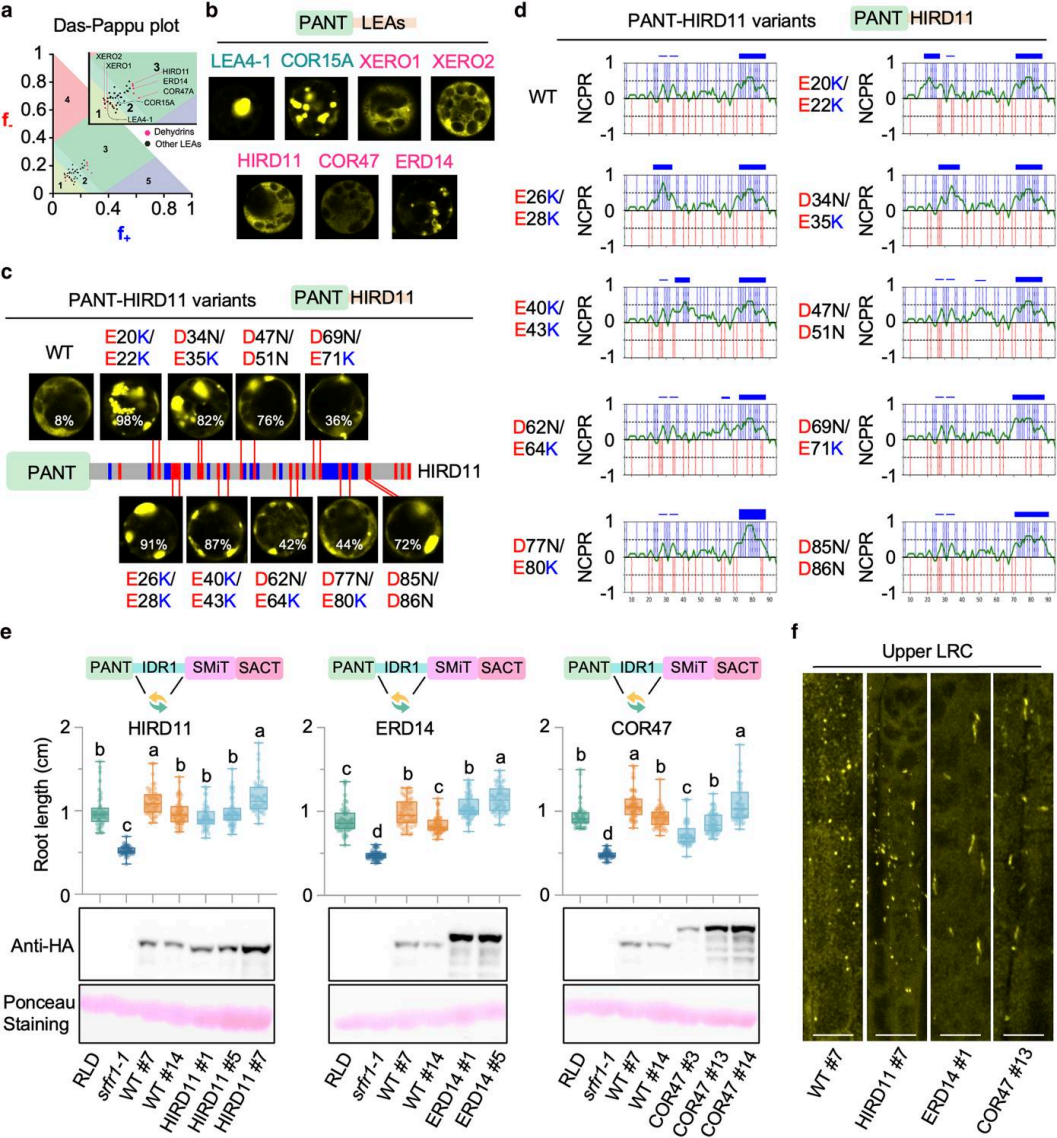
The zwitterionic IDR1 of SRFR1 can be functionally substituted by zwitterionic dehydrins. a) The Das-Pappu plot shows the distribution of Arabidopsis LEA proteins. b and c) Subcellular localization of PANT-LEAs and variants of PANT-HIRD11 in rice protoplasts. Images were taken using the same confocal microscopy settings. A diagram of the arrangement of residues in HIRD11 is shown. Positively and negatively charged residues are colored in blue and red, respectively. Other residues are shown in gray. The average percentage of protoplasts exhibiting puncta formation is indicated on each representative image. The percentage was determined by counting 3 to 5 randomly selected low-magnification images from a large area, each containing 80 to 120 protoplasts. d) The local NCPR of HIRD11 and its variants (horizontal green traces). Positively and negatively charged residues are indicated by vertical blue and red lines, respectively. Basic blocks are represented by blue horizontal bars (top). Weak blocks are defined as 0.25 < NCPR < 0.5; intermediate blocks as 0.5 ≤ NCPR < 0.75; and strong blocks as 0.75 ≤ NCPR ≤ 1. e) Primary root length of YFP-HA-tagged SRFR1 with HIRD11, ERD14, or COR47 IRD1 substitutions. Primary root length was measured with 6-d-old seedlings, *n* = 42 to 65. All data points are shown in box-and-whisker plots. Boxes indicate the interquartile range, with center lines denoting the median. Whiskers extend to the minimum and maximum values of the dataset. Letters denote statistically different groups (ordinary one-way ANOVA, Tukey-Kramer grouping). Protein expression levels were detected using an anti-HA antibody. Ponceau S staining of the Rubisco large subunit was used as a protein loading control. f) Upper LRC condensates of YFP-HA-tagged SRFR1 with HIRD11, ERD14 or COR47 IDR1 substitutions. Images were taken with roots of 6-d-old seedlings. Multiple single sections were taken using the same confocal microscopy settings; maximal projection images are shown, bar = 10 µm.

Dehydrins and other groups of LEA proteins are highly enriched in charged residues (Hernandez-Sanchez et al. 2022). For instance, more than 40% of the residues in HIRD11, ERD14, and COR47, 3 extensively studied dehydrins, are charged, classifying them as strong polyampholytes that fall into region 3 of the Das-Pappu plot (Fig. 10a) (Das and Pappu 2013). To assess whether these highly charged LEA proteins could functionally mimic SRFR1 IDR1 in preventing PANT aggregation, we fused PANT with 5 different dehydrins. As shown in Fig. 10b, 4 of the 5 dehydrins (COR47 > HIRD11 > XERO1 > XERO2) resulted in predominantly diffuse localization, similar to what was observed with the native IDR1. Interestingly, when fused with ERD14, PANT formed large vesicle-like structures, some of which were as large as chloroplasts. However, fusion with COR15A or LEA4-1, which belong to type III and type IV LEA proteins, failed to prevent PANT aggregation (Fig. 10b).

Given the similar localization pattern of PANT-HIRD11 in protoplasts, as well as the similar charge distribution pattern of HIRD11 and SRFR1 IDR1 (Fig. 10b), we tested whether the zwitterionic nature of HIRD11 plays a role in regulating PANT polymerization. As shown in Fig. 10c, converting negatively charged residues to positively charged or polar ones at 9 distinct positions of HIRD11 consistently shifted the diffuse localization toward puncta formation, phenocopying the negative-to-positive mutations in native SRFR1 IDR1. Due to the nearly perfect alternating charge distribution in the N-terminal region of HIRD11 (1 to 70aa), these mutations introduced new basic blocks or enhanced the existing basic blocks (Fig. 10d). Our results suggest that, in addition to its well-characterized short linear motifs (SLiMs), such as the N1 segment and the K-segment (Yokoyama et al. 2020), the zwitterionic nature of HIRD11 may also play an important role in its function and warrants further investigation.

Next, we substituted the native SRFR1 IDR1 with HIRD11, ERD14, and COR47 in the genomic clone *YFP-HA-gSRFR1^WT^*, generating *YFP-HA-gSRFR1^IDR1-HIRD11^*, *YFP-HA-gSRFR1^IDR1-ERD14^*, and *YFP-HA-gSRFR1^IDR1-COR47^*, respectively. All these dehydrin substitutions rescued the short root phenotype of *srfr1-1* (Fig. 10e). Strikingly, SRFR1^IDR1-ERD14^ even produced significantly longer roots (Fig. 10e), possibly due to higher protein accumulation. Consistent with the PANT-IDR1 deletion lines (Fig. 5, d and f), high-expression lines were readily obtained for all 3 dehydrin substitutions (Fig. 10e), further supporting a role for the native IDR1 in maintaining SRFR1 protein homeostasis. We also examined the formation of biomolecular condensates by the dehydrin substitution constructs in LRCs (Fig. 10f). While SRFR1^IDR1-HIRD11^ formed condensates morphologically similar to WT, SRFR1^IDR1-ERD14^ and SRFR1^IDR1-COR47^ formed visibly larger condensates. It is worth noting that the wild-type accumulation level of SRFR1^IDR1-COR47^ only partially rescued the *srfr1-1* phenotype, possibly due to the enrichment of acidic blocks in COR47. Altogether, our PANT-LEA fusion experiments in protoplasts and stable transgenic plants reinforced the conclusion that the general zwitterionic nature of IDR/IDPs is necessary for their chaperone-like functions.

### Promoting primary root growth by shifting the zwitterionic nature of IDR1

**Figure 11.**
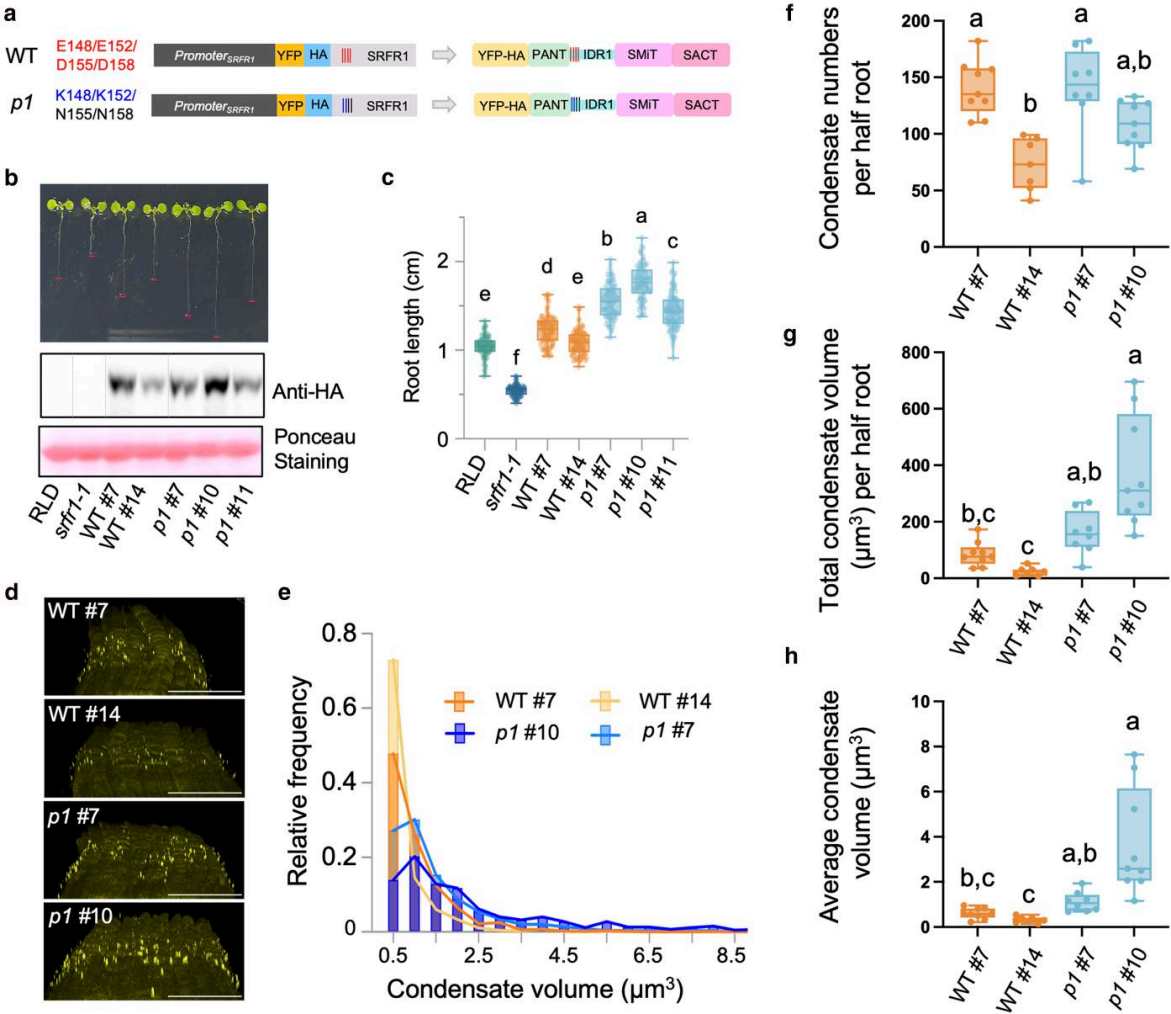
Shifting the zwitterionic IDR1 of SRFR1 to a more positively charged state enhances upper LRC SRFR1 condensate accumulation and improves primary root growth. a) Diagram of YFP-HA-tagged SRFR1^WT^ and SRFR1*^p1^* variant constructs used for transformation of *srfr1-1*. b and c) Primary root length and protein expression of the SRFR1*^p1^* variant under constant temperature. Primary root length is measured with 6-d-old seedlings, *n* = 58 to 93. Letters denote statistically different groups (ordinary one-way ANOVA, Tukey-Kramer grouping). Protein expression levels were detected using an anti-HA antibody. Ponceau S staining of the Rubisco large subunit was used as a protein loading control. d) Condensate accumulation in upper LRCs formed by wild-type SRFR1 and SRFR1 IDR1 domain variant *p1*. Confocal 3D images were taken from the roots of 6-d-old seedlings. Seedlings were fixed with 4% PFA for large-scale 3D imaging. Z-stack images were collected by scanning from the root surface to the midsection at 0.42 µm intervals. Bar = 50 µm. e) Relative frequency distribution of condensate volumes formed by SRFR1^WT^ and SRFR1*^p1^*. f to h) Quantification of condensate number, total condensate volume, and average condensate volume formed by SRFR1^WT^ and SRFR1*^p1^* (*n* = 7 to 9). Letters denote statistically different groups (one-way ANOVA, Kruskal–Wallis Dunn's post-hoc test). For c and f to h), all data points are shown in box-and-whisker plots. Boxes indicate the interquartile range, with center lines denoting the median. Whiskers extend to the minimum and maximum values of the dataset.

We observed that increased formation of LRC SRFR1 condensates correlated with improved primary root growth (Fig. 3, c and d). The enhanced condensate formation observed in vitro with the negative-to-positive PANT-IDR1 variants prompted us to explore whether further improving root growth could be achieved by shifting the zwitterionic nature of IDR1 toward a more positively charged state. For simplicity, we designated these negative-to-positive mutations in IDR1 as “p variants” (Fig. 9b). We then introduced the p1 variant mutations (E148K/E152K/D155N/D158N) into the YFP-HA-tagged genomic SRFR1 construct and transformed *srfr1-1* with it (Fig. 11a). As expected, SRFR1^p1^ variant lines exhibited longer primary root growth than SRFR1^WT^ lines (Fig. 11, b and c).

SRFR1^p1^ formed significantly larger condensates than SRFR1^WT^ (Fig. 11, d, e, g, and h), consistent with the larger condensate observed with PANT-IDR1^p1^ in vitro (Fig. 9b). While most SRFR1WT condensates ranged from 0 to 1 µm^3^, with larger ones rarely found, larger condensates (1 to 3 µm^3^) were frequently observed in SRFR1^p1^ lines (Fig. 11e). Due to lower protein expression, SRFR1^WT^ line #14 exhibited significantly fewer condensates and trended toward lower total and average condensate volumes compared with SRFR1^WT^ line #7 (Fig. 11, d to h). Although SRFR1^p1^ line #10 contained, on average, slightly fewer condensates than SRFR1^WT^ line #7, its total condensate volume was significantly higher by ∼4.35-fold, driven by a significant ∼6-fold increase in average condensate volume. Similarly, SRFR1^p1^ line #7 trended toward higher total and average condensate volumes than SRFR1^WT^ line #7, despite lower protein abundance. Together, these results suggest that LRC condensate accumulation is influenced by both protein concentration and PANT-IDR1 properties, with a shift toward a more positively charged IDR1 enhancing SRFR1 condensate formation and further promoting root growth.

### Identification of putative components of SRFR1 condensates in upper LRCs

**Figure 12.**
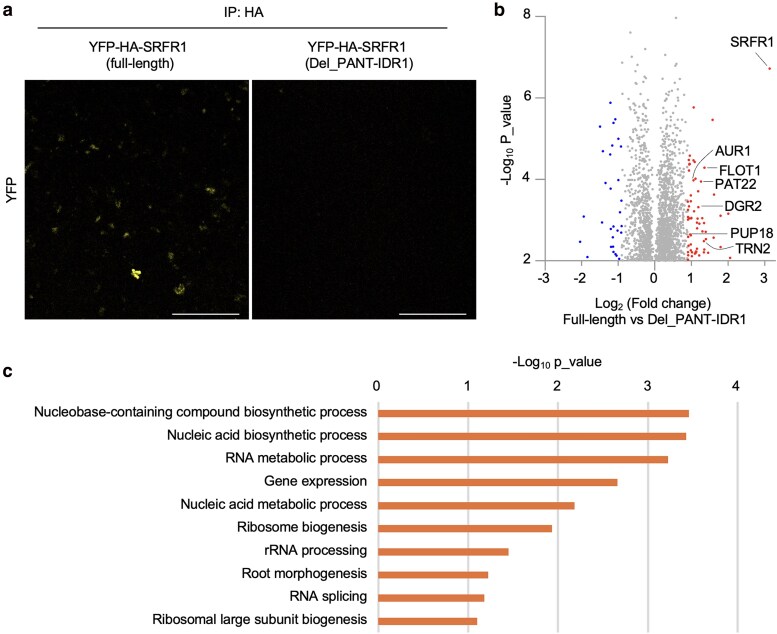
Identification of putative components of SRFR1 condensates in upper LRCs. a) SRFR1 condensates were purified with anti-HA magnetic beads from ∼2,000 roots of vertically grown YFP-HA-tagged full-length SRFR1 #7 seedlings. The condensate-defective Del_PANT-IDR1 line was used as a control. Bar = 20 µm. b) Volcano plot showing differentially enriched proteins based on 3 replicates of IP-MS data comparing full-length and Del_PANT-IDR1 SRFR1 transgenic lines (cutoff: ± 0.9 log_2_ fold changes). Red dots indicate proteins enriched in YFP-HA-tagged full-length SRFR1 samples, while blue dots indicate proteins enriched in control samples (Del_PANT-IDR1). c) Gene ontology (GO) analysis of 63 putative SRFR1 condensate components. The −log_10_  *P*-values of enriched biological process GO terms are shown.

To identify putative components of SRFR1 condensates in the upper LRCs, we performed immunoprecipitation-mass spectrometry (IP-MS) by enriching SRFR1 condensates from root tips of approximately 6,000 vertically grown seedlings. Given similar protein expression levels, we used SRFR1^WT^ line #7 with the condensate-defective SRFR1^Del_PANT-IDR1^ #5 truncation variant as a negative control. Since PANT-PANT interactions are primarily driven by hydrophobic interactions (Fig. 6, c and h), we found that Direct-23, a dye with phenyl groups, could dissolve SRFR1 condensates even after paraformaldehyde (PFA) fixation during our optimization of condensate quantification protocols. Thus, we conducted our immunoprecipitation using only PBS buffer with protease inhibitors and washed with PBS containing 0.05% Tween-20 to maintain condensate integrity (Fig. 12a).

IP-MS analysis identified 63 proteins significantly enriched in full-length SRFR1 samples (Fig. 12b and Supplementary Data Set 1). According to the Root Cell Atlas database (https://rootcellatlas.org), most identified proteins were expressed in LRCs (Supplementary Figure S12). Among those enriched proteins, several of them have been reported to be involved in root development, including TORNADO2 (TRN2), FLOTILLIN1 (FLOT1), AURORA KINASE1 (AUR1), PROTEIN S-ACYL TRANSFERASE22 (PAT22), and DUF642 L-GalL RESPONSIVE GENE2 (DGR2) (Van Damme et al. 2011; Gao et al. 2012; Li et al. 2012b; Liu et al. 2024). TRN2, a tetraspanin required for radial patterning of root tissues, suppresses LRC cell fate in epidermal cells (Cnops et al. 2000), although its specific role in LRCs remains poorly understood (Fendrych et al. 2014). FLOT1, a membrane microdomain-associated protein involved in clathrin-independent endocytosis, is essential for the regulation of meristem size (Li et al. 2012b). As a purine permease, PURINE UPTAKE PERMEASE (PUP18) may regulate cytokinin concentrations in root cell layers. Mutants in many of the genes encoding these proteins display compromised primary root growth (Zürcher et al. 2016). Gene Ontology (GO) enrichment analysis suggests that SRFR1 condensates may also contribute to RNA processing and epigenetic regulation (Fig. 12c), potentially acting upstream to fine-tune ethylene signaling. Collectively, these findings provide a foundation for understanding the molecular composition and regulatory roles of SRFR1 condensates in LRCs, warranting further investigation.

## Discussion

### Upper LRC-specific SRFR1 condensates and root growth

Previous studies have shown that LRCs function as an auxin sink to control meristem size, while developmentally regulated PCD of LRCs releases auxin to promote lateral root initiation above the meristem (Xuan et al. 2016; Di Mambro et al. 2019). This dual role raises a question of how the auxin minimum in the TZ is maintained despite oscillations in rhizosphere auxin levels caused by auxin release by dying LRCs. We observed that SRFR1 condensates are present only in live upper LRCs, and that new upper LRCs containing SRFR1 condensates often cover the epidermis when neighboring LRCs are undergoing PCD (Fig. 3c). These observations suggest that live upper LRCs with SRFR1 condensates act as a barrier, buffering the TZ against rhizosphere auxin oscillations.

In addition, several putative SRFR1 condensate components (eg TRN2, FLOT1, and PUP18) are associated with plasma membrane functions. These proteins may serve as nucleation sites for SRFR1 condensate assembly or have their activities modulated through sequestration within condensates. Among them, *PUP18*, encoding a highly expressed putative LRC purine uptake permease (Supplementary Figure S12), likely regulates cytokinin uptake into LRCs. Although its function remains uncharacterized, ethanol-inducible silencing of its homolog *PUP14* caused a severely shortened root phenotype (Zürcher et al. 2016). Thus, it is tempting to hypothesize that sequestration of PUP18 into SRFR1 condensates may increase apoplastic cytokinin levels, suppress the expression of *PIN* genes (*PIN1*, *PIN3*, and *PIN4*), and thereby reduce auxin accumulation in the TZ. The specific localization of SRFR1 condensates within the upper LRCs underscores the need for spatially resolved analyses to define the distinct regulatory contributions of the upper, middle, and bottom LRC regions in meristem maintenance.

### Thermally stable and stimuli-responsive upper LRC SRFR1 condensates fine-tune root growth

**Figure 13.**
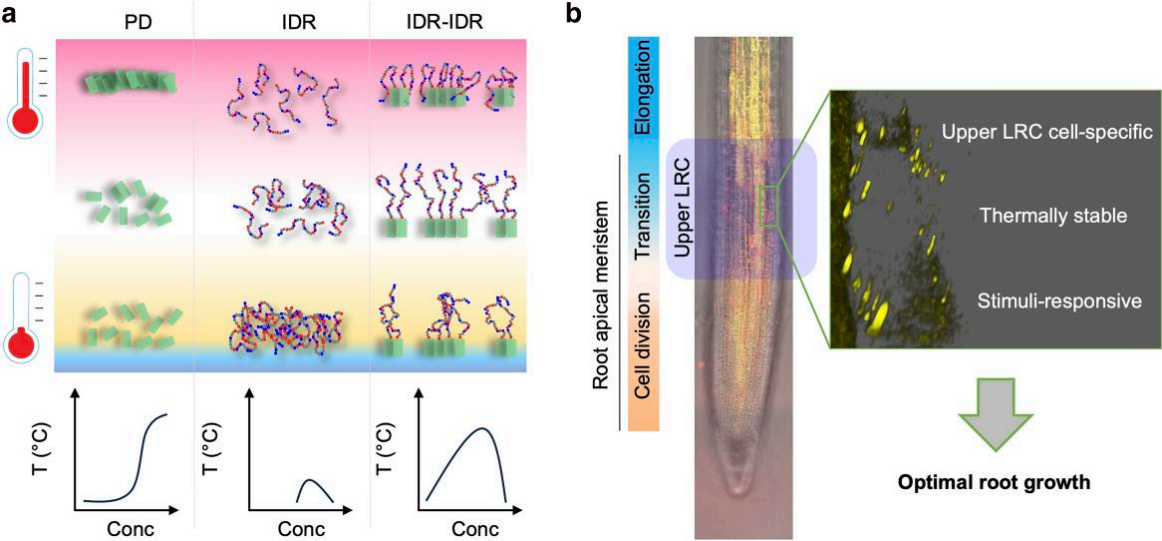
A proposed model illustrating the biological significance of COMET, enabling condensate formation across a broad range of temperatures. a) COMET mechanism at the molecular level. b) The upper LRC-specific SRFR1 condensates are stable in a broad temperature range but are sensitive to both developmental and environmental cues. For illustration purposes, the merged root image was reused from Fig. 3b.

Unlike animals, plants cannot relocate to escape daily or seasonal temperature fluctuations. Although major progress has been made in identifying thermosensors and elucidating thermosensation (Jung et al. 2023), the mechanisms by which plants buffer thermal fluctuations remain largely unknown. In this study, we discovered a mechanism that we call COMET (Combined Module Equilibrating Temperature) (Fig. 13a). COMET depends on a thermally stable condensation module that can be generated by the combination of a thermosensitive PD and a zwitterionic IDR in which the dynamic changes in PD-PD, PD-IDR, and IDR-IDR interactions in response to temperature fluctuations contribute to the thermal stability of the PD-IDR module. Since hydrogen bonding and electrostatic interactions are favored at lower temperatures, most zwitterionic polymers exhibit UCST behavior (Das and Pappu 2013; Blackman et al. 2019; Bianchi et al. 2020; Huihui and Ghosh 2020; Devarajan et al. 2022). The entropy-driven polymerization of PDs is reminiscent of the phase separation of LCST-type polymers. Thus, the PANT-IDR1 module can be considered as an ensemble of LCST and UCST block copolymers. We propose that the COMET mechanism derived from the PANT-IDR1 module not only provides a principle for designing polymerization-mediated synthetic biomolecular condensates but also may inspire the design of temperature-invariant materials for industrial and biomedical applications.

At low temperatures, the intermolecular interactions of the zwitterionic IDR, stabilized by hydrogen bonding and electrostatic forces, promote PD polymerization, possibly by bringing PDs into closer proximity and stabilizing intermediate states of PD–PD interactions through transient and labile IDR–IDR interactions (Fig. 13a). In biotechnological applications, the covalent addition of zwitterionic polymers (eg poly-carboxylbetaine) or zwitterionic polypeptides (eg poly-EK) has been employed to stabilize therapeutic proteins or commercialized enzymes (Keefe and Jiang 2011; Xiong et al. 2011; Liu et al. 2015; Morimoto and Yamamoto 2021).

At high temperatures, the transient and labile interactions between IDR and PD attenuate PD polymerization, preventing irreversible aggregation (Fig. 13a). In this context, the IDR acts like a molecular shield. At intermediate temperatures, intermolecular hydrogen bonding and electrostatic interactions of the zwitterionic IDR are disrupted due to increased molecular motion, while the PD–PD interaction is enhanced as temperature rises (Fig. 13a). Indeed, upper LRC SRFR1 condensates are stable over a wide range of temperatures (Fig. 8e).

In principle, to ensure optimal growth, some development-related condensates should remain stable under daily temperature fluctuations. A key role for the LRC in controlling meristem size has been established by several studies, and the root cap not only protects the meristem from mechanical damage and pathogens, but also is the site for perception and integration of diverse external and internal stimuli (Hahn et al. 2008; Kanno et al. 2016; Di Mambro et al. 2019; Ganesh et al. 2022). The accumulation and disassembly of SRFR1 condensates in upper LRCs are dynamic and highly sensitive to both hormonal treatments and changes in growth conditions (Figs. 3 and 4). Thus, we propose that the upper LRC-specific, thermally stable but stimuli-sensitive SRFR1 condensates are important for the promotion of optimal growth, especially during early spring when daily temperature fluctuations are large (Fig. 13b).

### Association with zwitterionic IDRs is common for polymerization domains

In general, polymerization is an entropy-driven process. As temperature increases, substantial entropy gains result from the elimination of semi-ordered water molecules (water cages) enveloping monomers during polymerization (Blackman et al. 2019; Yu et al. 2021; Baddam and Tenhu 2023; Nan et al. 2023). From a thermodynamic viewpoint, PDs tend to form irreversible aggregates as the temperature increases, driven by the system's entropy maximization. Protein polymerization plays fundamental roles in forming both structural and signaling higher-order assemblies, a process that is widespread across all kingdoms of life (Wu 2013; Wu and Fuxreiter 2016). A critical, yet largely overlooked and unexplored, question is how irreversible aggregation of PDs is prevented at physiologically relevant temperatures.

In this study, we found that the irreversible aggregation of the PANT PD in SRFR1 is prevented by its adjacent zwitterionic IDR1 or fusion with zwitterionic dehydrins (Figs. 8, a to c and 10, a to d). Interestingly, the association of a PD with a zwitterionic IDR is also observed in other PDs, such as the sterile alpha motif (SAM) domain (Knight et al. 2011; Denay et al. 2017; Ray and Hewitt 2023) and the Disheveled/Axin (DIX) domain (Supplementary Figure S11, a and b) (Schwarz-Romond et al. 2007; Bienz 2020; van Dop et al. 2020). Most of the IDRs adjacent to SAM and DIX domains fall into region 2 of the Das-Pappu plot (Supplementary Figure S11, c and d), similar to the SRFR1 IDR1 orthologs found in angiosperm plants (Fig. 9f). This suggests that a similar COMET mechanism may apply to SAM- or DIX-containing proteins, preventing irreversible aggregation and promoting physiological polymerization at different temperatures and under temperature fluctuations. Moreover, this COMET mechanism might also be generalized to oligomerization domains. This notion is supported by observations that the IDRs of Crn1 and PopZ are also zwitterionic and play a role in fine-tuning the oligomerization of their adjacent coiled-coil domains (Lasker et al. 2022; Han et al. 2023). It would be interesting to test whether the zwitterionic IDRs of Crn1 and PopZ1 play a similar role in promoting oligomerization at low temperatures and preventing aggregation at high temperatures.

In this study, we mainly focused on the condensation mechanism of the PANT-IDR1 module of SRFR1, which has guided the engineering of this module to enhance plant growth under both normal conditions and temperature fluctuations. However, the function of the SACT domain of SRFR1 remains unknown. Interestingly, the predicted SACT domain shares structural similarities with the recently resolved bacterial effector AvrB, which catalyzes the rhamnosylation of its plant virulence target RIN4 using UDP-rhamnose as a substrate (Peng et al. 2024). Although endogenous protein rhamnosylation has not been reported in plants, it is tempting to purify the SACT domain alongside putative SRFR1 condensate components to test whether the SACT domain could rhamnosylate its targets in vitro. In the future, validating the compositions of LRC SRFR1 condensates and characterization of the SACT domain's biochemical function will further facilitate engineering of SRFR1 to improve plant growth under fluctuating environments.

## Materials and methods

### Plant growth

Arabidopsis (*Arabidopsis thaliana*) ecotype accessions Col-0, RLD, mutants *srfr1-1*, *srfr1-2*, *srfr1-4*, *snc1-11*, *eds1-2*, *srfr1-4 snc1-11*, and *srfr1-4 eds1-2* were described previously (Kwon et al. 2009; Kim et al. 2010; Bhattacharjee et al. 2011; Garner et al. 2021). *etr1-3* (CS3070), *ein2* (CS3071), and *pCyclinB1::GUS* (CS68143) were obtained from Arabidopsis Biological Research Center (ABRC). SRFR1 CRISPR mutants in Col-0 (*srfr1^Col-0^*), RLD (*srfr1^RLD^*), and Ws (*srfr1^Ws^*) backgrounds were generated in this study; desired mutations were confirmed by Sanger sequencing. *srfr1-4 etr1-3* and *srfr1-4 ein2* were generated by standard genetic crossing; double mutants were characterized by PCR-based genotyping and confirmed by Sanger sequencing. eYFP-HA-gSRFR1^WT^; *srfr1-1*, eYFP-HA-gSRFR1^Del_PANT^; *srfr1-1*, eYFP-HA-gSRFR1^Del_PANT-IDR1^; *srfr1-1*, eYFP-HA-gSRFR1^Del_IDR2^; *srfr1-1*, eYFP-HA-gSRFR1^WT^; *srfr1-4*, eYFP-HA-gSRFR1^Del_PANT^; *srfr1-4*, eYFP-HA-gSRFR1^Del_PANT-IDR1^; *srfr1-4*, eYFP-HA-gSRFR1^Del_IDR2^; *srfr1-4*, eYFP-HA-gSRFR1^L13T/F47T/K101A^; *srfr1-1*, eYFP-HA-gSRFR1^F47T/E115A^; *srfr1-1*, eYFP-HA-gSRFR1^F47T/V110T^; *srfr1-1*, eYFP-HA-gSRFR1^L13T/F47T^; YFP-HA-gSRFR1^OsIDR1 sub^; *srfr1-1*, eYFP-HA-gSRFR1^IDR1-HIRD11^; *srfr1-1*, eYFP-HA-gSRFR1^IDR1-ERD14^; *srfr1-1*, eYFP-HA-gSRFR1^IDR1-COR47^; *srfr1-1*, and eYFP-HA-gSRFR1^*p1* (E148K/E152K/D155N/D158N)^; *srfr1-1* were generated in this study (see Supplementary Data Set 2 for more details).

For root length measurement, if not specified, Arabidopsis seeds were germinated and grown on half-strength Murashige and Skoog (1/2 MS) medium containing 1% (w/v) sucrose, 0.05% (w/v) 2-(N-morpholino) ethanesulfonic acid (MES), and 0.5% agar (pH = 5.7) in transparent Petri dishes placed horizontally on a rack under constant light (75 µE m^−2^ s^−1^) at 23 °C. For root length measurement under temperature fluctuations, seeds were sown in Petri dishes as above, which were then floated on water baths with temperatures alternating every 12 h. For the vertical growth assay, Arabidopsis seeds were germinated and grown on 1/2 MS medium containing 1% (w/v) sucrose, 0.05% MES, and 1.2% agar (pH = 5.7). For liquid growth assays, Arabidopsis seeds were germinated and floated on 1/2 MS medium containing 0.25% (w/v) sucrose and 0.05% (w/v) MES (pH = 5.7). Seedlings from day 6 to day 8 were used for root length measurement and confocal imaging. For the RT-qPCR experiment, seedlings grown in liquid medium were transferred to fresh liquid medium at day 6, and root and shoot tissues were harvested at day 12. For transgenic plant screening, Arabidopsis seeds were germinated and grown on half-strength 1/2 MS medium plates containing 1% (w/v) sucrose, 0.05% (w/v) 2-(N-morpholino) ethanesulfonic acid (MES), 0.5% agar (pH = 5.7), 25 µg/mL kanamycin, and 100 µg/mL timentin. For seed propagation, all Arabidopsis plants were grown in soil (Beger BM2 seed germination mix) in a Conviron growth chamber at 23 °C, 50% relative humidity, and 120 µE m^−2^ s^−1^ light with a 16 h/8 h light/dark photoperiod. For protoplast preparation, seeds of Arabidopsis were directly sown on soil and grown in a Conviron reach-in growth chamber at 22 °C, 75% relative humidity, and 100 µE m^−2^ s^−1^ light with a 12 h/12 h light/dark photoperiod. Rice seedlings were grown on 1/2 MS medium containing 1% (w/v) sucrose and 0.8% agar in a transparent plastic cup under constant light (200 µE m^−2^ s^−1^) at 30 °C. Seven to ten-day-old seedlings were used for protoplast isolation.

### Plasmid construction and transgenic plant preparation

The *SRFR1* CRISPR construct was generated according to our previous work (Zheng et al. 2020). Briefly, 2 guide RNAs targeting *SRFR1* exon 3 and exon 6 and driven by Arabidopsis U6-26 and U3b promoters, were cloned into the *pAtEC1.2e1p::Cas9-GFP-gRNA* vector. *SRFR1* CRISPR mutants in Col-0, RLD, Ws, and *pCyclinB1::GUS* (CS68143) background were generated by *Agrobacterium tumefaciens* GV3101-mediated transformation. Positive transformants were selected by spraying Basta and confirmed by Sanger sequencing. Eventually, Cas9- and gRNA-free lines with the same 1 bp insertion allele in all backgrounds were used for further analysis.

For generating eYFP-HA-tagged SRFR1 transgenic lines, a previously reported HA-tagged 9 kb SRFR1 genomic fragment in *pCambia2300-HA-gSRFR1* was modified by overlap PCR to introduce AatII and SpeI cloning sites; then the eYFP tag was inserted between these 2 sites just before the HA tag. This construct was designated as *pCambia2300-eYFP-HA-gSRFR1^WT^*. Subsequently, SRFR1 domain truncation binary constructs were generated by overlap PCR. To make the PANT domain truncation construct (SRFR1^Del_6–134^), a ∼930 bp fragment 1 was PCR amplified with PANTdel-LP + PANTdel-Overlap-RP. A 756 bp fragment 2 was obtained with PANTdel-Overlap-RP + PANTdel-RP. Using fragments 1 and 2 as templates, PCR with PANTdel-LP + PANTdel-RP was used to amplify a ∼1,660 bp fragment. This band was cut with AatII and XbaI to recover a ∼1,200 bp band and ligate it to AatII- and XbaI-cut *pCambia2300-eYFP-HA-gSRFR1^WT^*. Then we used PANTdel-RP to sequence the construct. Correspondingly, the PANT-IDR1 deletion (SRFR1^Del_6–289^) construct was generated with primer combinations of PANTdel-LP + PANT-IDR1del-Overlap-RP, PANT-IDR1del-Overlap-LP + PANT-IDR1del-RP, and PANTdel-LP + PANT-IDR1del-RP. The resulting fragment was cut with AatII and XbaI. A ∼300 bp band was recovered and ligated to AatII- and XbaI-cut *pCambia2300-eYFP-HA-gSRFR1^WT^*. IDR2 deletion (SRFR1^Del_715–751^) was generated with primer combinations of IDR2del-LP + IDR2del-Overlap-RP, IDR2del-Overlap-LP + IDR2del-RP, and IDR2del-LP + IDR2del-RP. The resulting fragment was digested with KfII and BstBI; a ∼1,800 bp band was recovered and ligated to KflI- and BstBI-cut *pCambia2300-eYFP-HA-gSRFR1^WT^*. To generate *pCambia2300-eYFP-HA-gSRFR1^L13T/F47/K101A^*, *pCambia2300-eYFP-HA-gSRFR1^L13T/F47T^*, *pCambia2300-eYFP-HA-gSRFR1^F47T/V110T^*, *pCambia2300-eYFP-HA-gSRFR1^F47T/E115A^* constructs, a ∼2 kb fragment covering eYFP-HA and SRFR1 exons 1 to 3 was subcloned to pBlueScript II SK (+) and designated as *pBS-eYFP-HA-SRFR1^Exon1–3^*. PCR-based site-specific mutagenesis was performed to make *pBS-eYFP-HA-SRFR1^Exon1–3 L13T/F47T/K101A^, pBS-eYFP-HA-SRFR1^Exon1–3 L13T/F47T^, pBS-eYFP-HA-SRFR1^Exon1–3 F47T/V110T^,* and *pBS-eYFP-HA-SRFR1^Exon1–3 F47T/E115A^*. After sequencing, ∼2 kb fragments harboring desired mutations were cut out with AatII and XbaI and ligated to AatII- and XbaI-digested *pCambia2300-eYFP-HA-gSRFR1^WT^* (see Supplementary Data Set 2). All above constructs were sequenced and transformed to *srfr1-1* and *srfr1-4* via *Agrobacterium tumefaciens* GV3101-mediated transformation. T3 homozygous plants were characterized and used for further analysis.

To make *pCambia2300-eYFP-HA-gSRFR1^IDR1-HIRD1^*, *pCambia2300-eYFP-HA-gSRFR1^IDR1-ERD14^*, *pCambia2300-eYFP-HA-gSRFR1^IDR1-COR47^*, *pCambia2300-eYFP-HA-gSRFR^p1 (E148K/E152K/D155N/D158N)^,* and *pCambia2300-eYFP-HA-gSRFR1^OsIDR1 sub^* constructs, MluI and XmaI sites were first introduced to *pBS-eYFP-HA-SRFR1^Exon1–3^* through overlap PCR to get *pBS-eYFP-HA-SRFR1^Exon1–3 MluI-XmaI^*. Then, coding sequences (without stop codon) of HIRD11, ERD14, COR47, IDR1^SRFR *p1* (E148K/E152K/D155N/D158N)^, and OsIDR1 were cut with MluI and XmaI from their *pSAT6-eYFP* constructs and ligated to MluI- and XmaI-digested *pBS-eYFP-HA-SRFR1^Exon1–3 MluI-XmaI^*. After sequencing, ∼2 kb fragments harboring desired mutations were cut out with AatII and XbaI and ligated to AatII- and XbaI-digested *pCambia2300-eYFP-HA-gSRFR1^WT^*.

To generate *pSAT6*-based transient expression SRFR1 truncation constructs, fragments corresponding to full-length SRFR1, PANT, IDR1^SRFR1^, PANT-IDR1^SRFR1^, IDR1^SRFR1^-SMiT, SMiT, SRFR1^N^ (PANT-IDR1-SMiT), SRFR1^C^ (SACT1-IDR2-SACT2), and SRFR1^Del_PANT^ (IDR1-SMiT-SACT1-IDR2-SACT2) were amplified with PCR (for primer details, see Supplementary Data Set 2), digested with KpnI and XmaI, and ligated to KpnI- and XmaI-cut *pSAT6-eYFP* (ABRC: CD3-1103) as indicated. cDNA fragments for SRFR1^Del_IDR1^ and SRFR1^Del_IDR2^ were generated by overlap PCR. The ∼1,890 bp cDNA SRFR1^Del_IDR1^ (SRFR1^Del_142–294^) fragment was obtained using primer combinations of IDR1del-CDS-LP + IDR1del-CDS-Overlap-RP, IDR1del-CDS-Overlap-LP + IDR1del-CDS-RP, and of IDR1del-CDS-LP + IDR1del-CDS-RP. The fragment was then cut with KpnI and AflII. A 1,552 bp fragment was recovered and ligated to KpnI- and AflII-cut *pSAT-eYFP-SRFR1*. ∼1,600 bp cDNA SRFR1^Del_IDR2^ (SRFR1^Del_718–754^) fragment was obtained using primer combinations of IDR2del-CDS-LP + IDR2del-CDS-Overlap-RP, IDR2del-CDS-Overlap-LP + IDR2del-CDS-RP, and IDR2del-CDS-LP + IDR2del-CDS-RP. After this fragment was cut with AflII and NotI, a ∼1,380 bp fragment was recovered and ligated to AflII- and NotI-cut *pSAT-eYFP-SRFR1*. Stop codon was incorporated into all fragments if not specified, ie, no additional sequence was added due to cloning.

To generate PANT and IDR fusion constructs, a short fragment containing MluI and XmaI was introduced to *pSAT6-eYFP-PANT-Stop* to get *pSAT6-eYFP-PANT-No Stop (NS)*. A 10 amino acid tail was added after the PANT domain due to the multiple cloning sites in *pSAT6-eYFP-PANT-NS*. Coding sequences corresponding to IDR1^NtSRFR1^, IDR1^GmSRFR1^, IDR1^ZmSRFR1^, IDR1^OsSRFR1^, IDR1^AtSRFR1^, HIRD11, XERO1, XERO2, COR47, ERD14, COR15A, LEA4-1, IDR^FLL2^, IDR^HEM1^, IDR^FCA^, and IDR1^TDP43^ were obtained by PCR. These fragments were then cut with MluI and XmaI and ligated to MluI- and XmaI-cut *pSAT6-eYFP-PANT-NS* as indicated. All constructs were confirmed by Sanger sequencing. Point mutation variants of PANT and PANT-IDR1 were generated by PCR-based site-specific mutagenesis in *pSAT6-eYFP-PANT-Stop* or *pSAT6-eYFP-PANT-IDR1^SRFR1^-NS* with primers listed in Supplementary Data Set 2. All constructs were confirmed by Sanger sequencing.

To generate *pGEX4T-3-eYFP*, *pGEX4T-3-eYFP-PANT*, *pGEX4T-3-eYFP-IDR1*, *pGEX4T-3-eYFP-PANT-IDR1*, fragments of e*YFP*, e*YFP-PANT*, e*YFP-IDR1*, and e*YFP-PANT-IDR1* were obtained by PCR using *pSAT6-eYFP*, *pSAT6-eYFP-PANT-Stop*, *pSAT6-eYFP-IDR1*, and *pSAT6-eYFP-PANT-IDR1-NS* as templates, then digested by EagI and ligated to SmaI- and EagI-cut *pGEX4T-3*. *pGEX4T-3-eYFP-6His-PANT* constructs were generated by inserting a short fragment encoding 6His between BglII and SalI in *pGEX4T-3-eYFP-PANT*. *pGEX4T-3-eYFP-6His-PANT* variants and *pGEX4T-3-eYFP-PANT*-IDR1-NS variants were generated by PCR-based site-specific mutagenesis. To generate *pCMV-*based constructs, fragments of YFP-tagged full-length SRFR1, PANT, PANT-IDR1^SRFR1^, and IDR1^SRFR1^ were amplified by PCR (for primer details, see Supplementary Data Set 2). Then, these fragments were digested with AsiSI and SmaI and ligated to the AsiSI- and PmeI-cut *pCMV* plasmid. All constructs were confirmed by Sanger sequencing.

### Protoplast isolation and transfection

Arabidopsis protoplast isolation and plasmid transfection were strictly conducted according to a previous protocol (Yoo et al. 2007). Fully expanded leaves from 5-wk-old soil-grown Col-0 were used for protoplast isolation. Leaf strips were digested with enzyme solution (1.5% Cellulase R-10, 0.4% Macerozyme R-10, 0.4 M mannitol, 20 mM KCl, 20 mM MES at pH 5.7, 10 mM CaCl_2_, and 0.1% BSA) in the dark for 3 h at room temperature. Then, the enzyme solution was diluted with an equal volume of W5 solution (154 mM NaCl, 125 mM CaCl_2_, 5 mM KCl, and 2 mM MES at pH 5.7). Protoplasts were released by gentle shaking. After washing with W5 solution, protoplasts were resuspended in MMG solution (0.4 M mannitol, 15 mM MgCl_2_, and 4 mM MES at pH 5.7) at a concentration of 2 × 10^5^ protoplasts per mL. Ten micrograms of plasmid DNA per 100 µL protoplast suspension was mixed, followed by adding 110 µL PEG solution (40% PEG4000, 0.2 M mannitol, and 100 mM CaCl_2_). After 15 min incubation, transfection was stopped by adding 440 µL W5 solution. Finally, protoplasts were resuspended in 1 mL W5 solution and incubated in 6-well plates for 12 to 16 h before confocal imaging.

Rice protoplast isolation and transfection were modified from our previous protocol (Zhang et al. 2011). Rice (*Oryza sativa*) cultivar Kitaake seeds were sterilized with 2.5% sodium hypochlorite containing 0.05% (w/v) Tween 20 for 30 min, washed with sterile water, and then sown on 1/2 MS medium plates and grown under constant light (200 µE m^−2^ s^−1^) at 30 °C for 7 to 10 d. Rice protoplasts were prepared by incubating stem strips with enzyme solution (1.5% Cellulase RS, 0.75% Macerozyme R-10, 0.6 M mannitol, 10 mM MES at pH 5.7, 10 mM CaCl_2_, and 0.1% BSA) for 6 to 8 h in the dark with gentle shaking (40 to 60 rpm). After the enzymatic digestion, an equal volume of W5 solution (154 mM NaCl, 125 mM CaCl_2_, 5 mM KCl, and 2 mM MES at pH 5.7) was added, followed by vigorous shaking by hand for 10 s. After washing with W5 solution, protoplasts were resuspended in MMG solution (0.4 M mannitol, 15 mM MgCl_2_, and 4 mM MES at pH 5.7) at a concentration of 2 × 10^6^ protoplasts per mL. Ten micrograms of plasmid DNA was used for 100 µL protoplasts. Transfection procedures were the same as for Arabidopsis protoplasts. After 12 to 16 h incubation, protoplasts were harvested for confocal imaging or Western blot.

### Root meristem analysis

Six-day-old Arabidopsis seedlings were grown on half-strength MS agar plates. Then, they were fixed in 4% PFA for 30 min. After fixation, seedlings were washed with PBS buffer. Seedlings for differential interference contrast (DIC) imaging were transferred to ClearSee reagent (10% [w/v] xylitol, 15% [w/v] sodium deoxycholate, and 25% [w/v] urea) and cleared at room temperature for 3 d. A Nikon Ti2E microscope equipped with 40× DIC optics was used to visualize cell structures beneath the epidermal layer and capture images of the cortex layer of the root. ImageJ was used for RAM length quantification. RAM length was measured as the distance from the QC to the first elongated cortical cell, while the meristematic cell number was determined by counting the cortical cells from the QC up to the first elongated cell (Ivanov and Dubrovsky 2013).

### GUS staining

Six-day-old *pCyclinB1::GUS* and *pCyclinB1::GUS srfr1^CRISPR^* seedlings grown on ½ MS agar plates were gently transferred to GUS staining solution containing 100 mM potassium phosphate buffer (pH 7.0), 2 mM K_4_Fe(CN)_6_, 2 mM K_3_Fe(CN)_6_, 0.1% Triton X-100, and 0.5 mg/mL/mL X-Gluc. Seedlings were vacuum infiltrated briefly, then incubated at 37 °C for 3 h. After staining, seedlings were washed and cleared in 70% ethanol. Visualization of blue precipitate was performed using a Nikon Ti2E microscope.

### LRC SRFR1 condensate immuno-purification and mass spectrometry

For each biological replicate, ∼2,000 seedlings per genotype (YFP-HA-tagged full-length or Del_PANT-IDR1 SRFR1) were grown vertically on ½ MS plates, with *n* = 3 for each genotype. Then, root tips (0.5 cm) were excised. Collected root tissue was fixed in 1% PFA for 15 min, then washed twice with cold PBS. The fixed tissue was dried with Kimwipes and flash-frozen in liquid nitrogen. Each sample was ground in 2 mL protein extraction buffer containing 1× PBS and 1× protease inhibitor cocktail (Sigma, P9599). Lysates were centrifuged at 4,000 × *g* for 10 min at 4 °C, and the resulting pellets were resuspended in 1 mL protein extraction buffer. The suspension was then subjected to low-speed centrifugation at 200 × *g* to remove cell debris, and the supernatant was collected. This 200 × *g* spin was repeated 3 times to enrich SRFR1 condensates. The enriched fraction was incubated with 150 µL pre-equilibrated anti-HA magnetic beads (Pierce, PI88836) for 1 h at 4 °C with gentle rotation. Beads were washed 3 times with PBST (PBS + 0.05% Tween-20) to remove nonspecifically bound proteins. Enriched SRFR1 condensates were examined by confocal microscopy.

Proteins bound to magnetic beads were resuspended in 6 M urea, 2 M thiourea, and 100 mM ammonium bicarbonate containing 5 mM DTT. Proteins were reduced for 1 h at room temperature, followed by alkylation with 14 mM iodoacetamide for 30 min in the dark. Proteins were then digested overnight at room temperature with 1 µg sequencing-grade trypsin. The supernatant containing digested peptides was collected, and digestion was quenched by adding trifluoroacetic acid (TFA) to a final concentration of 0.5%. One-fifth of each digest was used for protein quantification.

Proteomics analyses were performed using an EvoSep One liquid chromatography system coupled online to a timsTOF Pro 2 mass spectrometer (Bruker Daltonik GmbH) via a CaptiveSpray nanoelectrospray ion source. Samples were analyzed with a 44-min gradient for peptide elution (or Evosep One 30 SPD program). The chromatography setup included a 15 cm × 150 µm ID column packed with 1.5 µm C18 beads (Bruker PepSep) and a 20 µm ID zero dead volume electrospray emitter (Bruker Daltonik). Mobile phases A and B consisted of 0.1% formic acid (FA) in water and 0.1% FA in acetonitrile (ACN), respectively. The EvoSep One system was coupled online to a modified trapped ion mobility (IM) spectrometry quadrupole time-of-flight mass spectrometer (timsTOF Pro 2, Bruker Daltonik GmbH) via a nanoelectrospray ion source (Captive spray, Bruker Daltonik GmbH). To perform data-independent acquisition (DIA-PASEF), the timsTOF Pro2 operated in long-gradient DIA-PASEF mode. The method employed 16 PASEF scans, each containing 4 overlapping ion mobility (IM) and *m*/*z* windows, resulting in 64 total precursor windows covering an *m*/*z* range of 400 to 1,200 with narrow 25 *m*/*z* isolation windows and an ion mobility range of 0.6 to 1.6 Vs·cm⁻². The duty cycle was maintained at 100%, with a total cycle time of 1.8 s.

The DIA-PASEF raw data were analyzed using the directDIA workflow of Spectronaut version 20.1 with default settings (trypsin digestion with 2 allowed missed cleavages, cysteine carbamidomethylation as a fixed modification, methionine oxidation, and acetylation at the protein N-terminus as variable modifications). All searches were conducted against the Arabidopsis Uniprot database #UP000006548. For statistical analysis of the 3 replicates per genotype, the total proteome was analyzed using Spectronaut with post-analysis based on both MS levels. Candidates were filtered based on a log_2_ fold change cutoff of 0.9, a *P*-value of 0.01, and a *Q*-value of 0.05.

### Confocal microscopy

A Leica TCS SP8 laser scanning confocal microscope was used to image protoplasts, live tissues, and in vitro fibrils and condensates. For Arabidopsis protoplast imaging, the excitation wavelength for YFP and chlorophyll auto-fluorescence was 514 nm with 10% laser power. 2% laser power was used for fibril imaging. The emission wavelengths for eYFP and chlorophyll auto-fluorescence were 525 to 575 nm and 650 to 750 nm, respectively. For rice protoplast imaging, the excitation wavelength for YFP was 514 nm with 2% laser power. The emission wavelength for eYFP was 525 to 575 nm. For in vitro fibrils and condensates imaging, a YFP laser line at 514 nm with 0.4% laser power was used. PI staining of Arabidopsis root was performed by immersing whole seedlings in PI staining solution (30 µg/mL PI in liquid ½ MS medium, pH 5.7) for 2 min. The roots were washed in ½ MS medium and subjected to confocal imaging. A 536 nm laser line with 10% laser power was used for PI excitation, and 600 to 680 nm wavelengths were used to detect the PI emission signal. Six-day-old seedlings of e*YFP-HA-gSRFR1^WT^* line #7 were treated with ACC (1 µM), AVG (1 µM), IBA (1 µM), IAA (1 µM), GA (0.1 µM), and BAP (0.1 µM) for 12 h prior to confocal imaging.

For large-scale root condensate imaging, seedlings were fixed in 4% PFA for 30 min. After fixation, seedlings were washed twice with PBS and kept in PBS at 4 °C until imaging. Due to the limited scanning depth of confocal microscopy, we quantified condensates per-half-root, scanning from the outer surface to the midsection at 0.42 µm intervals. In general, 50 to 70 images along the *z*-axis were obtained for each half-root. The *z*-stack images were reconstructed into 3D images with the Leica LAS X application suite. The reconstructed 3D images were then aligned to match the original scanning orientation. Then, the height and the diameter of each condensate were measured with ImageJ, and given the column-like morphology of SRFR1 condensates, their volume was calculated by approximating their shape as cylinders.

### Recombinant protein isolation and hydrodynamic radius measurement

To ensure high expression of recombinant proteins, single colonies were picked from freshly transformed *E. coli* BL21(DE3) pLysS plates. Around 10 intermediate-sized single colonies were mixed for inoculation and then incubated overnight at 37 °C in LB broth. For the purification of GST-eYFP-PANT and GST-eYFP-6His-PANT, 3 L of LB were inoculated with 20 mL of overnight culture and incubated for approximately 3 h to reach an optical density of 0.4 to 0.5. This cell culture was then incubated on ice for 1 h. IPTG, final concentration 200 μM, was added to the cell culture. After growing overnight at 22 °C, the cell culture was passed through a French press to lyse the cells. The extract was centrifuged, and 3 mL of glutathione agarose resin (GoldBio, cat# G-250) was added to 150 mL supernatant. Samples were incubated at 4 °C for 1 h with rotation and then washed with PBS in a gravity column. Protein was eluted from beads with 10 mL of 50 mM reduced glutathione (prepared with PBS, pH adjusted to 7.4). Protein was diluted with PBS and then concentrated with Amicon centrifugal filters (10 kDa cutoff) several times until the concentration of glutathione was below 0.5 mM. Purity and the correct size of purified proteins were confirmed by SDS-PAGE and Coomassie blue staining. Because PANT is an aggregation-prone protein when expressed alone, less than 20% is soluble. For this reason, large volumes of cell culture and glutathione agarose resin were used. Still, about 50% GST-eYFP-PANT aggregated during the concentration step. Moreover, fine fibrils of GST-eYFP-PANT only form with freshly prepared protein. After freezing, even in the presence of 25% glycerol, only aggregation-like structures formed. For purification of GST-eYFP, GST-eYFP-PANT-IDR1, as well as variants of GST-eYFP-6His-PANT and GST-eYFP-PANT-IDR1, about one-third of the cell culture and glutathione agarose resin were used. The hydrodynamic radius of purified proteins at different temperatures was determined with multiple-angle dynamic light scattering (MADLS) using the Zetasizer Ultra (Red) (Malvern Panalytical Ltd.). One hundred and sixty microliters of protein sample at a concentration of 30 µM was loaded into a ZEN2112 cuvette. Protein samples were equilibrated at the indicated temperatures for 5 min, then MADLS measurements were collected at 3 different angles of detection (173°, 90°, and 13°). MADLS data were processed by the ZS Xplorer software version 3.0 (Malvern Panalytical Ltd.).

### In vitro fibril and condensate assay

For the in vitro fibril and condensate assay, proteins were all prepared freshly and diluted to the desired concentrations. Then, these proteins were centrifuged for 15 min at 4 °C, 21,000 × *g* to remove protein aggregates formed during concentration steps. The in vitro fibril and condensate assay was performed in a 200 µL PCR tube at room temperature by mixing with 10 × PEG8000 stock solution. To ensure that PEG8000 was completely dissolved, the PEG solution was freshly prepared with the indicated buffers was shaken at 40 to 60 rpm for at least 1 h. For example, 40 µL of 30 µM GST-eYFP-PANT was added to a PCR tube containing 4 µL of 10% PEG8000 and mixed by gentle tapping and incubated for 3 h at the indicated temperatures. Then, these whole mixtures were transferred to 18-well µ-Slides (Ibidi, cat#81816) for confocal imaging.

### Total RNA isolation and RT-qPCR analysis

Total RNA from young seedlings of Arabidopsis, rice (*Oryza sativa*), soybean (*Glycine max*), maize (*Zea mays*), tobacco (*Nicotiana tabacum*), and human (*Homo sapiens*) HEK293T cells was extracted using TRIzol reagent (Invitrogen). Endogenous RNase was further removed by ethanol precipitation. After DNase treatment, 1 µg of total RNA was used for first-strand cDNA synthesis with M-MLV reverse transcriptase (Promega) and oligo (dT) 18 primer. Real-time quantitative PCR (RT-qPCR) was performed using SYBR Green qPCR Master Mix (Agilent) in an ABI 7500 real-time qPCR machine (Life Technologies). *EF1a* was used as an internal control. Primers used for RT-qPCR are listed in Supplementary Data Set 2.

### Root measurement

Roots of Arabidopsis seedlings were aligned on the surface of Agar plates. Then, images were captured with a digital camera. The length of primary roots was measured using ImageJ software. To avoid experimental errors, seeds were harvested from plants grown side by side, and different genotypes were grown in the same Petri dish with multiple replicates.

### Protein structure prediction

PANT dimer and tetramer structures were modeled by a locally installed AlphaFold v2.2.4 using default settings and the AlphaFold3 server (Jumper et al. 2021; Abramson et al. 2024). Interestingly, the predicted tetramer structure contains 2 modeled PANT dimeric structures, ie, the modeled PANT dimeric interface was retrieved in the predicted tetramer structure. Therefore, the structure of the 24-mer PANT polymer was generated by the elongation of the tetramer using the MatchMaker in the program UCSF Chimera (Pettersen et al. 2004). Structures of peptides, proteins, and protein-peptide complexes were analyzed and displayed using UCSF Chimera X (Pettersen et al. 2021).

### Sequence analysis of IDRs and IDPs

The blockiness of the like charges parameter (B_LC_) was calculated exactly by following the mathematical formula described by Yamazaki et al. (2022). The local NCPR was calculated for 10 amino acid sliding windows, followed by the description by Lyons et al. (2023). The FCR, kappa, and global NCPR parameters were calculated using the CIDER server (Holehouse et al. 2017). Protein sequences for IDRs and IDPs are listed in Supplementary Data Set 3.

### Immunoblot

Immunoblot analysis of the expressed HA-tagged SRFR1 variants was performed by extracting approximately 0.15 g of whole seedlings in 0.3 mL of 2 × sodium dodecyl sulfate (SDS) loading buffer (100 mM Tris–HCl, pH 6.8, 4% w/v SDS, 20% v/v glycerol, 30 mM dithiothreitol). For the detection of GFP-tagged SRFR1 and DVL2 variants in rice protoplasts, 0.3 mL of 2 × SDS loading buffer was added to 100 µL of the protoplast pellet. Samples were cleared by centrifugation at 21,000 × *g* for 15 min at 4 °C and loaded onto an 8% v/v SDS-PAGE gel. Protein was detected with 1:3,000 horseradish peroxidase-conjugated anti-HA (Roche, cat#12013819001) or anti-GFP (Invitrogen, A11122) antibodies.

### Statistical analysis

All statistical analyses were performed using GraphPad Prism 10. Root length values, which were assumed to follow a normal distribution, were analyzed using ordinary one-way ANOVA with Tukey's test for multiple comparisons. For two-sample comparisons, an unpaired *t*-test with Welch's correction was used, as equal standard deviation among genotypes was not assumed. Condensate-related measurements did not follow a normal distribution; therefore, nonparametric tests were applied. Kruskal–Wallis Dunn's post-hoc tests were used for multiple-group comparisons, and Mann–Whitney *U* tests were used for two-sample comparisons. Additional details, including sample size and exact *P*-values, are provided in Supplementary Data Set 4.

### Accession numbers

Protein sequences in this study can be found in The Arabidopsis Information Resource (TAIR, www.arabidopsis.org) and UniProt (www.uniprot.org). SRFR1 (AT4G37460), ETR1 (AT1G66340), EIN2 (AT5G03280), SNC1 (AT4G16890), EDS1 (AT3G48090), HIRD11 (AT1G54410), XERO1 (AT3G50980), XERO2 (AT3G50970), ERD14 (AT1G76180), COR47 (AT1G20440), COR15A (AT2G42450), LEA4-1 (AT1G32560), FLL2 (AT1G67170), HEM1 (AT2G35110), FCA (AT4G16280), ACS1 (At3g61510), ACS2 (At1g01480), ACS4 (At2g22810), ACS6 (At4g11280), ACS7 (At4g26200), ACS8 (At4g37770), ACS9 (At3g49700), ACS11 (At4g08040), EF1a (At5g60390), DVL2 (UniProt: O14641), TDP-43 (UniProt: Q13148). The IP-MS data have been deposited to the ProteomeXchange Consortium via the PRIDE partner repository with the dataset identifier PXD069457.

## Supplementary Material

koaf292_Supplementary_Data

## Data Availability

Constructs and transgenic plants generated in this study will be available to the scientific community from W.G. upon request. This paper does not report original code. Any additional information required to reanalyze the data reported in this paper is available from W.G. upon request.

## Supplementary material

The following materials are available in the online version of this article.

**Supplementary Figure S1**. Rescue of the *srfr1* short root phenotype by inhibiting ethylene biosynthesis and enhanced root growth via SRFR1 overexpression (supports Fig. 2).

**Supplementary Figure S2**. Subcellular localization of SRFR1 in root and leaf tissues (supports Fig. 3).

**Supplementary Figure S3**. Subcellular localization of different domains of SRFR1 (supports Fig. 5).

**Supplementary Figure S4**. PANT domain-mediated polymerization is required for upper LRC SRFR1 condensate formation and primary root growth (supports Figs. 6 and 7).

**Supplementary Figure S5**. In vitro sedimentation of PANT variants (supports Fig. 6).

**Supplementary Figure S6**. PANT domain polymerization-mediated upper LRC SRFR1 condensate formation and primary root growth (supports Fig. 7).

**Supplementary Figure S7**. In vitro condensation assay (supports Fig. 8).

**Supplementary Figure S8**. Summary of the subcellular localization of 57 PANT-IDR1 variants in rice protoplasts (supports Fig. 9).

**Supplementary Figure S9**. Subcellular localization of PANT-IDR1 variants and charge distribution of IDR1^SRFR1^ orthologs (supports Fig. 9).

**Supplementary Figure S10**. Functional substitution of the IDR1 domain of SRFR1 with its orthologs (supports Fig. 10).

**Supplementary Figure S11**. Association with zwitterionic IDRs is common among polymerization domains (supports Figs. 9 and 10).

**Supplementary Figure S12**. Digital expression patterns of putative SRFR1 condensate components in different root tissues (supports Fig. 12).

**Supplementary Data Set 1**. List of SRFR1 condensate-associated proteins identified by IP-MS.

**Supplementary Data Set 2**. List of DNA primers and transgenic or gene-edited plant lines.

**Supplementary Data Set 3**. Sequences used for charge patterning analysis.

**Supplementary Data Set 4**. Statistical analysis details.

**Supplementary Video 1**. SRFR1 forms condensates in upper lateral root cap cells (supports Fig. 1j).

**Supplementary Video 2**. SRFR1 forms condensates in upper lateral root cap cells (supports Fig. 1l).

**Supplementary Video 3**. The PANT domain forms fibrils in Arabidopsis and rice protoplasts (supports Fig. 3b).

**Supplementary Video 4**. Purified GST-YFP-PANT forms fibrils in vitro (supports Fig. 4b).

## Dive Curated Terms

The following phenotypic, genotypic, and functional terms are of significance to the work described in this paper:

RIN4 Gramene: AT3G25070
RIN4 Araport: AT3G25070

SRFR1 Gramene: AT4G37460
SRFR1 Araport: AT4G37460

PIN5 Gramene: AT5G16530
PIN5 Araport: AT5G16530

PIN1 Gramene: AT1G73590
PIN1 Araport: AT1G73590

PIN3 Gramene: AT1G70940
PIN3 Araport: AT1G70940

PIN4 Gramene: AT2G01420
PIN4 Araport: AT2G01420

EIN2 Gramene: AT5G03280
EIN2 Araport: AT5G03280

root AmiGo: PO:0009005
