# Supplementary material for: Polymerization-mediated SRFR1 condensation in upper lateral root cap cells regulates root growth
Source: Plant Cell. 2025 Dec 30;38(1):koaf292. doi: 10.1093/plcell/koaf292 (PMC12862871; doi:10.1093/plcell/koaf292)
Supplement: koaf292_Supplementary_Data [file koaf292_supplementary_data.zip › Supplemental Figures 121725_JS_wg clean.pdf]

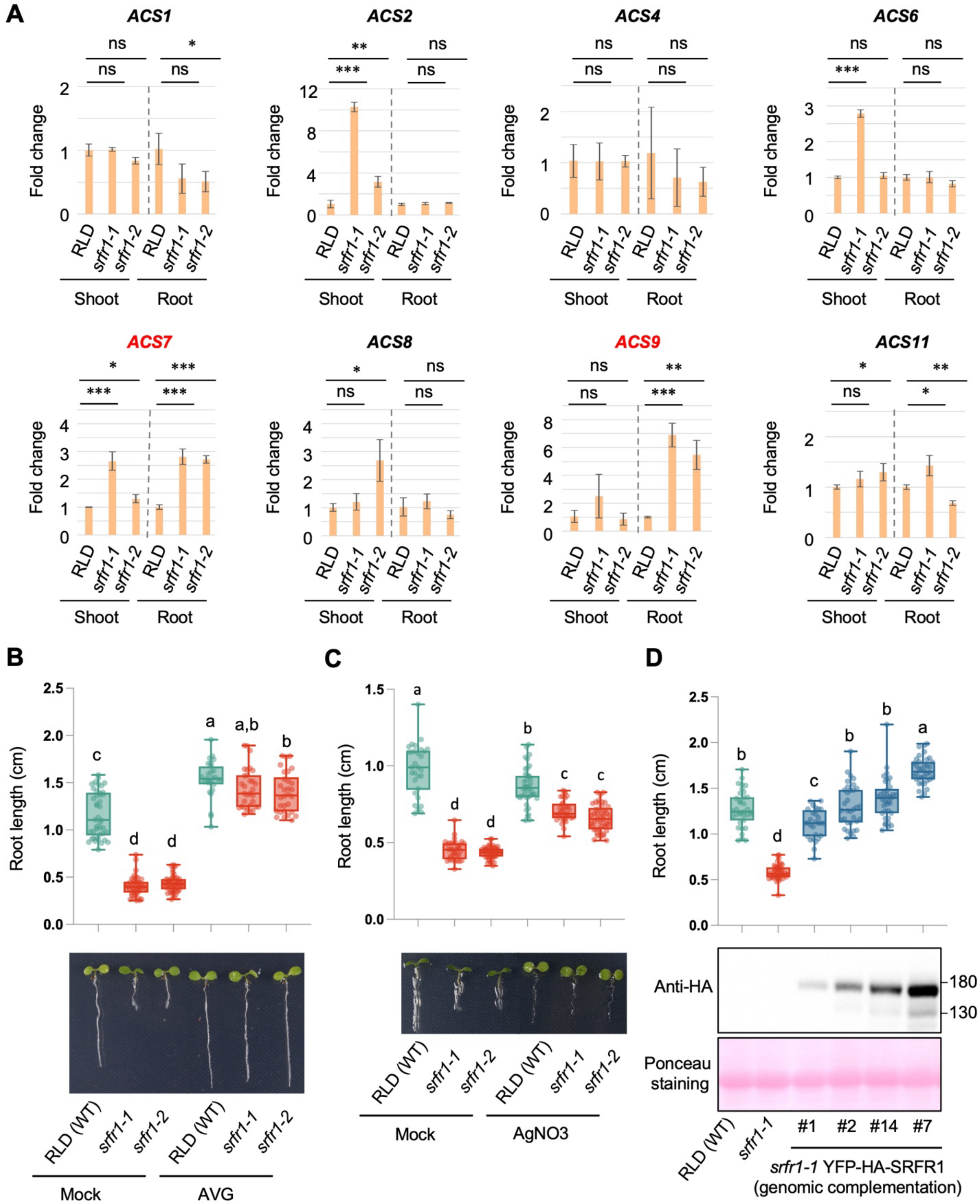

Supplementary Fig. S1. Rescue of *srfr1* short root phenotype by inhibiting ethylene biosynthesis and enhanced root growth via SRFR1 overexpression (supports Fig. 2). **A)** Real-time qPCR analysis of ethylene biosynthesis gene expression in the shoot and root tissues of RLD, *srfr1-1*, and *srfr1-2*. Values are means ± SD, n = 3. *EF1a* was used

as an internal control. *** indicates $p < 0.001$, ** indicates $p < 0.01$ and * indicates $p < 0.05$ (Unpaired Student's t-test). **B** and **C)** The short root phenotype of *srfr1-1* and *srfr1-2* is largely rescued by inhibiting ethylene biosynthesis. Primary root length was measured with 6-day-old seedlings grown on ½ MS plates containing the indicated concentrations of $AgNO_3$ and AVG. n= 30-45 for AgNO3 treatment, and n = 26-49 for AVG treatment, respectively. **D)** High expression of YHP-HA-tagged SRFR1 enhances primary root growth. Primary root length was measured with 6-day-old seedlings, n = 27-42. Protein expression levels were detected using an anti-HA antibody. Ponceau S staining of the Rubisco large subunit was used as protein loading control. For **B**-**D)**, all data points are shown in box-and-whisker plots. Boxes indicate the interquartile range, with center lines denoting the median. Whiskers extend to the minimum and maximum values of the dataset. Letters indicate statistically different groups (ordinary one-way ANOVA, Tukey-Kramer grouping).

ALT TEXT: Images and bar graphs show *srfr1* mutants exhibit shortened roots, which can be rescued by mutations in the ethylene signaling pathway. Image and bar graphs in Panel C and D show SRFR1 overexpression enhances root growth.

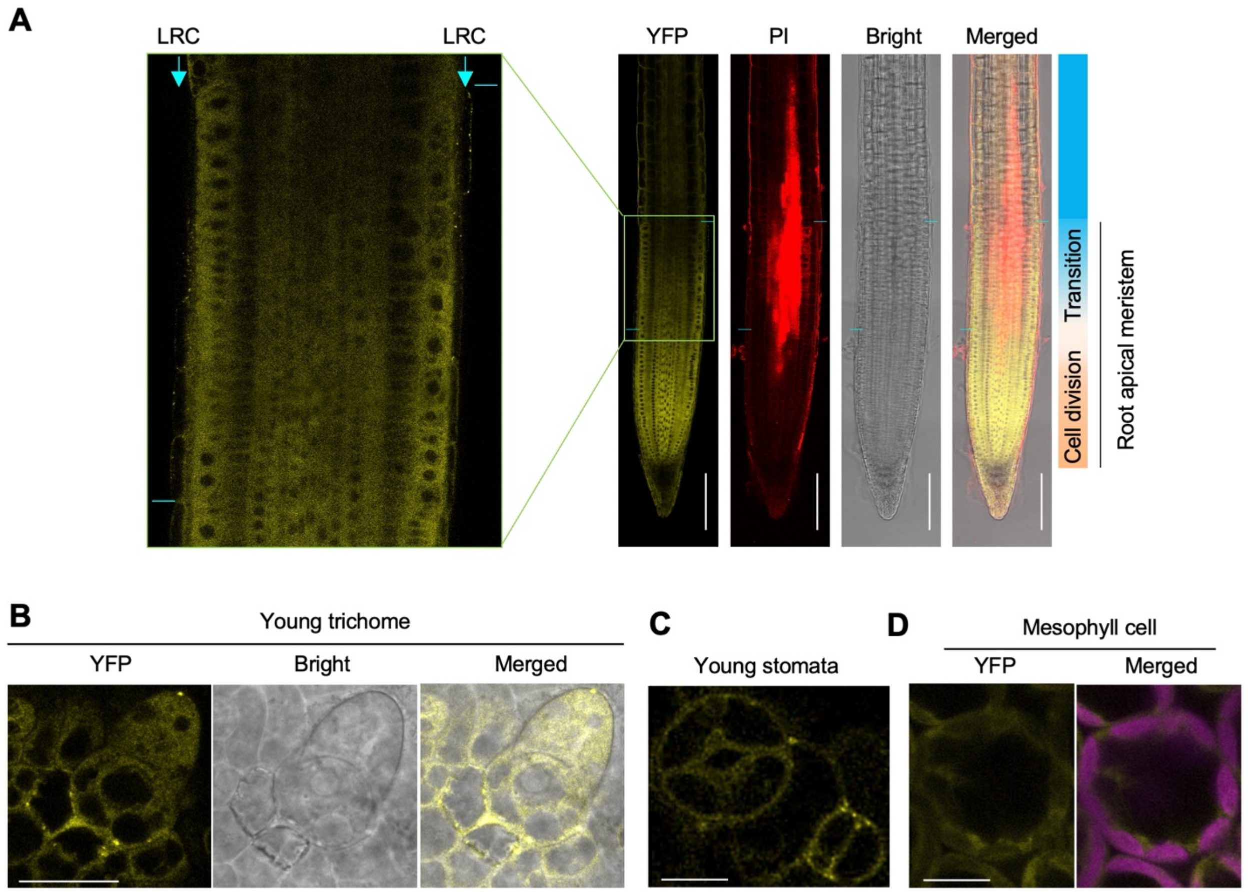

Supplementary Fig. S2. Subcellular localization of SRFR1 in root and leaf tissues (supports Fig.3). **A)** Localization of SRFR1 condensates in roots of 6-day-old seedlings. Images were obtained using tile scanning and assembled from 4 median section images of the same root shown in Fig. 3B (assembled surface sections). Scale bar = 100μm. **B-D)** Subcellular localization of SRFR1 in the true leaf tissues of 8-day-old seedlings. Merged chlorophyll auto-fluorescence and YFP signals were also shown. Bar = 10 μm.

ALT TEXT: Microscopy images show SRFR1 protein localization (yellow) in different plant root and leaf cell types: trichome cells (A), stomata (B), and mesophyll cells (C).

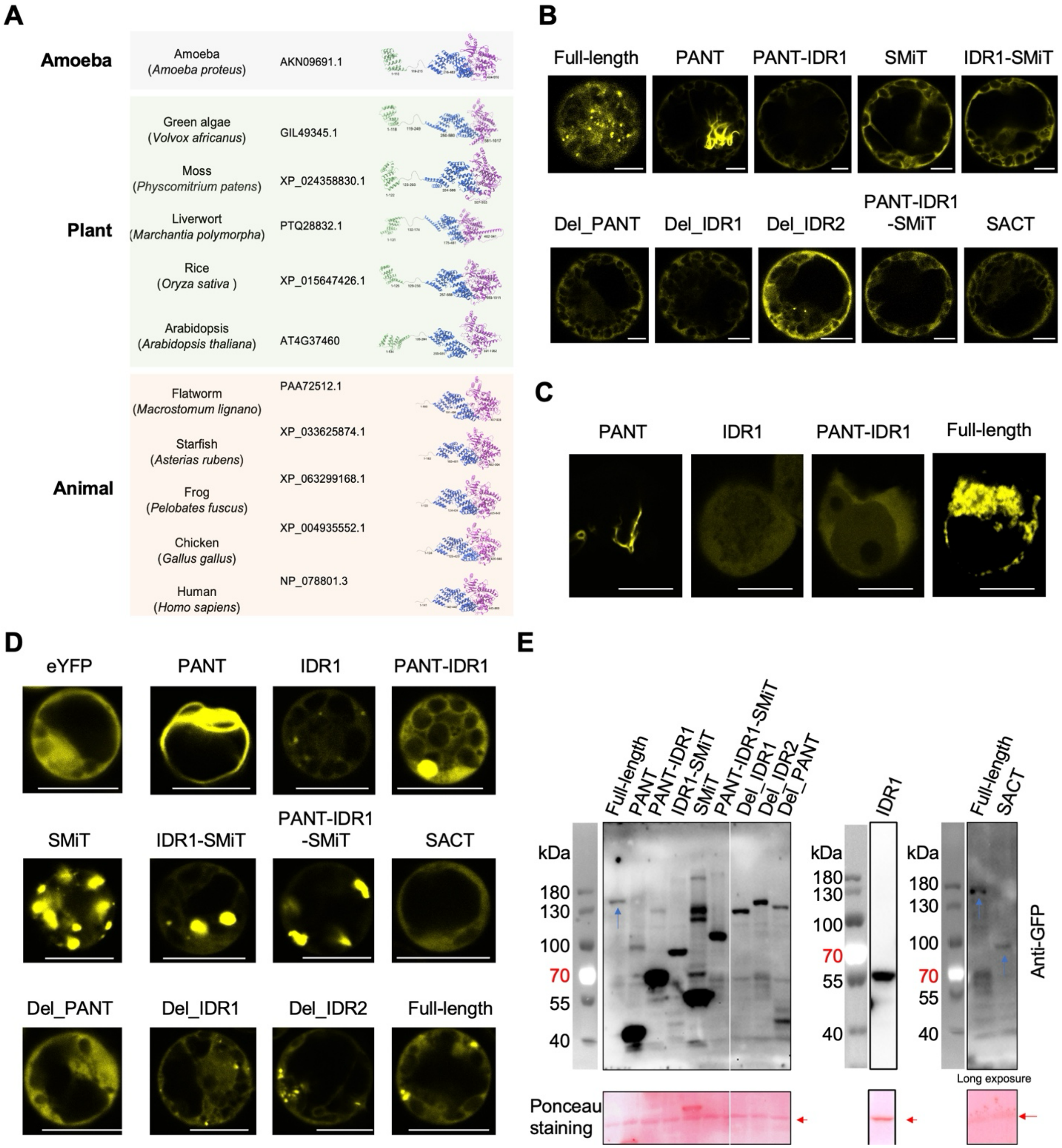

Supplementary Fig. S3. Subcellular localization of different domains of SRFR1 (supports Fig.5). **A)** The PANT domain is absent in SRFR1 orthologs in animals. Protein architecture is drawn based on AlphaFold2 predictions. **B)** The PANT domain is required for SRFR1 biomolecular condensate formation in Arabidopsis protoplasts. SRFR1 and differentially truncated SRFR1 variants were expressed in Arabidopsis protoplasts. **C)** Subcellular localization of PANT, IDR1, PANT-IDR1, and full-length SRFR1 in human HEK293T cells. Bar = 10 μm. **D)** Subcellular localization of SRFR1 full-length and variants in rice protoplasts. Bar = 10 μm. Images were taken using the same confocal microscopy settings. **E)** Western blot detection of YFP-tagged full-length and truncated

SRFR1 expression in rice protoplasts. Ponceau S staining of the Rubisco large subunit is shown as a protein loading control. Blue arrows indicate lower protein accumulation of $SRFR1^{FL}$ and SACT.

ALT TEXT: Panel A highlights the absence of the PANT domain in animal SRFR1 orthologs. Images show subcellular localization of SRFR1 proteins with varying domain compositions (B-D), and expression confirmation by Western blot (E).

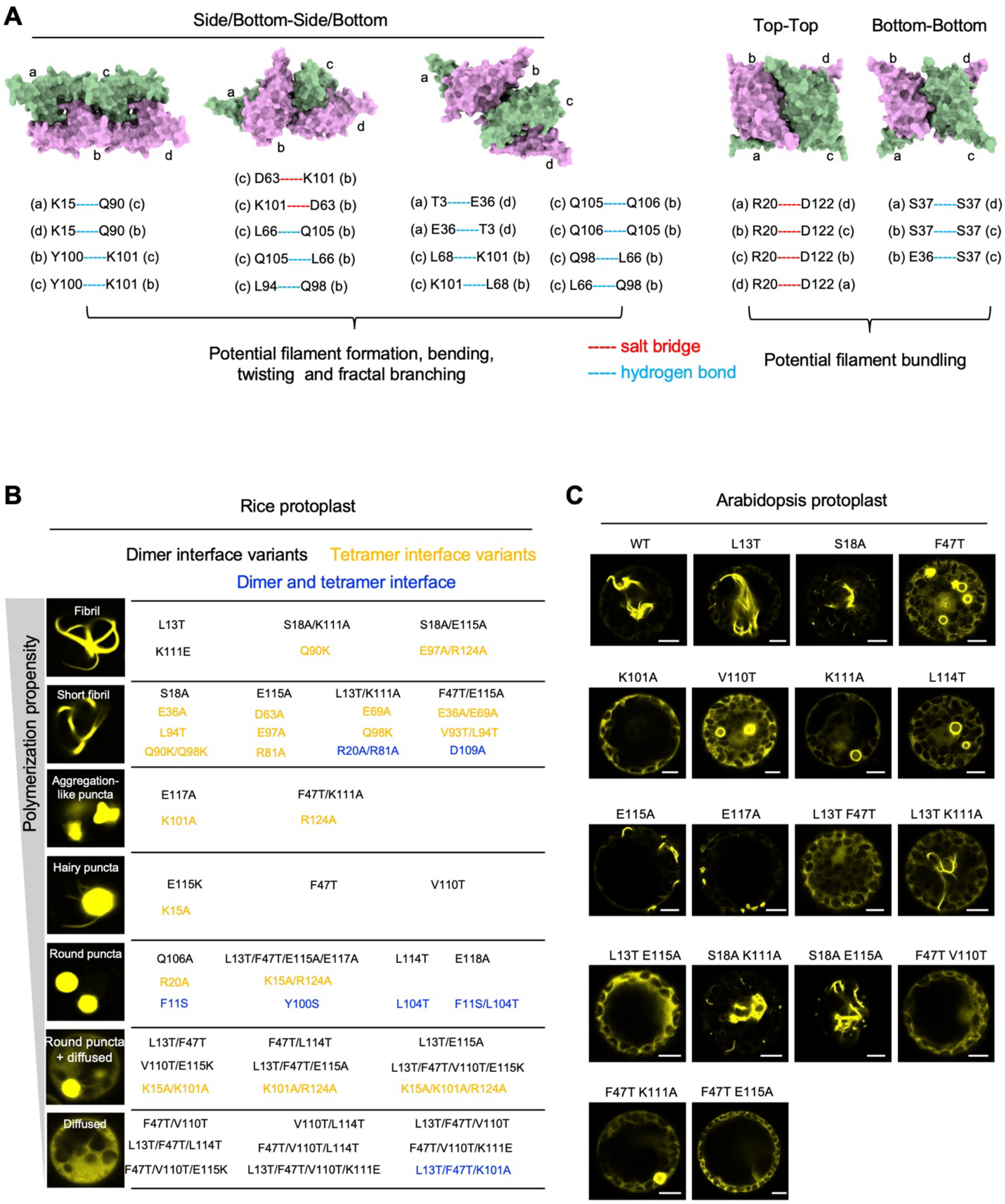

Supplementary Fig. S4. PANT domain-mediated polymerization is required for upper LRC SRFR1 condensate formation and primary root growth (supports Fig. 6 and Fig.7). **A)** PANT tetramer prediction using both a locally installed AlphaFold2 and the AlphaFold3 server. Five representative models are shown. **B)** Summary of the subcellular localization of 58 PANT dimer and tetramer interface variants in rice

protoplasts. Images were taken using the same confocal microscopy settings. Some of the representative images have been reused from Fig. 6H to more clearly illustrate the distinct polymerization morphologies of the different PANT variants. **C)** Subcellular localization of 17 PANT variants in Arabidopsis protoplasts. Images were taken using the same confocal microscopy settings.

ALT TEXT: Panel A shows predicted PANT protein tetramer structures with different interaction interfaces. Panel B categorizes 58 PANT variants by their localization patterns in rice protoplasts. Panels C shows the subcellular localization of 17 PANT variants in Arabidopsis protoplasts.

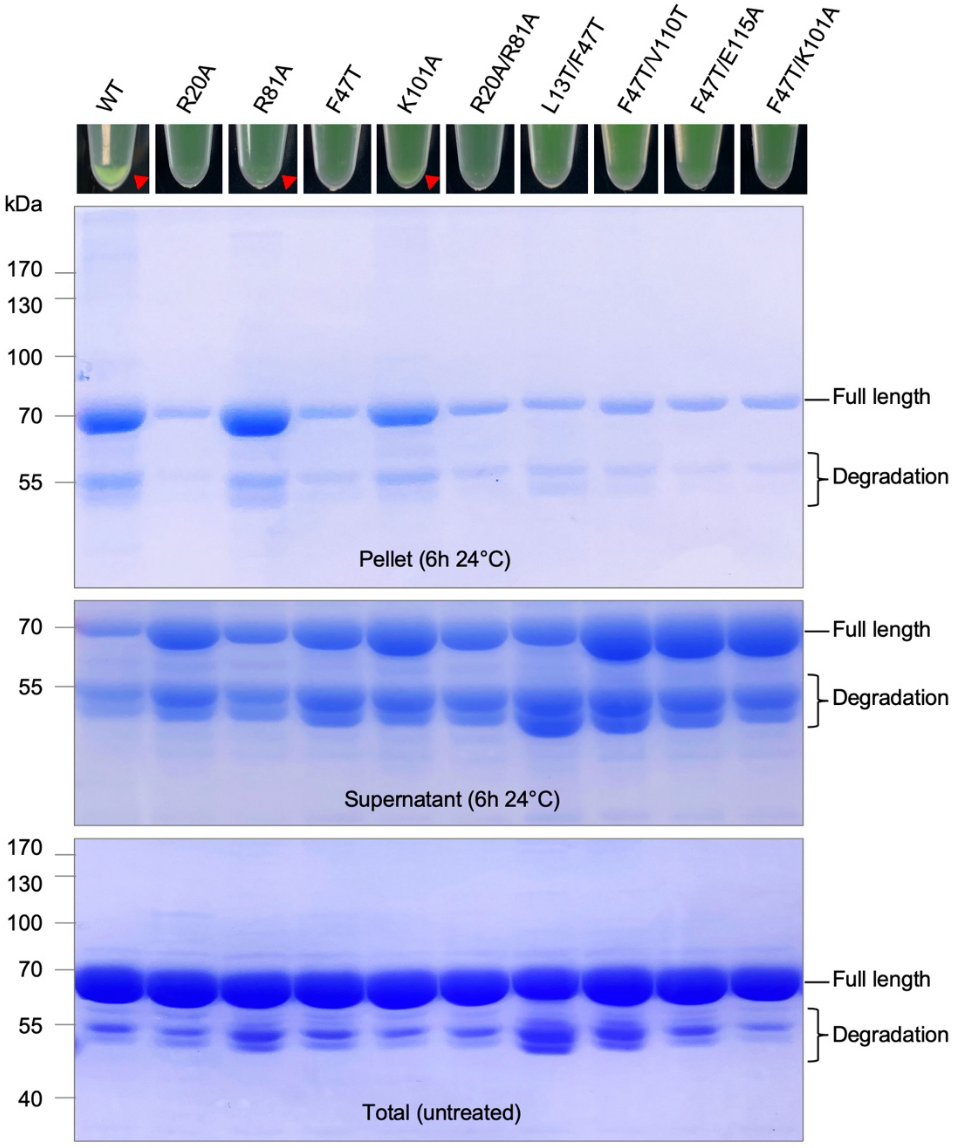

Supplementary Fig. S5. *In vitro* sedimentation of PANT variants (supports Fig. 6). GST-eYFP tagged PANT and PANT variants at 30 μM in PBS solution were incubated at room temperature for 6 hours.

ALT TEXT: This image shows protein sedimentation analysis comparing the wild-type PANT domain with 9 variants. Gels show protein distribution between pellet and supernatant fractions after centrifugation, revealing how different mutations affect protein aggregation properties.

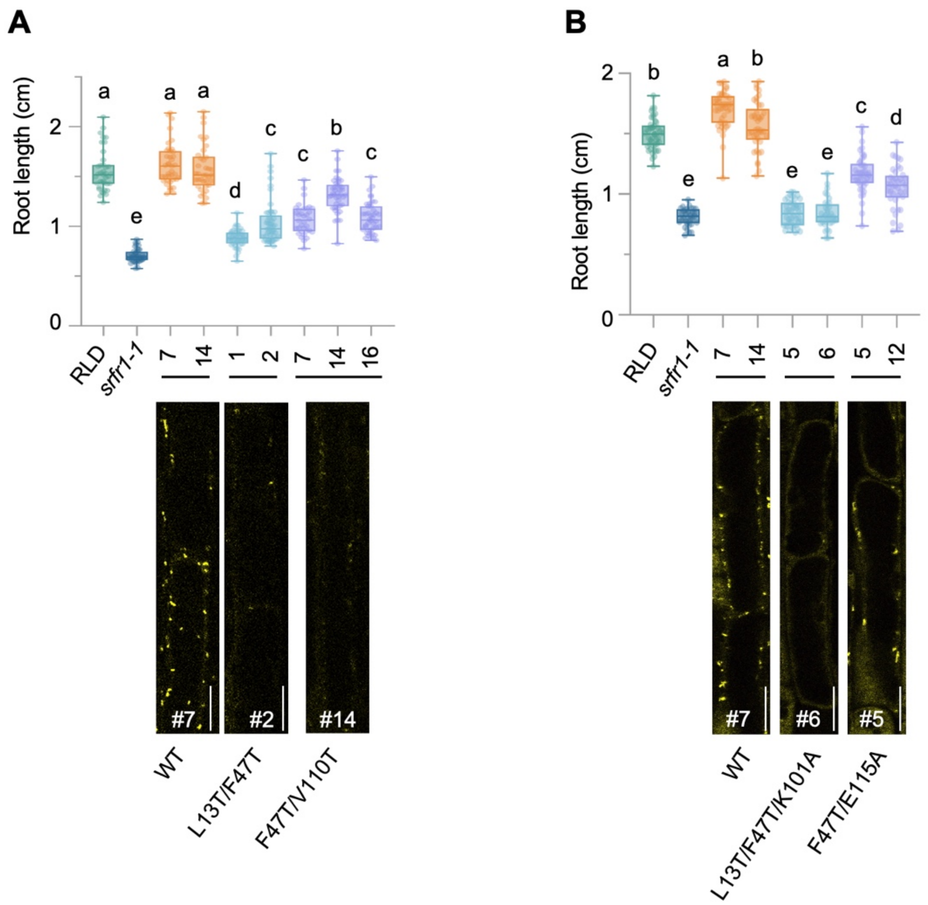

Supplementary Fig. S6. PANT domain polymerization-mediated upper LRC SRFR1 condensate formation and primary root growth (supports Fig.7). **A** and **B)** Primary root length and upper LRC condensate accumulation of YFP-HA-tagged SRFR1 dimer-interface variants. Primary root length was measured with 6-day-old seedlings, n = 38-53. All data points are shown in box-and-whisker plots. Boxes indicate the interquartile range, with the center line denoting the median. Whiskers extend to the minimum and maximum values of the dataset. Letters denote statistically different groups (ordinary

one-way ANOVA, Tukey-Kramer grouping). Images were taken with roots of 6-day-old seedlings. Single surface section confocal images were shown. Bar = 10 μm.

ALT TEXT: Graph and images show how mutations affecting PANT polymerization impact root growth and SRFR1 condensate formation in upper LRCs.

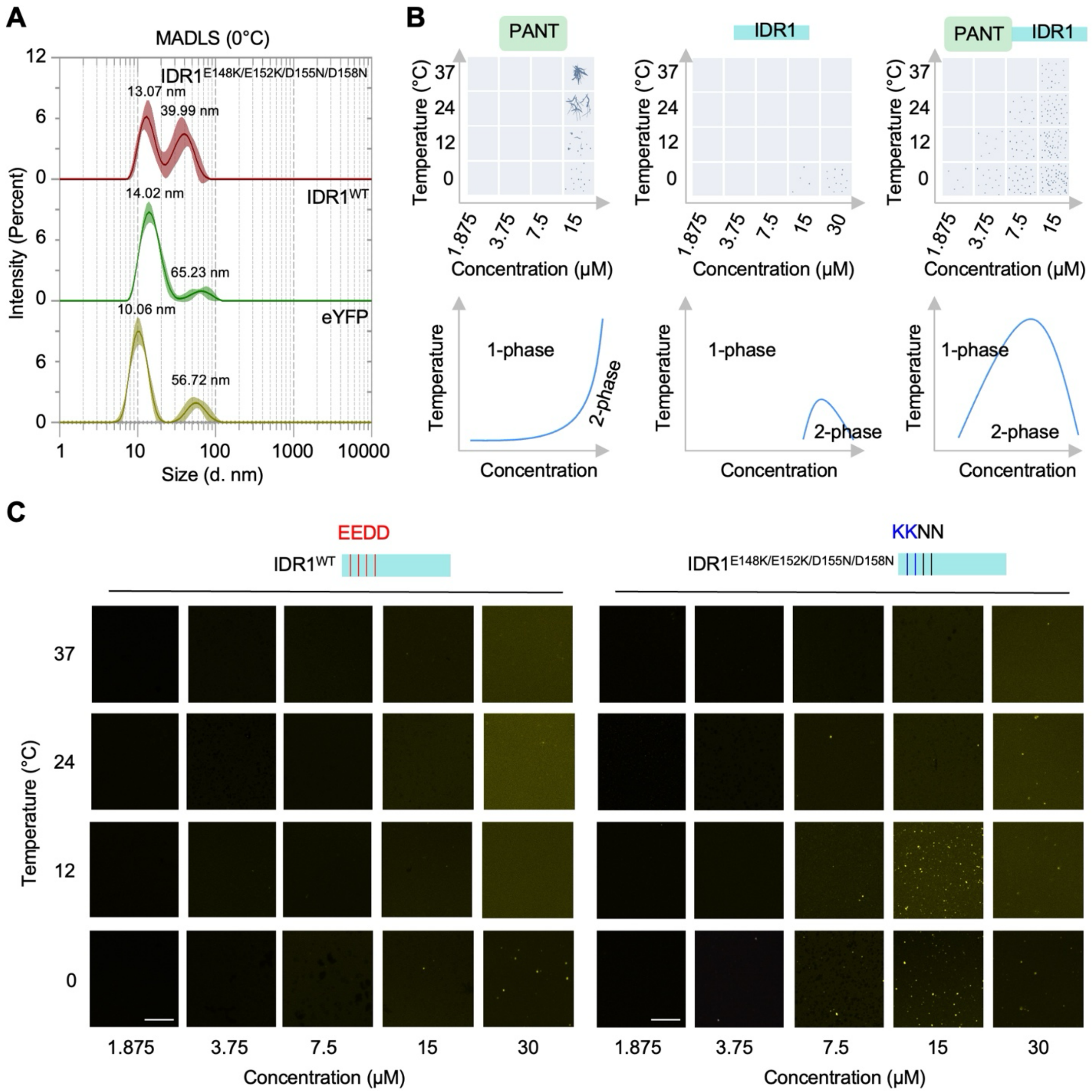

Supplementary Fig. S7. *In vitro* condensation assay (supports Fig. 8). **A)** The hydrodynamic radius of GST-YFP, GST-YFP-IDR1^{WT} and GST-YFP-IDR1$^{E148K/E152K/D155N/D158N}$ at 30 μM was measured in PBS solution at 0°C. **B)** *In vitro* condensate formation assay of GST-eYFP tagged PANT, IDR1, and PANT-IDR1 in PBST buffer containing 0.5% PEG8000 (w/v) at different concentrations and temperatures. Schematic diagrams are shown based on experimental data. **C)**

Condensate formation by GST-YFP tagged IDR1^{WT} and IDR1$^{E148K/E152K/D155N/D158N}$ in PBST buffer containing 0.5% PEG8000 (w/v). Images were taken 3 hours post PEG addition using the same confocal microscopy settings. Bar = 10 μm.

ALT TEXT: This figure compares protein condensation behaviors at different temperatures and concentrations. Panel A displays particle size measurements. Panel B shows phase diagrams of PANT, IDR1 and PANT-IDR1, and panel C compares wild-type IDR1 with a variant showing different condensation patterns across temperatures and concentrations.

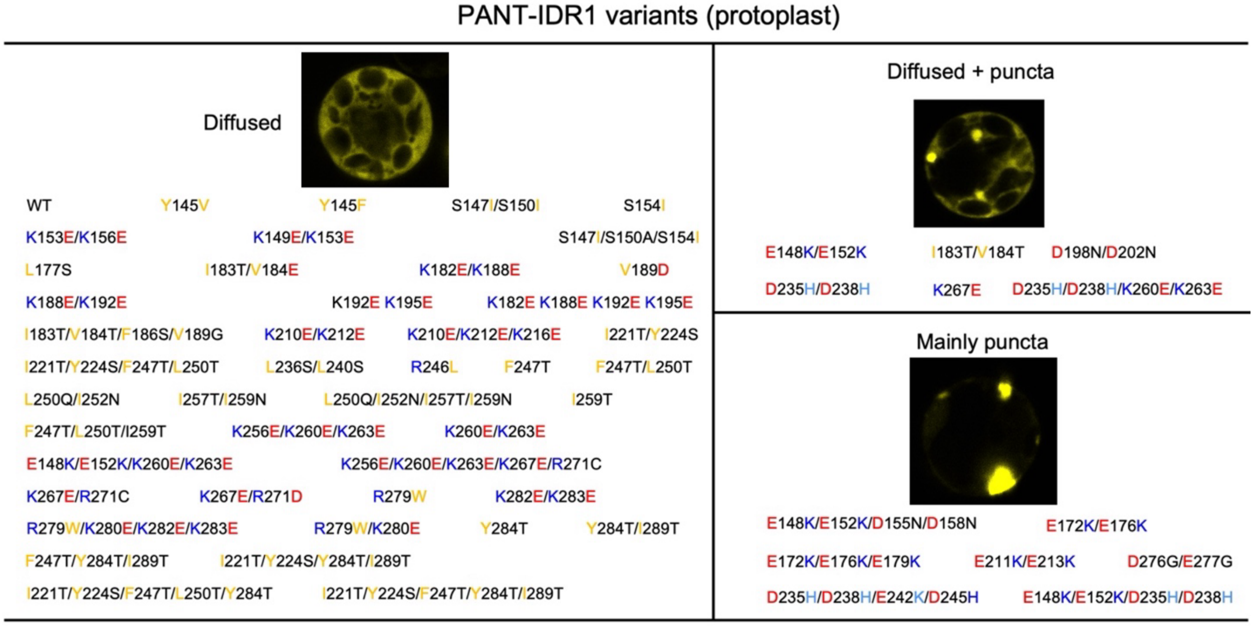

Supplementary Fig. S8. Summary of the subcellular localization of 57 PANT-IDR1 variants in rice protoplasts (supports Fig. 9). Images were taken using the same confocal microscopy settings. Note: The PANT-IDR1 construct here includes an additional six amino acids (PGIHLI) at its C-terminus due to an introduced restriction enzyme site, resulting in diffuse localization (*pSAT6-eYFP-PANT-IDR1$^{No\ Stop}$*, Supplementary Dataset 1). In contrast, the PANT-IDR1 construct in Supplementary Fig. S3 contains a stop codon immediately following the IDR1 sequence (*pSAT6-eYFP-PANT-IDR1^{Stop}*, Supplementary Dataset 1). We take advantage of the diffuse localization of PANT-IDR1 (No Stop) to screen variants that impair or suppress PANT polymerization.

ALT TEXT: This figure shows subcellular localizations of 57 PANT-IDR1 variants in rice protoplasts, categorized as diffused, diffused + puncta, or mainly puncta.

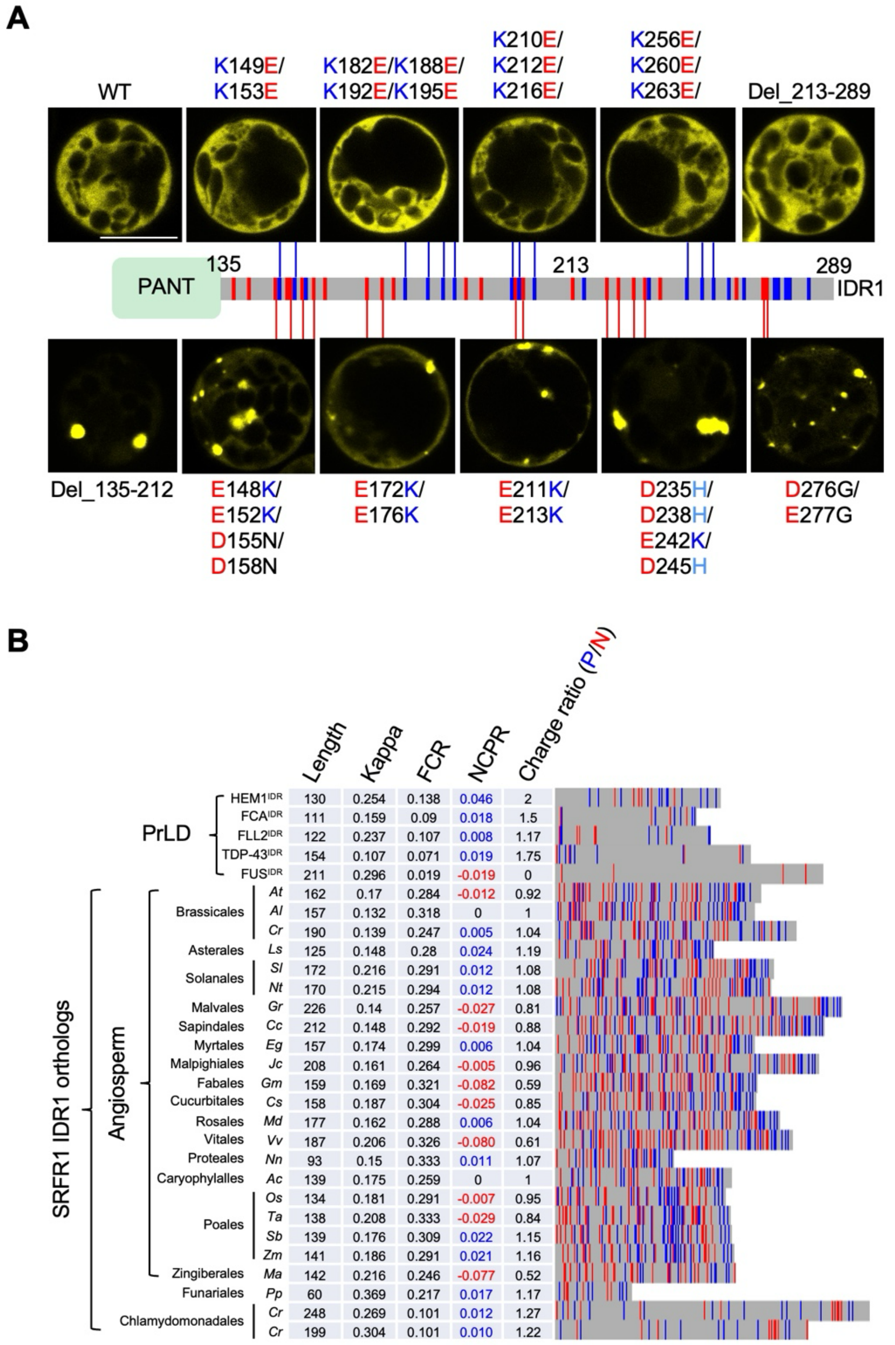

Supplementary Fig. S9. Subcellular localization of PANT-IDR1 variants and charge distribution of IDR1^{SRFR1} orthologs (supports Fig. 9). **A)** Subcellular localization of PANT-IDR1 variants in rice protoplasts. Images were taken using the same confocal microscopy settings. Bar = 10 µm. Note: The same as in Supplementary Fig. S8, *pSAT6-eYFP-PANT-IDR1$^{No\ Stop}$*, was used. **B)** Diagram of the arrangement of amino

acids in PrLDs and IDR1 orthologs. Positively charged amino acids are colored in blue, negatively charged amino acids are colored red, and others are shown in gray.

ALT TEXT: Panel A shows the localization of PANT-IDR1 charge variants in rice protoplasts. Bars in panel B depict charge distributions in SRFR1 IDR1 orthologs and PrLDs.

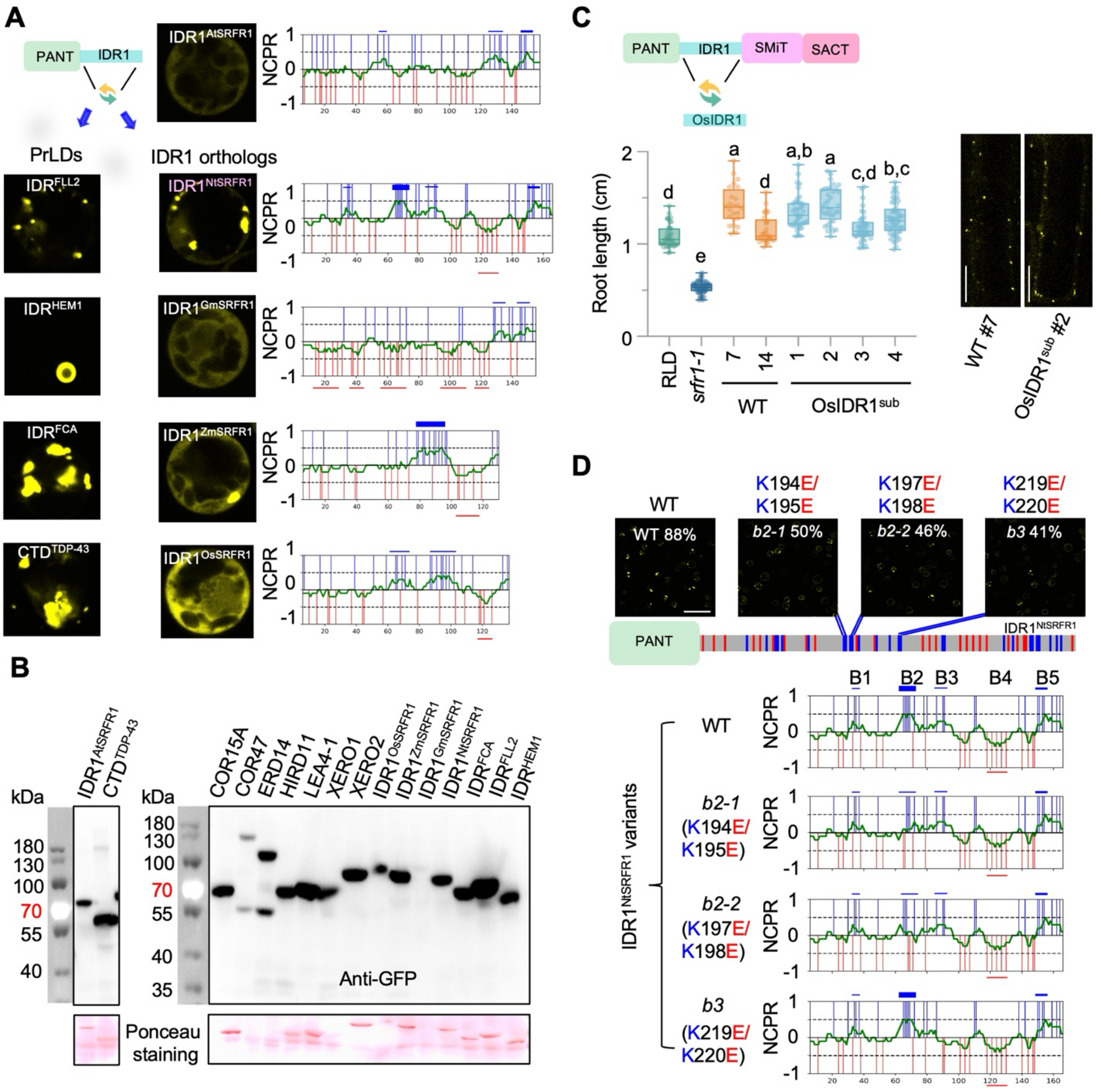

Supplementary Fig. S10. Functional substitution of the IDR1 domain of SRFR1 with its orthologs (supports Fig. 10). **A)** Subcellular localization of the PANT domain fused with IDR1 orthologs and PrLDs. Images were taken using the same confocal microscopy settings. The local NCPR of SRFR1 IDR1 from Arabidopsis, tobacco, soybean, maize

and rice is shown. Positively and negatively charged amino acids are indicated by blue and red vertical lines, respectively. Acidic and basic blocks are represented by red and blue horizontal bars, respectively. Weak blocks are defined as 0.25 < NCPR < 0.5 or -0.5 < NCPR < -0.25; intermediate blocks as 0.5 ≤ NCPR < 0.75 or -0.75 < NCPR ≤ -0.5; and strong blocks as 0.75 ≤ NCPR ≤ 1 or -1 ≤ NCPR ≤ -0.75. **B)** Western-blot detection of YFP-tagged PANT domain fused with IDR1 ortholog and PrLDs expressed in rice protoplasts. Ponceau S staining of the Rubisco large subunit is shown as a protein loading control. The $IDR1^{AtSRFR1}$ and $CTD^{TDP\text{-}43}$ samples in the left panel were run on the same gel as the middle panel of Supplementary Fig. S3E; therefore, the same maker lane was reused. **C)** Primary root length and upper LRC condensate accumulation. Images were taken from the roots of 6-day-old seedlings. n = 24-56. All data points are shown in box-and-whisker plots. Boxes indicate the interquartile range, with the center line denoting the median. Whiskers extend to the minimum and maximum values of the dataset. Letters denote statistically different groups (ANOVA, Tukey-Kramer grouping). Maximal projections of multiple single confocal images are shown. Bar = 10 µm. **D)** Subcellular localization of the PANT domain fused with NtIDR1 and its variants. Images were taken using the same confocal microscopy settings. The local NCPR of SRFR1 IDR1 from Arabidopsis, tobacco, soybean, maize and rice is shown. Positively and negatively charged amino acids are indicated by blue and red vertical lines, respectively. Acidic and basic blocks are represented by red and blue horizontal bars, respectively. Weak blocks are defined as 0.25 < NCPR < 0.5 or -0.5 < NCPR < -0.25; intermediate blocks as 0.5 ≤ NCPR < 0.75 or -0.75 < NCPR ≤ -0.5; and strong blocks as 0.75 ≤ NCPR ≤ 1 or -1 ≤ NCPR ≤ -0.75. The average percentage of protoplasts exhibiting puncta formation is indicated on each representative image. The percentage was determined by counting 3-5 randomly selected low magnification images from large areas, each containing 80-120 protoplasts.

ALT TEXT: This figure demonstrates that IDR1 orthologs, but not PrLDs, can functionally substitute for SRFR1 IDR1 in regulating PANT polymerization. Panel B specifically shows the NCPR graphs and charge blocks of $IDR1^{NtSRFR1}$ and its three variants. The data indicates that shifting basic blocks towards acidic blocks in $IDR1^{NtSRFR1}$ attenuates PANT domain aggregation.

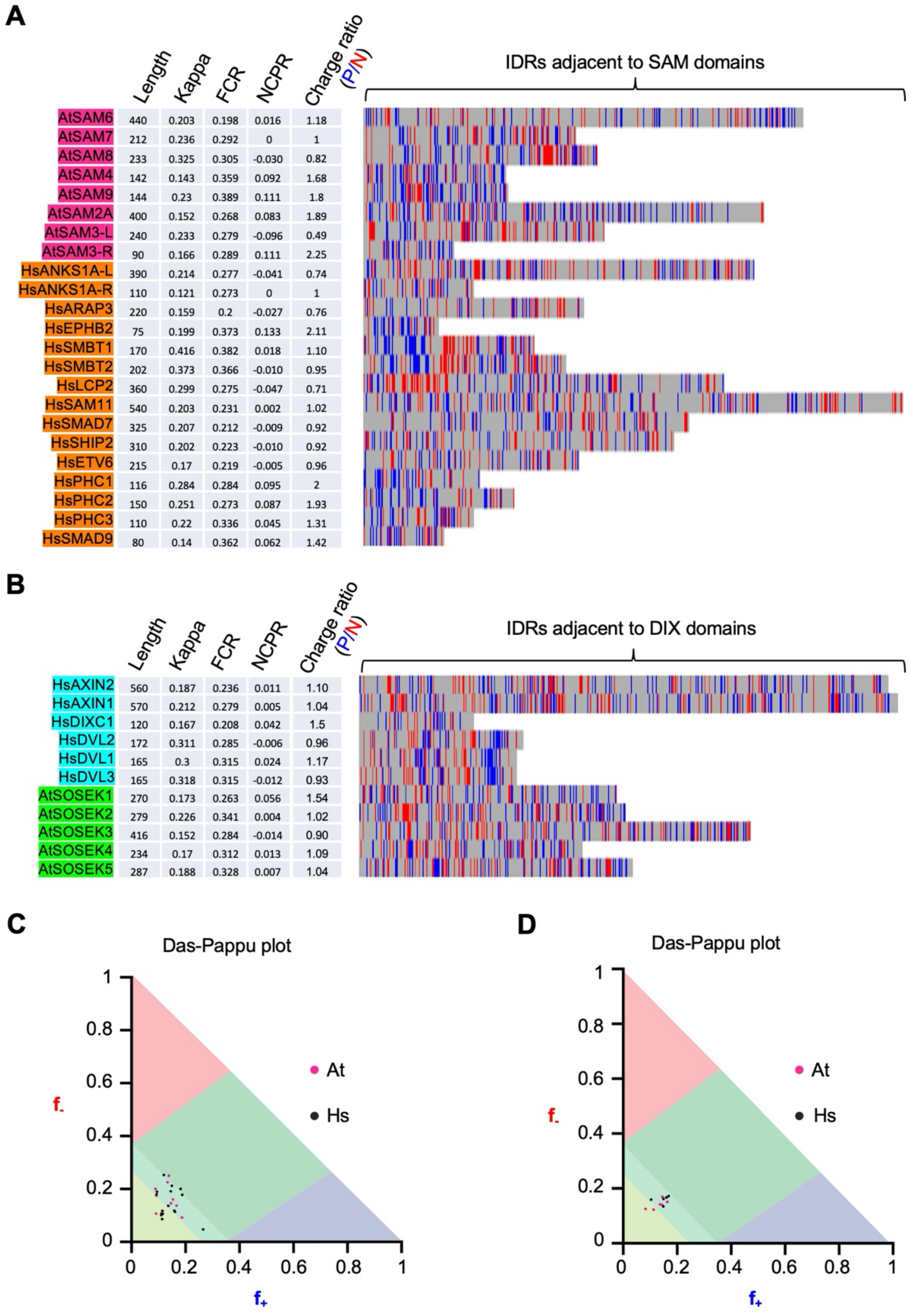

	Length	Kappa	FCR	NCPR	Charge ratio (P/N)
AtSAM6	440	0.203	0.198	0.016	1.18
AtSAM7	212	0.236	0.292	0	1
AtSAM8	233	0.325	0.305	-0.030	0.82
AtSAM4	142	0.143	0.359	0.092	1.68
AtSAM9	144	0.23	0.389	0.111	1.8
AtSAM2A	400	0.152	0.268	0.083	1.89
AtSAM3-L	240	0.233	0.279	-0.096	0.49
AtSAM3-R	90	0.166	0.289	0.111	2.25
HsANKS1A-L	390	0.214	0.277	-0.041	0.74
HsANKS1A-R	110	0.121	0.273	0	1
HsARAP3	220	0.159	0.2	-0.027	0.76
HsEPHB2	75	0.199	0.373	0.133	2.11
HsSMBT1	170	0.416	0.382	0.018	1.10
HsSMBT2	202	0.373	0.366	-0.010	0.95
HsLCP2	360	0.299	0.275	-0.047	0.71
HsSAM11	540	0.203	0.231	0.002	1.02
HsSMAD7	325	0.207	0.212	-0.009	0.92
HsSHIP2	310	0.202	0.223	-0.010	0.92
HsETV6	215	0.17	0.219	-0.005	0.96
HsPHC1	116	0.284	0.284	0.095	2
HsPHC2	150	0.251	0.273	0.087	1.93
HsPHC3	110	0.22	0.336	0.045	1.31
HsSMAD9	80	0.14	0.362	0.062	1.42

	Length	Kappa	FCR	NCPR	Charge ratio (P/N)
HsAXIN2	560	0.187	0.236	0.011	1.10
HsAXIN1	570	0.212	0.279	0.005	1.04
HsDIXC1	120	0.167	0.208	0.042	1.5
HsDVL2	172	0.311	0.285	-0.006	0.96
HsDVL1	165	0.3	0.315	0.024	1.17
HsDVL3	165	0.318	0.315	-0.012	0.93
AtSOSEK1	270	0.173	0.263	0.056	1.54
AtSOSEK2	279	0.226	0.341	0.004	1.02
AtSOSEK3	416	0.152	0.284	-0.014	0.90
AtSOSEK4	234	0.17	0.312	0.013	1.09
AtSOSEK5	287	0.188	0.328	0.007	1.04

Supplementary Fig. S11. Association with zwitterionic IDRs is common among polymerization domains (supports Fig. 9 and Fig. 10). **A** and **B)** Diagram illustrating the arrangement of amino acids in IDRs adjacent to SAM and DIX domains, respectively. Positively charged amino acids are colored in blue, negatively charged amino acids are

colored red, and others are shown in gray. **C** and **D)** The Das-Pappu plot shows the distribution of IDRs adjacent to SAM and DIX domains, respectively.

ALT TEXT: Diagrams show amino acid arrangements in IDRs near SAM and DIX domains, with Das-Pappu plots illustrating their distribution.

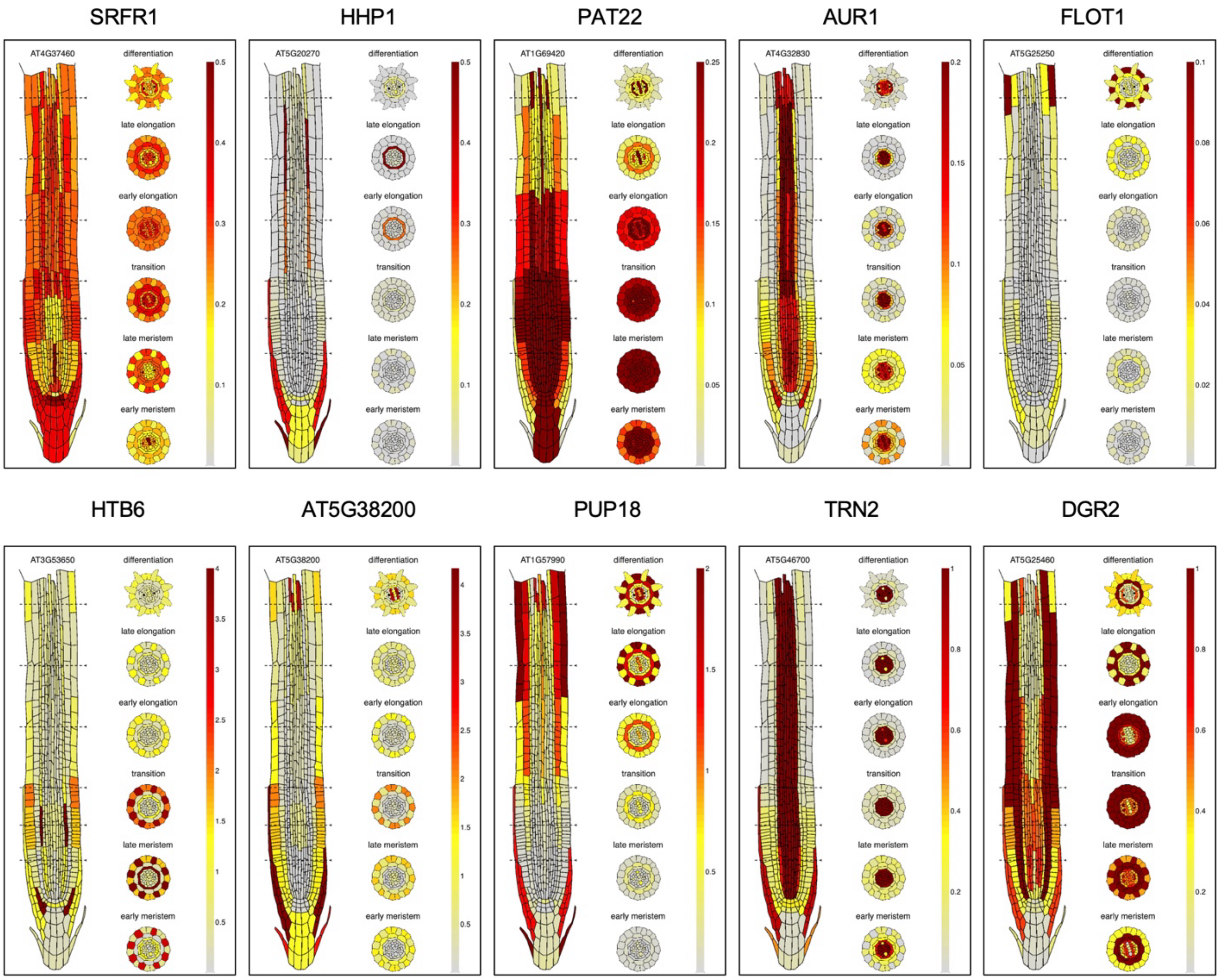

Supplementary Fig. S12. Digital expression patterns of putative SRFR1 condensate components in different root tissues (supports Fig. 12). Integrated single-cell expression data were obtained from the Root Cell Atlas website (https://rootcellatlas.org).

ALT TEXT: Images show tissue-specific gene expression profiles of nine putative SRFR1 condensate components. Expression data were obtained from an integrated single-cell RNA sequencing dataset available on the Arabidopsis Root Cell Atlas website.
