# Supplementary material for: Polymerization-mediated SRFR1 condensation in upper lateral root cap cells regulates root growth
Source: Plant Cell. 2025 Dec 30;38(1):koaf292. doi: 10.1093/plcell/koaf292 (PMC12862871; doi:10.1093/plcell/koaf292)
Supplement: koaf292_Supplementary_Data [file koaf292_supplementary_data.zip › Supplementary Data Set 2.docx]

**Sequences used for charge patterning analysis**

**Prion-like Domains** (Supports Fig. 6F and Supplementary Fig. S8B)

> HEM1_PrLD

TPSTPLSTASPYHSPSVSLIHASPSMKNSTTPQRGSGSGSSSTAAPDSGYFKGSSSSLYGQEHYTESETGNSRNNENNNNNKQRGSSRRSGPLDYSSSHKGGSGSNSTGPSPLPRFAVSRSGPISYKQHN

> FCA_PrLD

QHQEKPTIQQSQTQLQPLQQQPQQVQQQYQGQQLQQPFYSSLYPTPGASHNTQYPSLPVGQNSQFPMSGIGQNAQDYARTHIPVGAASMNDISRTQQSRQSPQELMWKNKA

> FLL2_PrLD

NSDRRAGGPYGNNINAEIDASGHQSGNGYYEDAFGPQGYIPQPVAGNATGPNSVVGAAQYPYQGVTQPGYFPQRPGYNFPRGPPGSYDPTTRLPTGPYGAPFPPGPSNNTPYAGTHGNPSRR

> TDP-43_PrLD

EPKHNSNRQLERSGRFGGNPGGFGNQGGFGNSRGGGAGLGNNQGSNMGGGMNFGAFSINPAMMAAAQAALQSSWGMMGMLASQQNQSGPSGNNQNQGNMQREPNQAFGSGNNSYSGSNSGAAIGWGSASNAGSGSGFNGGFGSSMDSKSSGWGM

> FUS_PrLD

MASNDYTQQATQSYGAYPTQPGQGYSQQSSQPYGQQSYSGYSQSTDTSGYGQSSYSSYGQSQNTGYGTQSTPQGYGSTGGYGSSQSSQSSYGQQSSYPGYGQQPAPSSTSGSYGSSSQSSSYGQPQSGSYSQQPSYGGQQQSYGQQQSYNPPQGYGQQNQYNSSSGGGGGGGGGGNYGQDQSSMSSGGGSGGGYGNQDQSGGGGSGGYGQQ

**SRFR1 IDR1 orthologs** (Supports Fig. 6F and Supplementary Fig. S8B)

>AtSRFR1_F4JS25_135-296

ATESPQETPAYHSEKSDEKSDKLDNHESGASSNGNSHESSSELGEQSKIVSFSKVASKASKQSDGNSDLCNGSVYKEKENGKCGSQINGYYESCKPCNGSDLHDNLAESSDRFGELSINGNKISIKSSKMSHKAEARCGISDESRKNKKYTIARISGTHSIS

>AlSRFR1_D7MAM0_129-286

SRQETPASHSEKSDEKTDKLDNHESGACSNGNSHDHEASRELGEKSKIISFSKDTSKASKQSDGSSDLCNGSAYKEKENGKCGSQINGSYESCKPCNGSDLHDNLAESSDRLGDLSINGNKLIIKSSKMSHKAEARCDETRKNKKYTIARISGTHSI

>CrSRFR1_R0F3L6_130-319

TFKNHASEPRQETSASPQSEKSDEKIDKLDNQNTASGTSINACKTTSISVSESGACSNGNSDEASRELGEQSKINSISKDVSKASKQSRGSSDLCNGSSFKEKENGKCGSQINGSYETCKPCNGSDLHDNLAESSDWLGNVTINGKKLSITSGFTSKSSHKAEVRCGVGNETTINKKYTIARISGNHSIS

>LsSRFR1_A0A2J6LZ59_131-255

LSEWSVSKPEPNNSVRTSETSNNHTMSSIGYEPIGDSREIESKQNHVSSNGNHEKQQNGTYNISVDFGARSSTSDTRKKSDLSTKISLVPSKSSDNDKSDMNSEQSNDAKRNKKFSVTRISKAKS

>SlSRFR1_A0A3Q7FBA4_126-297

AVGSNNHSVQSSGPESNTGPPLSTKSGETCDISKASDRELKTCSSGMLESSEKSKNSSVLQNSSSNNSKKHKKIESESKELHERQANKTNNNCKKLGYPSLVCSELSDISEDSRKSSAVTSESSEQSEPNELQEILSQLNNKCDVRVELSDEGKRNKKFCVTRVNKTKSINV

>NtSRFR1_A0A1S3ZZ32_129-299

DNHSVESSGPESNTGPMLSTKSAETCDISKASDRKLKECSSGMLVSCEKSNDSSVLQNSSSNNAKKHKKIDRQPNGLHERQANGTKNNCKKLGYPSLVCSELSDVSEGSRKSSAVTSESSEQSEPNELQEILCQLNNKCDVRLELTEEGKRNKKFCVTRINKTKSINVDF

>GrSRFR1_A0A0D2PDD9_122-347

KPGKQDISFISDNHVADSKLSTPVSLSTPYVDGKLNETLKYQNEYNTSRLFQERRDVSKFCNMSNDKIDPRNRTNDEERSQSSLSSSELASDTNEKSRESFKNLTVLSDGSKLSVESADASENSSICGDNCNGGLSDLTSTNQMPHGLTNGTHNNFDTPSNSSDSGTALSEKSEPCSKSSAISSNSSDITEGHCLPNNSSGSHNEISDEAKRSKKFCVAKISKTKS

>CcSRFR1_V4SFD3_135-346

NSMSSLTVSEPGLNANDKMSETSENHNKSDICDSSSQSRDVSETCSKSSHDPDLCNGRSDEAKGGSSVPVSKSGLHINGKLREVSENHNGSSDGSKSTHASRDASEINRQSSDDFDICNGPIDKASVNERHGRQTNGTHDVHDKLSSDSASLNDSNTNSESYSKSSISDNKSSDSTESRSKLSFKWDMLKETSNEARRNKKFCVTRISKSKS

>EgSRFR1_A0A059AM61_133-289

ADSTSTVSVLDSVTQVNGESNEISRNQKSLRDALKNSSRSNGTSDVPYKPNRSLDLPSCFSSEAKRKEKLECKINGSLHVLDDSKSESKSCETSLTGSESSIICAKPSDMAETSSKLNDKVVAQSEMSDGVTHNEITDDPRKNKKACVTKLSKNRSI

>JcSRFR1_A0A067KJM3_131-338

NNVTESRSSMNVTESSKIQNKSSDFSSSSGESGDASQSCSKFRDKFEVLNGIKDEAGGKSPIPIPESGSFVNGKPTENYINQNRLGDKHYLCSESRDTSEFYCKSGNNFGMQNDLSEKAEGGKKVDSPMNVTHDILDKPSHSSNSYNSLSNTSEFPSKFSKLPSSLGETSHIRSKSSNETDVPNEASDEANKSKKFCVTKISKTKSVT

>GmSRFR1_K7K4N6 _127-285

ALYETNGSPVSQSESDSPSDGNLTEICENQDRLSVQDELCDNASDKSLILLKSADNFDLRNELNIEDRESNKSDSQVNGSPDVIDKLSYNSESCNDSSDTSESCDKDKVFTNSGESSSDSLDVAEILRKLSSKFNFPHEKIGEARKNKFCVARISKTKS

>CsSRFR1_A0A0A0KIG1_119-276

NGEVESGFSTVGPESGVTSASKNCRETNNNVGKLMEHPDFCMKSSDSSEVCSISSDNLVVCEGGCEEVGPNRDIKCESNGCTDNDDRLSDASKLHDLRSDMPEFCNKSKLTAFHSKSGDSTDILSKSSKTSDVRGNVSDEARRTKKFSVAKISKTKSI

>MdSRFR1_A0A498I0L0_127-285

NGDDNQAKESASSKSSDNDKSDMNSEQSNDAKLVSEASPHINGKSGETYKNHNKLSGESKLCCESTVTSAVHGKFNGNGNFVSSKGIGDKAGGSKKFDSQMNGNHDINDKLHSESCNDLSDTCSKLPMICSKSSDLTETPPTPPKLSTKSDIRHEIGEESKRNKKFSVARLSKTKSI

>VvSRFR1_F6HSV4_129-315

HAMESPESSISVSESALHVNEKPNSTHKNDSKLNDESELCSESSDTSEIHCKPNSTHKNDSKLNDESELCSESSDTSEIHCKAFDTSDGHDELRDTVNGNEKLNSESNGTYDIFVKSSDESELCSELNDTSEQSSKSSVVIHSKSSDISEVRRKSSNKYDIRSELGDEANRNKKFCVTRISKTKSIS

>NnSRFR1_ A0A1U8B6B5_135-227

ESSNQTVPLSDSGLQKFSENNKSSDTSELSRKLNDTHEMYNKSSETSEIHTKSSDTNEIHKKSRDAPEQRCKLSEEPKRNSKIFVTGIPKTKS

>AcSRFR1_ A0A2G5CP57_129-267

HAMDSSTPIMSLRDSGTKTHTKANGTVEISSKSCEASQLCNNSSVTSETSSKPKGTSESESKSSETSQVCNNSSVTSEASSKPKGTSESESNSTSKSEVHKRSSDTSEFCIKLSDEVEIRTRPSDGSKRSLKILGNGNS

>OsSRFR1_ Q7XHN9_129-262

DRVVDASPCDTKVVISEDRVVDTSCTATTMADTKTVVCEENIGNSGVISNGAVILANDNKADNNKECSSPTKDTTGTHHTPKKTTKPDKKSKAKGRKEINSQIEDVADSISSGETVAVDQTLFASKISKSSKSI

>TaSRFR1_ A0A453BPP7_121-258

IYETVECEDVVDASPCDTKVVISEDRFINTSFTAATTDTKTVVCEENSANSKSSSNSDALLPNNDKDHKDSVSSAKDTTVPHQTPKKQPKTDKKNKAKVKKETNGQAEDVEERRSSDETITLDQALFATKVSKSSKSI

>SbSRFR1_ A0A1B6Q8L6_126-264

ESADHVMDSSTCDTKVVISEDRVADKSLAATTMADTKTVVCEEAIGSSKVSSNGDTKLTNYNKKTDHNKVSSSPVKDTTGTQAPKKAPKSDKKNKAKAVKEINGRAEGVTVRTSADESETILLDQTLFATKISKASKSI

>ZmSRFR1_ A0A1D6FA37_124-264

TTQSADHVMDASTCDTKVVISEDRVADTSFAATTMADTKTVVCEEAIGSSKVSSNGDTKLTNYNKKADHNKVSSSSVKDITGTQAPKKAPKLDKKNKAKAVKEINGRAEGVTGRASTDESETISLDQALFATKISKSSKSI

>MaSRFR1_ A0A804IZN6_123-265

SPSFAGEDHSMDASSCETKVVVSEDHIMDSSSTNVSTTETEAIICEDHGSSGVTKVVVSEDHVTDSRIKNSSTTNAEHTTNSSGVTPSDKESVNGHKLENTHEICSKPVDSTDIFSMYNDTTKLGRKVVVTGIQKTKSISLD

>PpSRFR1_ A0A2K1L4F1_152-211

LSKASSENEETTSNIVKAASLDSSTRNSKVGAVTVTGSASEPMEHIYGVSKSRQNRLSVT

>CrSRFR1.1_ A0A2K3E698_119-366

DPTGVKVGLDGPTSRSPAQEQNGVGGRAVLGLQVVSILLLAVLVAGQYQLLSHKAGLSAVVGPLSADVAKAAAPAAASTSSWAPATPNPSPSSNGGAAPAPAAPAPSSSSSATTSPADAAGPSKPAAPAAEPAAQQKAGAATAAPSTGRGGVASTSAPSSSSTSSSSSSSSSNGGAASKPSSKSASPPPPGPSKGPAAAASTASSSSSSKAAAAGAVGECDVEEVDCVAQQKAARAAAAGGPGGAASP

>CrSRFR1.2_ A0A2K3E656_121-319

TGVKAGLSAVVGPLSADVAKAAAPAAASTSSWAPATPNPSPSSNGGAAPAPAAPAPSSSSSATTSPADAAGPSKPAAPAAEPAAQQKAGAATAAPSTGRGGVASTSAPSSSSTSSSSSSSSSNGGAASKPSSKSASPPPPGPSKGPAAAASTASSSSSSKAAAAGAVGECDVEEVDCVAQQKAARAAAAGGPGGAASPD

**LEA proteins** (Supports Fig. 7A)

> AT5G06760

MQSMKETASNIAASAKSGMDKTKATLEEKAEKMKTRDPVQKQMATQVKEDKINQAEMQKRETRQHNAAMKEAAGAGTGLGLGTATHSTTGQVGHGTGTHQMSALPGHGTGQLTDRVVEGTAVTDPIGRNTGTGRTTAHNTHVGGGGATGYGTGGGYTG

> AT1G32560

MQSAKQKISDMASTAKEKMVICQAKADEKAERAMARTKEEKEIAHQRRKAKEAEANMDMHMAKAAHAEDKLMAKQSHYHVTDHGPHVPQQAPVPAPAPVMGHGYGHNPTGVTSVPPQTYHPTYPPTGHHNHHHY

> AT2G35300

MQSAKEKISDMASTAKEKLNIGGAKAQGHAEKTMARTKKEKKLAQEREKSKEAQAKADLHQSKAEHAADAQVHGHHLPGHSTYPTRATGANYPPGQI

> AT1G20450_ERD10

MAEEYKNTVPEQETPKVATEESSAPEIKERGMFDFLKKKEEVKPQETTTLASEFEHKTQISEPESFVAKHEEEEHKPTLLEQLHQKHEEEEENKPSLLDKLHRSNSSSSSSSDEEGEDGEKKKKEKKKKIVEGDHVKTVEEENQGVMDRIKEKFPLGEKPGGDDVPVVTTMPAPHSVEDHKPEEEEKKGFMDKIKEKLPGHSKKPEDSQVVNTTPLVETATPIADIPEEKKGFMDKIKEKLPGYHAKTTGEEEKKEKVSD

> AT4G38410

MADHPRSTEQQEADAAASKGCGMFDFLKKKPEDVHSSENARVTKEPKEEEKPSLAERFHLSDSSSSDEEAGENGEKKEKKKKKKKNEVAEDQCETEEKIPAGIGHEDGKEKGFMEKIKDKLPGGHNGKPEAEPHNDKAKEKGFMEKIKEKLPGHTNDEKKKET

> AT1G20440_COR47

MAEEYKNNVPEHETPTVATEESPATTTEVTDRGLFDFLGKKEEEVKPQETTTLESEFDHKAQISEPELAAEHEEVKENKITLLEELQEKTEEDEENKPSVIEKLHRSNSSSSSSSDEEGEEKKEKKKKIVEGEEDKKGLVEKIKEKLPGHHDKTAEDDVPVSTTIPVPVSESVVEHDHPEEEKKGLVEKIKEKLPGHHDEKAEDSPAVTSTPLVVTEHPVEPTTELPVEHPEEKKGILEKIKEKLPGYHAKTTEEEVKKEKESDD

> AT1G76180_ERD14

MAEEIKNVPEQEVPKVATEESSAEVTDRGLFDFLGKKKDETKPEETPIASEFEQKVHISEPEPEVKHESLLEKLHRSDSSSSSSSEEEGSDGEKRKKKKEKKKPTTEVEVKEEEKKGFMEKLKEKLPGHKKPEDGSAVAAAPVVVPPPVEEAHPVEKKGILEKIKEKLPGYHPKTTVEEEKKDKE

> AT1G54410_HIRD11

MAGLINKIGDALHIGGGNKEGEHKKEEEHKKHVDEHKSGEHKEGIVDKIKDKIHGGEGKSHDGEGKSHDGEKKKKKDKKEKKHHDDGHHSSSSDSDSD

> AT2G21490

MADLRDEKGNPIHLTDTQGNPIVDLTDEHGNPMYLTGVVSSTPQHKESTTSDIAEHPTSTVGETHPAAAPAGAGAATAATATGVSAGTGATTTGQQHHGSLEEHLRRSGSSSSSSSEDDGQGGRRKKSIKEKIKEKFGSGKHKDEQTPATATTTGPATTDQPHEKKGILEKIKDKLPGHHNHNHP

> AT5G66400_RAB18

MASYQNRPGGQATDEYGNPIQQQYDEYGNPMGGGGYGTGGGGGATGGQGYGTGGQGYGSGGQGYGTGGQGYGTGTGTEGFGTGGGARHHGQEQLHKESGGGLGGMLHRSGSGSSSSSEDDGQGGRRKKGITQKIKEKLPGHHDQSGQAQAMGGMGSGYDAGGYGGEHHEKKGMMDKIKEKLPGGGR

> AT3G50980_XERO1

MESYQNQSGAQQTHQQLDQFGNPFPATTGAYGTAGGAPAVAEGGGLSGMLHRSGSSSSSSSEDDGLGGRRRKKKGITEKIKEKLPGHHDSNKTSSLGSTTTAYDTGTVHHEKKGMMEKIKEKLPGGHH

> AT3G50980_XERO2

MNSHQNQTGVQKKGITEKIMEKLPGHHGPTNTGVVHHEKKGMTEKVMEQLPGHHGATGTGGVHHEKKGMTEKVMEQLPGHHGSHQTGTNTTYGTTNTGGVHHEKKSVTEKVMEKLPGHHGSHQTGTNTAYGTNTNVVHHEKKGIAEKIKEQLPGHHGTHKTGTTTSYGNTGVVHHENKSTMDKIKEKLPGGHH

> AT2G33690

MSGNMSKSEEKQELPLETSPYTKYEDIEDYKKNAYGTSGHQDVKPGHGGGTTDAPTPSGDAAPSAIDSANQKAKK

> AT2G23110

MEDQKKPPTTEQEVKEVKNDDLESIKTPYLDYDNLEDYKMKGYGAQGHQEPKLGMGGGATDAPTPSGGLGRGGGAASTDLSSTDAINRQGVP

> AT2G23120

MEAGKTPPTTTTTTEKKTEQVKDNDLPTDSPYMATGTLEDHKLKAYGAEGHQEPTPGLGGGSTDAPTPSGDAPAATTTDAKAP

> AT2G44060

MSTSEDKPEIISRVVHQEGDVEIVDRSQKDKDEEKEEGKGGFLDKVKDFIHDIGEKLEGTIGFGKPTADVSAIHIPKINLERADIVVDVLVKNPNPVPIPLIDVNYLVESDGRKLVSGLIPDAGTLKAHGEETVKIPLTLIYDDIKSTYNDINPGMIIPYRIKVDLIVDVPVLGRLTLPLEKCGEIPIPKKPDVDIEKIKFQKFSLEETVAILHVRLQNMNDFDLGLNDLDCEVWLCDVSIGKAEIADSIKLDKNGSGLINVPMTFRPKDFGSALWDMIRGKGTGYTIKGNIDVDTPFGAMKLPIIKEGGETRLKKEDDDDDDEE

> AT1G01470

MASLLDKAKDFVADKLTAIPKPEGSVTDVDLKDVNRDSVEYLAKVSVTNPYSHSIPICEISFTFHSAGREIGKGKIPDPGSLKAKDMTALDIPVVVPYSILFNLARDVGVDWDIDYELQIGLTIDLPVVGEFTIPISSKGEIKLPTFKDFF

> AT2G46140

MASADEKVVEEKASVISSLLDKAKGFFAEKLANIPTPEATVDDVDFKGVTRDGVDYHAKVSVKNPYSQSIPICQISYILKSATRTIASGTIPDPGSLVGSGTTVLDVPVKVAYSIAVSLMKDMCTDWDIDYQLDIGLTFDIPVVGDITIPVSTQGEIKLPSLRDFF

> AT1G02820

MARSLANAKIQSVFGSEKLSNAVFRRGFAAAAKTALDGSVSTAEMKKRAGEASSEKAPWVPDPKTGYYRPETVSEEIDPAELRAILLNNKQ

> AT4G02380

MARSISNVKIVSAFVSRELSNAIFRRGYAATAAQGSVSSGGRSGAVASAVMKKKGVEESTQKISWVPDPKTGYYRPETGSNEIDAAELRAALLNNKQ

> AT4G15910

MAARSLSGAVKSLCSAASGSLSCSIVLRRSYVATSQNVTAAGLSKGGSTRVMVGKMEQRGLDQEAESAWGPDPVTGYYRPSNRAAEIDPAELRELLLKNKAKSF

> AT3G53770

MSQSLFNLKSLSRSINNTIRMRRYIVITKASQRAYTIGSSQEKPSWASDPDTGYFRPETAAKELDPYIAKTSQVQGKMMRGEELWWMPDPQTGYYRPDNFARELDAVELRSLHFNKNQKTYVVS

> AT4G39130

MADLKDERGNPIYLTDAHGEPAQLMDEFGNAMHLTGVATTVPHLKESSYTGPHPITAPVTTTNTPHHAQPISVSHDPLQDHDLRWFGTSSTEENGEGVGRKTNITDETKSKLGVDKPSAATVTGSGSGSVHEKKGFFKKIKEKLSGHHNDL

> AT4G13560

MSQQQFNAGQNRGQAQEKAEQWTESAKQTAQSACDKTADLTQSARDKAADLTQSARDKTADGSHSANKSAQHNQEQAAGLFGQTGESVKNMAQGALDGVKNSLGMNEKK

> AT1G52690

MASHQEQSYKAGETRGKAQEKTGEAMGTMGDKTQAAKDKTQETAQSAQQKAHETAQSAKDKTSQAAQTTQERAQESKDKTGSYMSETGEAIKNKAHDAAEYTKETAEAGKEKTSGILGQTGEQVKQMAMGATDAVKHTLGLRTDEGNKEHVSSAPSTTTTTTTRETQRK

> AT3G15670

MASNQQSYKAGETRGKAQEKTGQAMGTMRDKAEEGRDKTSQTAQTAQQKAHETAQSAKDKTSQTAQAAQQKAHETAQSAKEKTSQTAQTAQQKAHETTQAAKEKTSQAGDKAREAKDKAGSYLSETGEAIKNKAQDAAQYTKETAQGAAQYTKETAEAGRDKTGGFLSQTGEHVKQMAMGAADAVKHTFGMATEEEDKEHYPGSTTTTTATTRTTDPTHQTYQRK

> AT3G02480

MDNKQNASYQAGQATGQTKEKAGGMMDKAKDAAASAQDSLQQTGQQMKEKAQGAADVVKDKTGMNKSH

> AT4G13230

MTSFAVVARLITRAPRVRASVPTRLVHGTTSTRKDSVCDKATEAQQKVAKKADEGAQTISDAAGNLKDKAKNTAEEAWDKVKDTTEKIKDTVTGKTEETKESIKATAKTVERSMNTKNLK

> AT5G44310

MAAMQLTRTVFFGISKAFPKSQAPRTLAVAIGRKSSRVFFASSVNHSKGRYDPVEKARDSRADLAYDSKKWREESGEYAEAGKGKAHKTKEEAKDKAYDMKERTKDYAEQTKNKVNEGASRAADKAYETKEKAKDKAYDVKEKTKDYAEEAKDKVNEGASRAADKAYETKEKAKDKAYDVKEKTKDFAEETKEKVNEGASRAADKAYETKEKAKDKAYDVKEKTKNYAEQTKDKVNEGASRAADKAEETKDKAKDYAEDSKEKAEDMAHGFKEKAQDIGEKTMDTVKDVWETAKSTAQKVTEAVVGSGEEADKARDDVDKGLEDLSKKAKENRNKDDDFKRF

> AT4G21020

MAAMQLTRTALVGLSKAFPGIKAPATLAASSRKVSRICFATSVSQNEGRDPLDNARDSRADSAYGSKKWREDTGEYYAQAAKDKANEGASKAADKAYETKEQAKDKAYETKEKAKDTAYNAKEKAKDYAERTKDKVNEGAYKAADKAEDTKEKAKDYAEDTMDNAKEKARHAKEKVKEYGEDTKEKAEGFKETVKGKAEELGEKTKETVKGAWESTKNAAQTVTEAVVGPEEDAEKARADMNKGVEDHRKKKAEKDQKEDDFITFN

> AT3G53040

MASGQREAERSAKAERAEAAASLAAEDLKDINKGDVTYKLTERTTTTEPERPGLIGSVMKAVQGTKDAVIGKSHDTAESTREGADIASEKAAGMRDTTGEVRDSTAQKTKETADYTADKAREAKDKTADKTKETADYAAEKAREAKDRTADKTKETAEYTAEKAREAKDKTADKLGEYKDYTAEKAKEAKDTTAEKLGEYKDYTVDKAKEAKDKTAEKAKETAEYTSDKARETKDKTAEKVGEYKDYTAEKAKETADKAREAKDKTAEKVGEYRDYTAEKATETKDAGVSKIGELKDSAVDTAKRAMGFLSGKTEETKQKAVETKDTAKEKMDEAGEEARRKMEEMRLEGKKLDEDASRKTQQSTESAADKAHETKDSVAQRGEEGKGSIMGALGNMTGAIKSKLTGATTPSDEETRASAHGDESTGKTVVAVDVKDTRPGYVATVLKEADQMTGQTFNDVGEIDDEEKVRIVVGEKKL

> AT2G36640

MASDKQKAERAEVAARLAAEDLHDINKSGGADVTMYKVTERTTEHPPEQDRPGVIGSVFRAVQGTYEHARDAVVGKTHEAAESTKEGAQIASEKAVGAKDATVEKAKETADYTAEKVGEYKDYTVDKAKEAKDTTAEKAKETANYTADKAVEAKDKTAEKIGEYKDYAVDKAVEAKDKTAEKAKETANYTADKAKEAKDKTAEKVGEYKDYTVDKAVEARDYTAEKAIEAKDKTAEKTGEYKDYTVEKATEGKDVTVSKLGELKDSAVETAKRAMGFLSGKTEEAKGKAVETKDTAKENMEKAGEVTRQKMEEMRLEGKELKEEAGAKAQEASQKTRESTESGAQKAEETKDSAAVRGNEAKGTIFGALGNVTEAIKSKLTMPSDIVEETRAAREHGGTGRTVVEVKVEDSKPGKVATSLKASDQMTGQTFNDVGRMDDDARKDKGKL

> AT2G42560

MASEQARRENKVTEREVQVEKDRVPKMTSHFESMAEKGKDSDTHRHQTEGGGTQFVSLSDKGSNMPVSDEGEGETKMKRTQMPHSVGKFVTSSDSGTGKKKDEKEEHEKASLEDIHGYRANAQQKSMDSIKAAEERYNKAKESLSHSGQEARGGRGEEMVGKGRDSGVRVSHVGAVGGGGGGEEKESGVHGFHGEKARHAELLAAGGEEMREREGKESAGGVGGRSVKDTVAEKGQQAKESVGEGAQKAGSATSEKAQRASEYATEKGKEAGNMTAEQAARAKDYALQKAVEAKETAAEKAQRASEYMKETGSTAAEQAARAKDYTLQKAVEAKDVAAEKAQRASEYMTETGKQAGNVAAQKGQEAASMTAKAKDYTVQKAGEAAGYIKETTVEGGKGAAHYAGVAAEKAAAVGWTAAHFTTEKVVQGTKAVAGTVEGAVGYAGHKAVEVGSKAVDLTKEKAAVAADTVVGYTARKKEEAQHRDQEMHQGGEEEKQPGFVSGARRDFGEEYGEERGSEKDVYGYGAKGIPGEGRGDVGEAEYGRGSEKDVFGYGPKGTVEEARRDVGEEYGGGRGSERYVEEEGVGAGGVLGAIGETIAEIAQTTKNIVIGDAPVRTHEHGTTDPDYMRREHGQR

> AT2G42540

MAMSFSGAVLTGMASSFHSGAKQSSFGAVRVGQKTQFVVVSQRKKSLIYAAKGDGNILDDLNEATKKASDFVTDKTKEALADGEKAKDYVVEKNSETADTLGKEAEKAAAYVEEKGKEAANKAAEFAEGKAGEAKDATK

> AT2G42530

MAMSLSGAVLSGMGSSFHNVGAKQSGVGTVRVGRKSELVVVAQRKKSLIYAVKSDGNILDDLNEATKKASDFVTDKTKEALADGEKTKDYIVEKTIEANETATEEAKKALDYVTEKGKEAGNKAAEFVEGKAEEAKNATKS

> AT2G03740

MSISGAVLSGLGPSFLISGGKRSGVGGGAMKVGRKNVIIAPQRKKSWVSAAVKGAGNSPNDPKWLDDASEKASGYVKEKGSEVGNVSAQKGQELQNQMERAKDYIFGKAGEAMDSVAENAKRASDFVTEKGKEVKEETTSRTDKAKDFIVEKAGDVKDTAMDMRNKTSKYVGDKATEAKEAILPPKTDA

> AT2G03850

MAMSISGAVFNGLGSSFLISRGKRSGVGLGVVAMRVGRKNVITSPKCKKSWVPTAVKGDGNSKLDPKWLDDASQKASDYVKEKGSEVGHASAQKGQDVNDHVDRAKYYMFEKASEAIDNVAEIAQFASEFVTEKGKETKKETASISEKAKDFIVEKAGEIIDIATDVSKKTAKYVGDKAKEVKEAIMPPKT

> AT3G17520

MGLERKVYGLVMVSLVLMAIATMCCVQATIEEEAAKDESWTDWAKEKIGLKHEDNIQPTHTTTTVQDDAWRASQKAEDAKEAAKRKAEEAVGAAKEKAGSAYETAKSKVEEGLASVKDKASQSYDSAGQVKDDVSHKSKQVKDSLSGDENDESWTGWAKEKIGIKNEDINSPNLGETVSEKAKEAKEAAKRKAGDAKEKLAETVETAKEKASDMTSAAKEKAEKLKEEAERESKSAKEKIKESYETAKSKADETLESAKDKASQSYDSAARKSEEAKDTVSHKSKRVKESLTDDDAEL

> AT4G36600

MMIMMLTTVVTLTWQKQCYGWGTETAEDMVRNEAEHAKNAAETAKKMASDAAHDTKDKTASWAGWVSDKISTGLGGKKAEAEEAAESAKNYAYDKAGSAYDNAGYAKDFASDKAGSAYDSAHNAKHYAYDKAGDAKDMAYDKTGQAKYMAYDKAGSAYEKAGQAKDMAYDKAGQAKDMAYDKVGSAYDKAGQAKDMAYDKAGSASEKAGQAKDFAYDKAAHAKDAAYNKAEDVIKMATDTSGEAKDSAYGTYERFKEGSKNAKDIASDKAHDVRETAGRAVDYAKDKANDAYESGSEAAGRFDEAMHKVGERYGAAKDSMSENTKEAYESAKEKASDAAGEYGSYMRDRSAEL

> AT2G18340

MVRVDGEGKDRGFGTKKEETGIYQKSKDEARKAAQAAENYAYDKANYVKDSAYDNAGYAKDFAENKAEYAKDFAYDKAGDAKNMAYENAGYAKDFAYDKAGDAKDMAYEKAGHAKDFAYDKAGNAKDMAYETAGYAKDFAYDKGGYAKDVAYDKAGNAKDMAYEKAGNAKDMAYEKAEHIKDFTYDKVGSAYGSAQSMMDSGYDKAGDAKDMAYEKAGIVKDMAYDKAGDAKDVAYEKAGIAKDMAYDKAGNAKDMAYDKVGSAYGSAQKAKDSGYEKAGEAKDYAYKKAGNAKDIAYEKAQDAKDFAYDKAGYGYDKAGDVIRMATDKSGEAYEGAKEKSKSAKDTAGEAMDDSIDYMKDKSHNAKDGATRGFEEAMEKVGEKYGVAKESTKYAYETAKKKASQVAGEIRDRYAEL

> AT3G51810

MARSLANAKIQSVFGSEKLSNAVFRRGFAAAAKTALDGSVSTAEMKKRAGEASSEKAPWVPDPKTGYYRPETVSEEIDPAELRAILLNNKQ

> AT2G40170

MASQQEKKQLDERAKKGETVVPGGTGGKSFEAQQHLAEGRSRGGQTRKEQLGTEGYQQMGRKGGLSTGDKPGGEHAEEEGVEIDESKFRTKT

> AT2G41260

MGNLKSLVLLALLFSFSVAVFANTSNDATHDEVKPSTEATHAIEAQKHDGKPQIAEAQVEANDPVVEPQQDWGGRGGCRWGCCGGWWRGRCRYCCRSQAEASEVVETVEPNDVEPQQGGRGGGGGGGGGRGGCRWGCCGGWWRGRCRYCCRSQAEASEVVETVEPNDVEPQQGGRGGGGGGGGGRGGCRWGCCGGWWRGRCRYCCRSQAEASEVVETVEPNDVEPQQGGRGGGGGGGGGRGGCRWGCCGGWWRGRCRYCCRSQAEANEVVETVEAQQAKP

> AT2G41280

MGNLMSLVLVALLFSLSLAVIADTSNDATHVKEEVKPSTEATDAIEAEVEVNDAVVEPQQGLPGGGCRFGCCGGYWWNGLCIYCCRSQAEANEVVKTVEPQKEEAKP

**IDRs adjacent to SAM domain** (Supports Supplementary Fig. S10A)

> AtSAM6_aIDR

MAKLRPRQLELSKTVPSKLGFDGEEDAWVFVKKQKIFIVLPSLPLPQQQHFTLEKPAISPQLEAGFRESMEVTQDSTFVHTVVPSLPLPETPQSQAELRDALADTHFTTPVPTVVVPALPLPEQFILHKPETSHSQVQFRDCIANTHKTTPLHTVVHSLPVTEHSTLQKPTSSQSQVELRESIADTHGTTPLQTEISSLPLPQHFVLQKPATSQSQADTHETTLLHTILPSLPVPELCTLQKPATSQSQAELRANTRKATLVHAAAMPSLPVPEHYSLQKPSTSQSQAELRAKTRKATLVHAAAIPSLPVPEHYSLLKPSTSQSQAEVRTLTHKATHVHTAMPSLSVTEHCTLQKPATSQSQAETSRVQTVEPEACPDFTSVDKPEIVMSRSLTTRKAPAPKRSLQESRKNQDRRVEIHRRRAGHKPIRFPRVMCSSVVM

> AtSAM7_aIDR

MAELQPVDGQQTNGGVVTIPASTSAEATAIAAAGSKRLRRPSVRLGEIGGDQYQQQHHHHAAAAAYDSQGRKSKWTPTTTSGNRKDTSKSSRTRTLTNLSSGYENIGTLDDEREGNVDSFGVGSWRVKKRVGSSAAAKRVRSNWVSKVGDGDEKISGGEELEGGFRDFSREDSESPIKEESLDRDGGGFYGRRRYESNNSSGNREFESNMDG

> AtSAM8_aIDR

MAELQLVEGHQINGGFIPPAIINSIEAPETSAAAGVSVGSKRLRRPSVRLGDIGGDQYHQHVVAAYDSPQVRRPKWRPSGGGGGGGGNRKEPNNQSGKTTSSSRTRTMTNLSSGGYENTGTLDEDPVSIGSWRVKKWVKSSGGETAATTTTNTASAKRVRSNWATRNDGVEQGDEKFSGEEEEEEEDEELGGEEGFRDFSREDSESPMKERRRYENREVELLGDWQSGGRGKE

> AtSAM4_aIDR

MYADKLEAESGSRKVVKDRINGGSGDISTRVRQVTGKRQRQDDKWEHDLFSSDKPQLSNRRVDPRDLRLKLQKRHHGSQSGREAGSGVRDLRDQLSGTMNQQPKNSDPPKSKAEAARPSMKSVATETETRKTSSQATRKKSQ

> AtSAM9_aIDR

MYSDLVVAETKISKTFKNRLNGGSGDFSSRGKQQQVTRKRGRQDDDKWEHDLFEDDDEPRLSKRRVDPKDLRLKLQKKRHGSQIGGRVFSVSVADLRDKLSRTVNPQTKNSKREAVRPAIKKVSVGTKPETRAAPNRATKKDPQ

> AtSAM2A_aIDR

MSEGSRRSRVTITLGRSGQVVNRAASDIDDGYELPRVGTKRSVKERLGNPLDSSVYGGEEVVSKRQRGEASFSGNDLQISRNDLRFKLMQKNAQRRAQSDEGCTMDLREKLSRSEQPPRSLDTRPRMAEPRDRPLSSSRTARGSSQMISSSSSHPAWALEDLRRRSPERFVDTSRGRSPPRNAGSFIGIPREPSPPRNGRRMIGSARDRSPPRSDGRIIGSPRDISPPRDAGRRFGPPRDQSPPRNAGRVTGSPRDRSPPRNAGRRMGPPRDQSPPRSTRSFSSNSRALSPARNMGSYMSSSQGFSPPRNPRSYMGSSRGSPPRSNIEDFHGRSRMLDNMRASPYPVRGVLNGQAPSSGAPFARPMLPPPVPNPHPLPPLSQLPPLGSIMQNSPFSVDEP

> AtSAM3_aIDR-L

MSNTVEDDDDDFQIPPSSQLSIRKPLHPTNANNISHRPPNKKPRLCRYPGKENVTPPPSPDPDLFCSSSTPHCILDCIPSSVDCSLGDFNGPISSLGEEDKEDKDDCIKVNREGYLCNSMEARLLKSRICLGFDSGIHEDDEGFVESNSELDVLINLCSESEGRSGEFSLGKDDSIQCPLCSMDISSLSEEQRQVHSNTCLDKSYNQPSEQDSLRKCENLSSLIKESIDDPVQLPQLVTD

> AtSAM3_aIDR-R

FASSAEVQAQSHCTSGHVTERQRDKSTTRKASEPKKPTANKLITEFFPGQATEGTKIRTAPKPVAEKSPSDSSSRRAVRRNGNNGKSKVI

> HsANKS1A_aIDR-L

MTGKRSTKEVDKTPPPQPPLISSMDSISQKSQGDVEKAVTELIIDFDANAEEEGPYEALYNAISCHSLDSMASGRSSDQDSTNKEAEAAGVKPAGVRPRERPPPPAKPPPDEEEEDHIDKKYFPLTASEVLSMRPRIHGSAAREEDEHPYELLLTAETKKVVLVDGKTKDHRRSSSSRSQDSAEGQDGQVPEQFSGLLHGSSPVCEVGQDPFQLLCTAGQSHPDGSPQQGACHKASMQLEETGVHAPGASQPSALDQSKRVGYLTGLPTTNSRSHPETLTHTASPHPGGAEEGDRSGARSRAPPTSKPKAELKLSRSLSKSDSDLLTCSPTEDATMGSRSESLSNCSIGKKRLEKSPSFASEWDEIEKIMSSIGEGIDFSQERQKISGLR

> HsANKS1A_aIDR-R

DRPYEEPPQKPPRFSQLRCQDLLSQTSSPLSQNDSCTGRSADLLLPPGDTGRRRHDSLHDPAAPSRAERFRIQEEHREAKLTLRPPSLAAPYAPVQSWQHQPEKLIFESC

> HsARAP3_aIDR

LDPKSDSAMEPSPSPAPQAQPPKPVPKPRTVFGGLSGPATTQRPGLSPALGGPGVSRSPEPSPRPPPLPTSSSEQSSALNTVEMMPNSIYFGLDSRGRAQAAQDKAPDSSQISAPTPALRPTTGTVHIMDPGCLYYGVQPVGTPGAPDRRESRGVCQGRAEHRLSRQDLEAREDAGYASLELPGDSTLLSPTLETEETSDDLISPYASFSFTADRLTPLL

> HsEPHB2_aIDR

IQSVEGQPLARRPRATGRTKRCQPRDVTKKTCNSNDGKKKGMGKKKTDPGRGREIQGIFFKEDSHKESNDCSCGG

> HsSMBT1_aIDR

DKCSENCSVLTKTKYTHYYGKKKNKRIGRPPGGHSNLACALKKASKRRKRRKNVFVHKKKRSSASVDNTPAGSPQGSGGEDEDDPDEGDDDSLSEGSTSEQQDELQEESEMSEKKSCSSSPTQSEISTSLPPDRQRRKRELRTFSFSDDENKPPSPKEIRIEVAERLHLD

> HsSMBT2_aIDR

SPVLISENCPENCSIHTKTKYTYYYGKRKKISKPPIGESNPDSGHPKPARRRKRRKSIFVQKKRRSSAVDFTAGSGEESEEEDADAMDDDTASEETGSELRDDQTDTSSAEVPSARPRRAVTLRSGSEPVRRPPPERTRRGRGAPAASSAEEGEKCPPTKPEGTEDTKQEEEERLVLESNPLEWTVTDVVRFIKLTDCAPLA

> HsLCP2_aIDR

EINKNEERRSIFTRKPQVPRFPEETESHEEDNGGWSSFEEDDYESPNDDQDGEDDGDYESPNEEEEAPVEDDADYEPPPSNDEEALQNSILPAKPFPNSNSMYIDRPPSGKTPQQPPVPPQRPMAALPPPPAGRNHSPLPPPQTNHEEPSRSRNHKTAKLPAPSIDRSTKPPLDRSLAPFDREPFTLGKKPPFSDKPSIPAGRSLGEHLPKIQKPPLPPTTERHERSSPLPGKKPPVPKHGWGPDRRENDEDDVHQRPLPQPALLPMSSNTFPSRSTKPSPMNPLPSSHMPGAFSESNSSFPQSASLPPYFSQGPSNRPPIRAEGRNFPLPLPNKPRPPSPAEEENSLNEEWYVSYITRP

> HsSAM11_aIDR

MSKGILQVHPPICDCPGCRISSPVNRGRLADKRTVALPAARNLKKERTPSFSASDGDSDGSGPTCGRRPGLKQEDGPHIRIMKRRVHTHWDVNISFREASCSQDGNLPTLISSVHRSRHLVMPEHQSRCEFQRGSLEIGLRPAGDLLGKRLGRSPRISSDCFSEKRARSESPQEALLLPRELGPSMAPEDHYRRLVSALSEASTFEDPQRLYHLGLPSHGEDPPWHDPPHHLPSHDLLRVRQEVAAAALRGPSGLEAHLPSSTAGQRRKQGLAQHREGAAPAAAPSFSERELPQPPPLLSPQNAPHVALGPHLRPPFLGVPSALCQTPGYGFLPPAQAEMFAWQQELLRKQNLARLELPADLLRQKELESARPQLLAPETALRPNDGAEELQRRGALLVLNHGAAPLLALPPQGPPGSGPPTPSRDSARRAPRKGGPGPASARPSESKEMTGARLWAQDGSEDEPPKDSDGEDPETAAVGCRGPTPGQAPAGGAGAEGKGLFPGSTLPLGFPYAVSPYFHTGAVGGLSMDGEEAPAPEDV

> HsSMAD7_aIDR

MAVNPLLTPTGQQTIPLIPSPFGPPTVDRDVLPSTVAPTDPRQFCVPSQFGSSVLPNTNMANVLSSRIYPGWGILPPESIKAVARRNEMIQRHHTARTEMEMYAIYQQRRMEKINPKGLAGLGIPFLYGSSVPAAPAAYHGRSMLPAGDLHFHRSTLRNLQGNPMLAATAPHFEESWGQRCRRLRKNTGNQKALDSDAESSKSQAEEKILGQTHAVPYEEDHYAKDPDIEAPSNQKSSETNEKPTTALANTCGELEPTHRKPWGSHTTTLKAKAWDDGKEEASEQIFATCDEKNGVCPPVPRPSLPGTHALVTIGGNLSLDEDIQ

> HsSHIP2_aIDR

IDKDEAGAKSKAPSVSRGSQEPRSGSRKPAFTEASCPLSRLFEEPEKPPPTGRPPAPPRAAPREEPLTPRLKPEGAPEPEGVAAPPPKNSFNNPAYYVLEGVPHQLLPPEPPSPARAPVPSATKNKVAITVPAPQLGHHRHPRVGEGSSSDEESGGTLPPPDFPPPPLPDSAIFLPPSLDPLPGPVVRGRGGAEARGPPPPKAHPRPPLPPGPSPASTFLGEVASGDDRSCSVLQMAKTLSEVDYAPAGPARSALLPGPLELQPPRGLPSDYGRPLSFPPPRIRESIQEDLAEEAPCLQGGRASGLGEAG

> HsETV6_aIDR

PRILFSPFFHPGNSIHTQPEVILHQNHEEDNCVQRTPRPSVDNVHHNPPTIELLHRSRSPITTNHRPSPDPEQRPLRSPLDNMIRRLSPAERAQGPRPHQENNHQESYPLSVSPMENNHCPASSESHPKPSSPRQESTRVIQLMPSPIMHPLILNPRHSVDFKQSRLSEDGLHREGKPINLSHREDLAYMNHIMVSVSPPEEHAMPIGRIADCRL

> HsPHC1_aIDR

KRYNVSCSHQFRLKRKKMKEFQEANYARVRRRGPRRSSSDIARAKIQGKCHRGQEDSSRGSDNSSYDEALSPTSPGPLSVRAGHGERDLGNPNTAPPTPELHGINPVFLSSNPSRW

> HsPHC2_aIDR

KCELCGRVDFAYKFKRSKRFCSMACAKRYNVGCTKRVGLFHSDRSKLQKAGAATHNRRRASKASLPPLTKDTKKQPTGTVPLSVTAALQLTHSQEDSSRCSDNSSYEEPLSPISASSSTSRRRQGQRDLELPDMHMRDLVGMGHHFLPSE

> HsPHC3_aIDR

RYNVSCSKKFALSRWNRKPDNQSLGHRGRRPSGPDGAAREHILRQLPITYPSAEEDLASHEDSVPSAMTTRLRRQSERERERELRDVRIRKMPENSDLLPVAQTEPSIWT

> HsSAMD9_aIDR

DSIQTSKMGKPSKNAPKDQTVSQKERRETSKQKQKGKENPDMANPSAMSTTAKGSKSLKVELIEDKIDYTKERQPSIDLT

**IDRs adjacent to DIX domain** (Supports Supplementary Fig. S10B)

> HsAXIN2_aIDR

RSGGENTAYMSNGGLGSLKVVCGYLPTLNEEEEWTCADFKCKLSPTVVGLSSKTLRATASVRSTETVDSGYRSFKRSDPVNPYHIGSGYVFAPATSANDSEISSDALTDDSMSMTDSSVDGIPPYRVGSKKQLQREMHRSVKANGQVSLPHFPRTHRLPKEMTPVEPATFAAELISRLEKLKLELESRHSLEERLQQIREDEEREGSELTLNSREGAPTQHPLSLLPSGSYEEDPQTILDDHLSRVLKTPGCQSPGVGRYSPRSRSPDHHHHHHSQYHSLLPPGGKLPPAAASPGACPLLGGKGFVTKQTTKHVHHHYIHHHAVPKTKEEIEAEATQRVHCFCPGGSEYYCYSKCKSHSKAPETMPSEQFGGSRGSTLPKRNGKGTEPGLALPAREGGAPGGAGALQLPREEGDRSQDVWQWMLESERQSKPKPHSAQSTKKAYPLESARSSPGERASRHHLWGGNSGHPRTTPRAHLFTQDPAMPPLTPPNTLAQLEEACRRLAEVSKPPKQRCCVASQQRDRNHSATVQTGATPFSNPSLAPEDHKEPKKLAGVHALQ

> HsAXIN1_aIDR

TRTGSESPKVCSDQSSGSGTGKGISGYLPTLNEDEEWKCDQDMDEDDGRDAAPPGRLPQKLLLETAAPRVSSSRRYSEGREFRYGSWREPVNPYYVNAGYALAPATSANDSEQQSLSSDADTLSLTDSSVDGIPPYRIRKQHRREMQESVQVNGRVPLPHIPRTYRVPKEVRVEPQKFAEELIHRLEAVQRTREAEEKLEERLKRVRMEEEGEDGDPSSGPPGPCHKLPPAPAWHHFPPRCVDMGCAGLRDAHEENPESILDEHVQRVLRTPGRQSPGPGHRSPDSGHVAKMPVALGGAASGHGKHVPKSGAKLDAAGLHHHRHVHHHVHHSTARPKEQVEAEATRRAQSSFAWGLEPHSHGARSRGYSESVGAAPNASDGLAHSGKVGVACKRNAKKAESGKSASTEVPGASEDAEKNQKIMQWIIEGEKEISRHRRTGHGSSGTRKPQPHENSRPLSLEHPWAGPQLRTSVQPSHLFIQDPTMPPHPAPNPLTQLEEARRRLEEEEKRASRAPSKQRYVQEVMRRGRACVRPACAPVLHVVPAVSDMELSETETRSQRKVGGGSAQPC

> HsDIXC1_aIDR

TNYNSHNSQSNGFLLPTAGKGATSVSNRGTSDLQLVRDALRSLRNSFSGHDPQHHTIDSLEQGISSLMERLHVMETQKKQERKVRVKSPRTQVGSEYRESWPPNSKLPHSQSSPTVSSTC

> HsDVL2_aIDR

NPQPEMAPPVHEPRAELAPPAPPLPPLPPERTSGIGDSRPPSFHPNVSSSHENLEPETETESVVSLRRERPRRRDSSEHGAGGHRTGGPSRLERHLAGYESSSTLMTSELESTSLGDSDEEDTMSRFSSSTEQSSASRLLKRHRRRRKQRPPRLERTSSFSSVTDSTMSLNI

> HsDVL1_aIDR

GAHSDAGSQGTDSHTDLPPPLERTGGIGDSRPPSFHPNVASSRDGMDNETGTESMVSHRRERARRRNREEAARTNGHPRGDRRRDVGLPPDSASTALSSELESSSFVDSDEDGSTSRLSSSTEQSTSSRLIRKHKRRRRKQRLRQADRASSFSSITDSTMSLNIV

> HsDVL3_aIDR

AEGSHPDPAPFCADNPSELPPPMERTGGIGDSRPPSFHPHAGGGSQENLDNDTETDSLVSAQRERPRRRDGPEHATRLNGTAKGERRREPGGYDSSSTLMSSELETTSFFDSDEDDSTSRFSSSTEQSSASRLMRRHKRRRRKQKVSRIERSSSFSSITDSTMSL

> AtSOSEK1_aIDR

SSPKEDYPNVEKKAWVTRNGGIDAEEKLQKLKLTSEKIQKESPVFCSQRSTATTSTVTEESTTNEEGFVLKKQDPKTVSGQRDGSTENGSGNDVESGRPSVSSTTSSSSYIKNKSYSSVRASHVLRNLMKCGGLDTNDAVLVPLNKSRSGAFGPAWEDERRYQYHQQHNARKSFEGAWSGIKMKETIEFCKPKVAPSKPSMAPLCSQCGKLFKPEKMHSHMKLCRGMKNSSANNDLMTSNNTVKPRQQRCRNIPGNPLGHQRVLTTTLKE

> AtSOSEK2_aIDR

KFQEVHVNRPLSGSIQEAPKSRLLRSKLKPQNRTASFDDAELYVGEEEEEEDGEYELYEEKTSYTSSTTPQSRCSRGVSTETMESTEQKPNLTKTEQDLQVRSDSSDLTRSNPVVKPRRHEVSTRVEDGDPVEPGSGRGSMWLQMISCGHIATKYYAPSVMNPRQKEENLRKGVLCKNIVKKTVVDDEREMIRFMSENPRFGNPQAEEKEYFSGSIVESVSQERVTAEPSLRRSNSFNEERSKIVEMAKETKKKEERSMAKVKCIPRTCLMSSSKQIKK

> AtSOSEK3_aIDR

SNSDHFSPIVNLATQNMKQIVVEPPSSRSMDDSSSSSSMNNGKGTNKHSHEDDELSPPALRSVSSSGVSPDSRDAKNSSSWCLAEYKVYKSEGLADASTQTDETVSGRSKTPIETFSRGVSTDEDVSSEPETSENNLVSEASCAGKERESAEISRNSVSPPFSNSASSLGGKTDTLESLIRADVSKMNSFRILEQEDVRMPAIPRLRASNMLMQLISCGSISVKDNNFGLVPTYKPKFGHSKFPSPFFSSSFMMGDLDRLSETPSLMGLRMEEKEYFSGSLVETKLQKKDAADSNASLKRSSSYNGDRASNQMGVAENGDSKPDSSKNNPSSRKASSILGKQQPLVSEKRRDSSEDTTKNIPCTTKTHDACSKRITESLRKPDSFREDEERVIKIDERLASGARVRIESKVPSEEP

> AtSOSEK4_aIDR

LDNNSGNFSAVTHRRNQSWSSVDHYKVYKASELNAEATRKLSMDASTQTDDRRRRKSPVDEVNEVTELSREEITSPPQSDSSPETLESLMRADGRLILLQEDQELNRTVEKMRPSAVLMQLISCGAMSFKKCGPTLMNGNTRSTAVRGTGNYRLERAEKELKSFGRVKLEEKEYFSGSLIDESSKKELVPALKRSSSYNIDRSSRMGLTKEKEGEELARANFIPRNPNSVVGQP

> AtSOSEK5_aIDR

SCLISNPRSLLETSSFRDPRSLNPDKNSGDDIPAVINRRRNQSWSSIDLSEYKVYKATESSAESTQRLAADASTQTDDRRRRRRPAKEEIEEVKSPASYENQSTELSRDEISPPPSDSSPETLENLIKADGRLILRPSESSTDHRTVESLSSGRMRASAVLMQLISCGTMSFKECGPVLLKDQGLALNGRSGCTITRGAEDNGEERVDKELKSFGRVQLEDKEYFSGSLIETKKELVPALKRSSSYNADRCSRMGPTTEKDEEEAVRAKCIPRKPKPVAKRNNGGQQ
