# Supplementary material for: Polymerization-mediated SRFR1 condensation in upper lateral root cap cells regulates root growth
Source: Plant Cell. 2025 Dec 30;38(1):koaf292. doi: 10.1093/plcell/koaf292 (PMC12862871; doi:10.1093/plcell/koaf292)
Supplement: koaf292_Supplementary_Data [file koaf292_supplementary_data.zip › Supplementary Video legends.docx]

**Supplementary Video. S1.** SRFR1 forms condensates in upper lateral root cap cells. (Supports Fig. 1J) Single-section images were taken along the z-axis using a Leica SP8 confocal microscope from the roots of 6-day-old *srfr1-1 YFP-HA-gSRFR1^WT^ #7* seedlings grown on ½ MS agar plate. The images were then processed with Huygens Confocal Deconvolution software. Images were then displayed sequentially from the surface to the median section using iMovie software.

**Supplementary Video. S2.** SRFR1 forms condensates in upper lateral root cap cells. (Supports Fig. 1L) Single section images along the z-axis were taken using a Leica SP8 confocal microscope from the roots of 6-day-old *srfr1-1 YFP-HA-gSRFR1^WT^ #7* seedlings grown on ½ MS Agar plate. Images were then processed with Huygens Confocal Deconvolution software. A 3D rotating movie were generated with Leica Application Suite X software.

**Supplementary Video. S3.** The PANT domain forms fibrils in Arabidopsis and rice protoplasts. (Supports Fig. 3B) 3D rotating movies showing PANT fibrils in one Arabidopsis protoplast and three rice protoplasts. YFP-tagged PANT protein transiently expressed in Arabidopsis and rice protoplasts for 16 hours. Single section images along the z-axis were taken using a Leica SP8 confocal microscope and processed with Huygens Confocal Deconvolution software. The 3D rotating movies were generated with Leica Application Suite X software.

**Supplementary Video. S4.** Purified GST-YFP-PANT forms fibrils *in vitro*. (Supports Fig. 4B) 3D rotating movie showing GST-YFP-PANT fibrils formed in PBS buffer 3 hours post PEG addition. Single section images along the z-axis were taken using a Leica SP8 confocal microscope and processed with Huygens Confocal Deconvolution software. A 3D rotating movie were generated with Leica Application Suite X software.
